# Identification of Tetrahydrocannabidiol Metabolites in Human Urine

**DOI:** 10.1002/dta.3945

**Published:** 2025-09-02

**Authors:** Willi Schirmer, Isabelle Mösch, Stefan Schürch, Wolfgang Weinmann

**Affiliations:** ^1^ Institute of Forensic Medicine, Forensic Toxicology and Chemistry University of Bern Bern Switzerland; ^2^ Department of Chemistry, Biochemistry and Pharmaceutical Sciences University of Bern Bern Switzerland

## Abstract

Tetrahydrocannabidiol (H4CBD) is an emerging semisynthetic cannabinoid, which has been known since 1940. Like hexahydrocannabinol (HHC), it is easily obtained by hydrogenation of available phytocannabinoids, in the case of H4CBD by hydrogenation of cannabidiol (CBD). H4CBD shows a weak affinity for the CB_1_ receptor, but it is unclear if H4CBD shows psychoactive properties, as reports from users are divided. Only a few countries have placed H4CBD under their narcotic substance law, for example, France and Switzerland. The aim of this study was to identify human Phase I and II metabolites in urine as potential forensic targets. The H4CBD used for this study was bought from an online store and analyzed beforehand using GC–MS. The Phase I and II metabolites were identified using LC‐HR‐MS/MS and GC–MS after trimethylsilylation. The found H4CBD metabolites were carboxylated, hydroxylated, and bishydroxylated species and their glucuronides with hydroxylation and carboxylation positions on the alicyclic moiety and on the side chain. The tentatively identified metabolites were the carboxylic acids 5″‐COOH‐H4CBD and 7‐COOH‐H4CBD, the hydroxylated metabolites (1*R*,6*R*)‐OH‐H4CBD, (1*R*,6*S*)‐OH‐H4CBD, two epimers of 2″‐OH‐H4CBD, and both epimers of 7‐OH‐H4CBD. The identified bishydroxylated metabolites were side‐chain hydroxylated derivatives of 7‐OH‐H4CBD. Various other hydroxylated metabolites were found, but their exact hydroxylation positions could not be determined. Some ESI+ spectra of the metabolites showed very unusual fragmentation patterns, like the loss of both oxygens from the resorcinol moiety with subsequent ring contraction and the appearance of radical cations for Phase II metabolites. These unusual patterns were noticed for H4CBD and its side‐chain‐altered metabolites.

## Graphical abstract

The detection of Phase I and II metabolites of tetrahydrocannabidiol (H4CBD) in urine after oral ingestion of 25‐mg H4CBD by a volunteer is described. The proposed metabolite structures are based on ESI+ spectra recorded by HPLC‐HRMS and EI spectra after trimethylsilylation recorded by GC–MS.

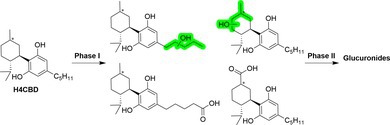

## 1 Introduction

Tetrahydrocannabidiol (H4CBD, also named H4‐CBD or THD) is a fully hydrogenated derivative of cannabidiol (CBD) and was first described in 1940. Jacob and Todd isolated CBD from Egyptian hashish and observed that CBD reacts with two equivalents of hydrogen, indicating the two nonaromatic double bonds of CBD [1]. With the rise and fall of hexahydrocannabinol (HHC) in Europe, other semisynthetic cannabinoids emerged to replace the widely banned HHC. H4CBD is one of these successors, which is easily obtained by hydrogenation of CBD. From CBD, it is known that it acts as a negative allosteric modulator at the CB_1_ receptor, acting antagonistically [2]. In contrast to CBD, H4CBD (epimeric mixture of (*R*)‐ and (*S*)‐H4CBD) has a weak affinity of 145 nM at the CB_1_ receptor [3]. Δ^9^‐Tetrahydrocannabinol (Δ^9^‐THC), the main psychoactive phytocannabinoid, in comparison, has an affinity of 40.7 nM at CB_1_ [4]. It is therefore assumed that H4CBD might have cannabimimetic effects in higher doses. However, a recent study showed that neither of the H4CBD epimers showed significant CB_1_ activation in comparison to Δ^9^‐THC. (*S*)‐H4CBD showed the strongest CB_2_ activation of the screened semisynthetic cannabinoids, whereas (*R*)‐H4CBD showed no significant CB_2_ activation in comparison with CP55,940 [5]. User reports from drug forums are inconsistent. Some users noted mild psychoactive effects, whereas others reported no effects at all after consumption. It is not clear if the users who noticed psychoactive effects consumed solely H4CBD, as these products are often wrongly declared [6, 7]. In a recent study in Germany in which 79 confiscated samples were analyzed, H4CBD was the most frequently detected semisynthetic cannabinoid apart from HHC [8], probably because, like HHC, it can easily be produced from CBD, whereas other semisynthetic cannabinoids are not obtained from phytocannabinoids [9, 10, 11]. Products containing H4CBD were also reported in Japan and Denmark [7, 12]. Switzerland scheduled H4CBD under its narcotic substance law directory on October 9, 2023 [13].

In analogy to the metabolism of Δ^9^‐THC, CBD undergoes hydroxylation on the allylic methyl group at C7 to form the metabolite 7‐OH‐CBD, which is further oxidized to the corresponding acid 7‐COOH‐CBD. These metabolites were found in rat liver after in vitro metabolism and after in vivo experiments in mice liver after application of a CBD suspension [14, 15]. The same metabolites were later found in the urine of a dystonic patient who received treatment with CBD [16]. 7‐OH‐CBD and 7‐COOH‐CBD are commonly used as analytical targets after the consumption of CBD [17, 18, 19]. The aim of this study was to identify urinary Phase I and II metabolites of H4CBD and provide analytical targets for the proof of consumption.

## 2 Materials and Methods

H4CBD was bought from a Swiss online shop before the substance was banned. The product was declared as pure H4CBD and was a reddish resin. It contained 34% (*R*)‐H4CBD and 23% (*S*)‐H4CBD, quantified by GC–MS using a single five‐point calibration (see Figure S1). Deionized water (18.2 MΩ·cm) was produced from a Millipore Milli‐Q IQ 7000 system (Billerica, MA, United States). *N*‐methyl‐*N*‐trimethylsilyltrifluoracetamide (MSTFA) (≥ 98.5%), *n*‐butyl acetate (*n*‐BuOAc) (≥ 99.7%), the *n*‐alkane standard (C7‐C40, 1000 μg/mL), and ammonium formate (≥ 99.0%) were purchased from Sigma‐Aldrich (Buchs, Switzerland). Ethyl acetate (EtOAc) (for liquid chromatography) and methanol (MeOH) (≥ 99.9%) were purchased from Carl Roth (Karlsruhe, Germany). Formic acid (50%, in water) and acetic acid (AcOH) were purchased from Grogg Chemie (Stettlen, Switzerland). Acetonitrile (MeCN) (≥ 99.9%) was purchased from Thermo Fisher Scientific (Reinach, Switzerland). Chromabond C18 SPE cartridges (3 mL, 500 mg) were purchased from Macherey‐Nagel (Önsingen, Switzerland). (*R*)‐Tetrahydrocannabidiol ((*R*)‐H4CBD) and (*S*)‐tetrahydrocannabidiol ((*S*)‐H4CBD) were purchased from Cayman Chemical (Ann Arbor, MI, United States). The internal standard (−)‐Δ^9^‐*trans*‐tetrahydrocannabinol‐D_3_ (THC‐D_3_) was purchased from Cerilliant (Round Rock, TX, United States). Instant buffer I and *β*‐glucuronidase (BGTurbo) from Finden KURA were used, which were purchased from Specialty Diagnostix (Passau, Germany). An aqueous solution of MeCN (60 V%) and formic acid (0.1 V%) was used for the reconstitution of LC–MS samples.

### 2.1 Self‐Administration Experiment

In a self‐experiment, a cannabis‐abstinent volunteer (60‐year‐old male) orally ingested 25 mg of a well‐characterized H4CBD product after dissolving it in olive oil. Urine samples were collected for 3 days after ingestion, and the first sample was collected 1 h after ingestion. Further samples were collected 3, 13, 17, 27, and 48 h after oral ingestion. The described metabolites were detected in the urine sample 3 h after consumption. No cannabimimetic effects were noticed.

### 2.2 LC‐QqTOF

Urine sample preparation was performed according to Schirmer et al. [20]. For the analysis of Phase I metabolites, 800‐μL urine, 100‐μL instant buffer I, and 5‐μL *β*‐glucuronidase were incubated for 15 min at 50°C. The solution was extracted with 1‐mL *n*‐BuOAc by shaking it for 10 min, centrifuging for 10 min (13,000 rpm [17,190 *g*], 8°C), and separating the organic layer. The organic phase was evaporated to dryness at 50°C under a stream of nitrogen and reconstituted in a 100‐μL reconstitution solution. For the analysis of Phase II metabolites, 800‐μL urine was mixed with 100 μL of an ammonium formate (10 M) solution and extracted with 1‐mL cold MeCN. The organic layer was evaporated to dryness and dissolved with 200‐μL reconstitution solution. Samples were analyzed on a Dionex Ultimate 3000 HPLC system (Thermo Fisher Scientific, Reinach, Switzerland), which was coupled to a TripleTOF 5600 mass spectrometer (Sciex, Toronto, Canada). For the data acquisition, Analyst TF software (Version 1.7) was used, and data processing was performed with Peak View (Version 1.2.0.3) and Sciex OS (Version 2.0.0.45330). Mass spectra were acquired in positive ionization mode (ESI+) using an IonDrive Turbo V ion source with a TurboIonSpray probe. The curtain gas and the ion source gases 1 and 2 were set to 55.0 psi, the ion spray voltage floating was 5500 V, and the source temperature was 650°C. Chromatography was performed on a Kinetex C8 column, 50 × 2.1 mm, 2.6 μm, 100 Å. For the analysis of Phase I metabolites after deglucuronidation, a gradient method consisting of mobile phase A (0.1% aqueous formic acid [%V]) and mobile phase B (MeCN with 0.1% formic acid [%V]) with the following gradient was used: 0–5 min, 45%–62% B; 5–14.5 min, 62% B; 14.5–14.6 min, 62%–45% B; and 14.6–15 min, 45% B. The flow rate was 0.3 mL/min, the injection volume was 2.5 μL, and the column oven was set to 25.0°C. For the analysis of Phase II metabolites, the gradient was changed to the following: 0–5 min, 30%–40% B; 5–14.5 min, 40% B; 14.5–14.6 min, 40%–30% B; and 14.6–15 min, 30% B. The instrument was operated in IDA (information‐dependent data acquisition) and in SWATH mode (sequential window acquisition of all theoretical mass spectra). A survey scan from *m/z* 100 to 950 was applied for IDA, which triggered the acquisition of product ion mass spectra from *m/z* 50 to 950. For SWATH mode, a mass range was scanned from *m/z* 100 to 950, acquiring product ion spectra in windows of 35 Da from *m/z* 50 to 950. A collision energy with a collision energy spread of 35 ± 15 V was used for IDA and SWATH mode [21].

## 3 LC‐QqLIT

The sample solutions after deglucuronidation as described above were analyzed using a Dionex Ultimate 3000 HPLC system (Thermo Fisher Scientific, Reinach, Switzerland) coupled to a QTRAP 4500 mass spectrometer (Sciex, Toronto, Canada), which was equipped with an IonDrive Turbo V ion source with TurboIonSpray probe. The curtain gas and the ion source gases 1 and 2 were set to 35.0 psi, the source temperature was 600°C, and the ion spray voltage was 5500 V. The column, mobile phases, injection volume, column oven temperature, flow rate, and gradient are the same as described for the analysis of deglucuronidated samples (Phase I metabolites) in the LC‐QqTOF section. Mass spectra were acquired in positive ionization mode (ESI+). A multiple reaction monitoring method was developed for the detection of H4CBD and its metabolites, and the relevant transitions with their corresponding potentials are shown in Table 1.

**TABLE 1:** MRM transitions of the LC‐QqLIT method. A dwell time of 20 ms and an entrance potential of 10 V for every transition were applied.

Q1/Da	Q3/Da	DP/V	ce/V	CXP/V	Name
318.2	196.3	88	32	7	THC‐D_3_ MRM 1
318.2	123.0	100	43	8	THC‐D_3_ MRM 2
319.2	83.0	60	24	16	H4CBD MRM 1
319.2	55.0	60	60	13	H4CBD MRM 2
319.2	181.0	60	20	9	H4CBD MRM 3
335.3	271.2	60	20	10	H4CBD‐OH MRM 1
335.3	137.0	60	20	10	H4CBD‐OH MRM 2
335.3	193.1	60	20	10	H4CBD‐OH MRM 3
335.3	191.1	60	20	10	H4CBD‐OH MRM 4
349.2	193.1	60	20	10	H4CBD‐COOH MRM 1
349.2	211.1	60	20	10	H4CBD‐COOH MRM 2
349.2	139.2	60	20	10	H4CBD‐COOH MRM 3

Abbreviations: ce: collision energy, CXP: cell exit potential, DP: declustering potential.

## 4 GC–MS

Sample preparation and analysis were performed according to Schirmer et al. [20]. Reference solutions were prepared by evaporating 50 μL of a 10‐μg/mL solution under a stream of nitrogen to dryness. To the residue, 25‐μL MSTFA and 25‐μL EtOAc were added. The solutions were incubated at 90°C for 40 min. The sample solutions were prepared by incubating 1‐mL urine with 100‐μL instant buffer I and 5‐μL *β*‐glucuronidase at 50°C for 15 min. The mixture was extracted twice with 500 μL *n*‐BuOAc by shaking for 10 min and centrifuging for 10 min (13,000 rpm [17,190 *g*], 8°C). The organic phases were combined and evaporated to dryness under a stream of nitrogen. The residue was dissolved in 1‐mL MeCN, diluted with 2‐mL water, and purified by solid‐phase extraction. The Chromabond C18 cartridges (3 mL, 500 mg) were conditioned with 2‐mL MeOH and 2‐mL AcOH (0.1 M). The sample solutions were loaded onto the cartridges, and they were washed with 1‐mL AcOH (0.1 M), 1‐mL aqueous MeCN (40 V%), and 1‐mL aqueous MeCN (70 V%). For the elution, 1.5‐mL MeCN was used, and the eluate was evaporated to dryness under a stream of nitrogen at 70°C. 25‐μL MSTFA and 25‐μL EtOAc were added, and the mixture was incubated at 90°C for 40 min. The extracts and reference solutions were analyzed using an 8890 gas chromatograph with a 7693A autosampler coupled to a 5977B mass selective detector (Agilent, Basel, Switzerland). MassHunter Workstation GCMS Data Acquisition (Version 10.1.49) was used for acquisition and Enhanced ChemStation (F.01.03.2357) (Agilent) for data analysis. A 5% phenylmethylsiloxane column (HP‐5 ms Ultra Inert, 30 m, 250 μm i.d., 0.25‐μm film thickness; Agilent J&W) was used. Helium with a constant flow of 1 mL/min was used as the carrier gas. The injection volume was 1 μL in pulsed splitless mode. The oven temperature started at 80°C and was ramped with 10°C/min to 300°C and held for 1 min, resulting in a total separation time of 23 min. The quadrupole temperature was 150°C, and the source temperature was 230°C. EI mass spectra were obtained with an ionization energy of 70 eV. The scan range was from *m/z* 40 to 650, with a scan speed of 1.562 s^−1^.

## 5 Investigated H4CBD Product

The investigated H4CBD resin “for recreational use” was analyzed by GC–MS: (*R*)‐H4CBD and (*S*)‐H4CBD were quantified using a single 5‐point calibration, and a single determination was performed. THC‐D_3_ was used as an internal standard. The quantification was run on another GC–MS device using identical instruments (Agilent 8890 gas chromatograph coupled to a 5977B mass spectrometer). This sample contained stearic acid and palmitic acid, presumably from the extraction of CBD from CBD‐rich *Cannabis* plants. In addition, not fully hydrogenated CBD was detected (H2CBD) in the product.

## 6 Results and Discussion

An overview of the Phase I and II metabolites that were identified by LC‐QqTOF can be found in Table 2. Table 2 shows the retention times and relevant ions of the metabolites. The trimethylsilyl derivatives of Phase I metabolites, which were identified by GC–MS, are found in Table 3. The Kováts indices [22], retention times, and relevant fragment ions are listed. The Kováts indices were calculated according to van Den Dool and Kratz [23].

**TABLE 2:** Chromatographic data and relevant ions of the Phase I and II metabolites of H4CBD (LC‐QqTOF). The ions are detected in ESI+ with a collision energy of 35 ± 15 V.

Metabolite	Glucuronide	*R*_*t*_/min	Relevant ions/Da
M1, (*R,S*)‐H4CBD		9.35	**319.2632**, 181.1223, 139.1481, 97.1012, 83.0855, 69.0699, 57.0699, 55.0542
M2, 7‐COOH‐H4CBD		3.12	349.2373, 331.2268, 313.2162, 303.2319, 285.2213, 227.1794, **137.1325**
M3, 5″‐COOH‐H4CBD		4.51	349.2373, 331.2268, 313.2162, 303.2319, 211.0965, 193.0859, 139.1481, **83.0855**
M4, OH‐H4CBD^a^		7.74	**335.2573**, 271.2420, 197.1172, 179.1067, 137.1325, 83.0855, 81.0699
M5, OH‐H4CBD^a^		7.13	335.2573, **271.2420**, 197.1172, 179.1067, 137.1325, 83.0855, 81.0699
M6, 2″OH‐H4CBD		4.87	335.2573, 317.2475, 261.1849, 179.1067, 137.1325, **123.0441**, 83.0855
M7, 2″OH‐H4CBD		4.56	335.2573, 317.2475, 261.1849, 179.1067, 137.1325, 123.0441, **83.0855**
M8, 7‐OH‐H4CBD		4.37	335.2573, 317.2475, **193.1223**, 181.1223, 137.1325, 123.0441, 95.0855, 81.0699
M9, 7‐OH‐H4CBD		3.64	335.2573, 317.2475, **193.1223**, 181.1223, 137.1325, 123.0441, 95.0855, 81.0699
M10, diOH‐H4CBD^b^		1.96	351.2530, 333.2424, 315.2319, 287.2369, 269.2264, 193.1223, 137.1325, **81.0699**
M11, H4CBD	M1	11.34	495.2952, 459.2741, 384.2659^•^, **319.2632**
M12, H4CBD	M1	11.82	495.2952, 459.2741, 384.2659^•^, **319.2632**
M13, 5″‐COOH‐H4CBD	M3	10.04	525.2694, 414.2401^•^, **349.2373**, 331.2268, 211.0965, 193.0859, 139.1481, 83.0855
M14, 7‐COOH‐H4CBD	M2	9.39	525.2694, 457.1857, 349.2373, 331.2268, 303.2319, **281.1536**, 193.1223, 181.1223
M15, OH‐H4CBD^a^	M6	5.07	511.2902, 400.2608^•^, 335.2573, **317.2475**, 261.1849, 179.1067
M16, OH‐H4CBD		5.79	511.2902, **335.2573**, 317.2475, 193.1223, 181.1223
M17, OH‐H4CBD		7.18	511.2902, **335.2573**, 317.2475, 193.1223, 137.1325
M18, 7‐OH‐H4CBD	M8, M9	8.59	511.2902, 443.2064, **335.2573**, 317.2475, 193.1223, 181.1223
M19, 7‐OH‐H4CBD	M8, M9	9.32	511.2902, 443.2064, **335.2573**, 317.2475, 193.1223, 181.1223
M20, OH‐H4CBD^a^		12.41	511.2902, **335.2573**, 317.2475, 299.2369, 271.2420, 197.1172, 179.1067, 137.1325
M21, OH‐H4CBD^a^		12.67	511.2902, **335.2573**, 317.2475, 299.2369, 271.2420, 197.1172, 179.1067, 137.1325
M22, OH‐H4CBD^a^		14.43	511.2902, **335.2573**, 317.2475, 299.2369, 271.2420, 197.1172, 179.1067, 137.1325
M23, diOH‐H4CBD^b^		3.51	527.2851, **351.2530**, 333.2424, 193.1223, 181.1223
M24, diOH‐H4CBD^c^		5.25	527.2851, **351.2530**, 333.2424, 137.1325
M25, diOH‐H4CBD^c^		5.67	527.2851, **351.2530**, 333.2424, 193.1223, 137.1325

*Note:* Base peak is written in bold. The corresponding ESI+ product ion spectra of the Phase I metabolites are found in Figures S3–S13, and the ESI+ product ion spectra of Phase II metabolites are shown in Figures S15–S29.

Abbreviation: *R*_*t*_: retention time.

^a^ Hydroxylation on side chain.

^b^ Two hydroxylation positions on alicyclic moiety.

^c^ One hydroxylation position on side chain and another on the alicyclic moiety.

**TABLE 3:** Chromatographic data (GC–MS) and relevant ions of the TMS derivatives of H4CBD and their metabolites.

Metabolite (trimethylsilylated)	*R*_*t*_/min	RRI	Relevant ions/Da (70 eV)
M26, (*R*)‐H4CBD	17.03	2235	462, **377**, 337
M27, (*S*)‐H4CBD	17.66	2307	462, **377**, 337
M28, 7‐COOH‐H4CBD	19.70	2560	564, **447**, 337, 317
M29, 5″‐COOH‐H4CBD	20.30	2640	564, 479, 439, 354, **214**
M30, (1*R*,6*S*)‐OH‐H4CBD	18.62	2424	550, 476, 392, **377**, 342
M31, OH‐H4CBD^a^	19.01	2473	550, **465**, 425
M32, (1*R*,6*R*)‐OH‐H4CBD	19.03	2475	550, **377**, 350, 337, 173
M33, 7‐OH‐H4CBD	19.15	2491	550, **447**, 337
M34, 7‐OH‐H4CBD	19.55	2541	550, **447**, 337
M35, OH‐H4CBD^a^	19.59	2547	550, **465**, 425
M36, diOH‐H4CBD^b^	20.86	2716	638, 535, 465, 425, **372**, 357
M37, diOH‐H4CBD^b^	21.37	2787	638, **535**, 499, 425

*Note:* Base peak is shown in bold. The corresponding EI mass spectra of the trimethylsilylated Phase I metabolites are found in Figures S30–S41.

Abbreviations: RT: retention time, RRI: relative retention index (Kováts index).

^a^ Hydroxylation on side chain.

^b^ Hydroxylation on C7 and on side chain.

### 6.1 LC‐QqTOF Phase I Metabolites

The chromatogram of an extract from a deglucuronidated urine sample 3 h after ingestion showed H4CBD (M1), carboxylated metabolites (M2 and M3), hydroxylated metabolites (M4–M9), and bishydroxylated metabolites (M10) (see Figure 1). A chromatogram of an extract from a deglucuronidated urine sample prior to H4CBD ingestion is shown in Figure S2. The corresponding spectra of H4CBD and its Phase I metabolites are found in Figures S3–S13. Reference spectra of (*S*)‐ and (*R*)‐H4CBD are shown in Figures S44 and S45.

**FIGURE 1:**
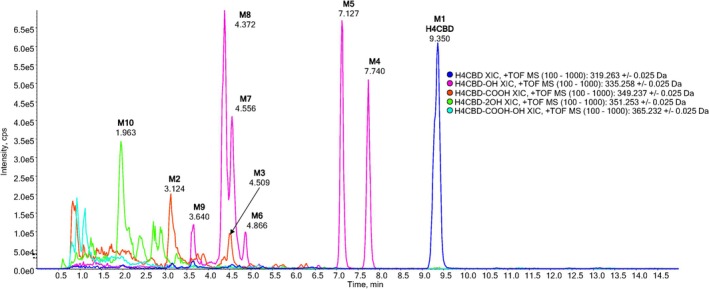
Extracted ion chromatograms of an extract from a deglucuronidated urine sample, 3 h after oral ingestion of 25‐mg H4CBD. H4CBD (blue, M1), carboxylated H4CBD (red, M2 and M3), monohydroxylated H4CBD (pink, M4–M9), and bishydroxylated H4CBD (green, M10).

Metabolite M1 is H4CBD, eluting as an unresolved double peak at 9.35 min. The ESI+ product ion spectra of (*R*)‐H4CBD and (*S*)‐H4CBD are indistinguishable and show only a few characteristic ions; see Figures S3 and S4. The base peak of the spectrum is the molecular ion (*m/z* 319.2632). The fragment ion with the highest abundance is the cyclohexylium ion (*m/z* 83.0855), which derives from the alicyclic moiety of the molecule. The most abundant ions in the spectrum of H4CBD are smaller fragments from the alicyclic moiety, which are not very characteristic, such as a butenylium ion (*m/z* 55.0542), a butylium ion (*m/z* 57.0699), a pentenylium ion (*m/z* 69.0699), and a methylcyclohexylium ion (*m/z* 97.1012). The characteristic ions are a methyl‐isopropyl‐cyclohexylium ion (*m/z* 139.1481) and a protonated olivetol ion (*m/z* 181.1223). Some very characteristic ions for CBD are not found for H4CBD, probably due to the lack of an olefinic group, which can be protonated under ESI+ conditions and initiate characteristic fragmentation patterns. The tropylium ions (*m/z* 193.1223 and *m/z* 123.0441), the former being the base peak of the CBD product ion spectrum, are absent in the spectrum of H4CBD. The ions resulting from partial fragmentation of the terpene moiety (*m/z* 259.1693 and *m/z* 217.1223), presumably chromenylium ions, are also not found in the product ion spectrum of H4CBD.

#### 6.1.1 Carboxylated Metabolites

The chromatogram of an extract from a deglucuronidated urine sample, which was collected 3 h after oral ingestion, showed two carboxylated metabolites (see Figure 1). The metabolite M2 eluting at 3.12 min shows the [M + H]^+^ ion at *m/z* 349.2373 (C_21_H_33_O_4_^+^, −0.9 ppm); see Figure S5. The ions after loss of two H_2_O at *m/z* 331.2268 (C_21_H_31_O_3_^+^, −0.9 ppm) and *m/z* 313.2162 (C_21_H_29_O_2_^+^, −3.5 ppm) and the ions after further loss of CO at *m/z* 303.2319 (C_20_H_31_O_2_^+^, 1.0 ppm) and *m/z* 285.2213 (C_20_H_29_O^+^, −3.5 ppm) can be observed. An ion with *m/z* 227.1794 (C_17_H_23_^+^, −3.5 ppm) is observed in the mass spectrum, and its formation mechanism is unknown. The base peak of the spectrum is an ion with *m/z* 137.1325 (C_10_H_17_^+^, −3.6 ppm). The ion at *m/z* 227.1794 seems to be characteristic because a similar ion (*m/z* 225.1) is found in the product ion spectrum of 7‐COOH‐CBD (see Figures S42 and S43). This metabolite was tentatively identified as 7‐COOH‐H4CBD.

The metabolite M3 eluting at 4.51 min shows the [M + H]^+^ ion at *m/z* 349.2373 (C_21_H_33_O_4_^+^, 2.6 ppm); see Figure S6. The ions after the loss of H_2_O at *m/z* 331.2268 (C_21_H_31_O_3_^+^, −0.9 ppm) and subsequent loss of CO at *m/z* 303.2319 (C_20_H_31_O_2_^+^, 4.3 ppm) or the second loss of H_2_O at *m/z* 313.2162 (C_21_H_29_O_2_^+^, −5.4 ppm) are found in the higher mass range of the spectrum. The characteristic ions are the protonated carboxy‐olivetol at *m/z* 211.0965 (C_11_H_15_O_4_^+^, −1.4 ppm) and the oxonium ion after the loss of H_2_O at *m/z* 193.0859 (C_11_H_13_O_3_^+^, −0.5 ppm). Ions resulting from the alicyclic moiety (*m/z* 139, *m/z* 97, *m/z* 83, *m/z* 69, *m/z* 57, and *m/z* 55) are identical to the ions from H4CBD, indicating as well that the side chain was metabolized. This metabolite was tentatively identified as 5″‐COOH‐H4CBD.

#### 6.1.2 Hydroxylated Metabolites

**FIGURE 2:**
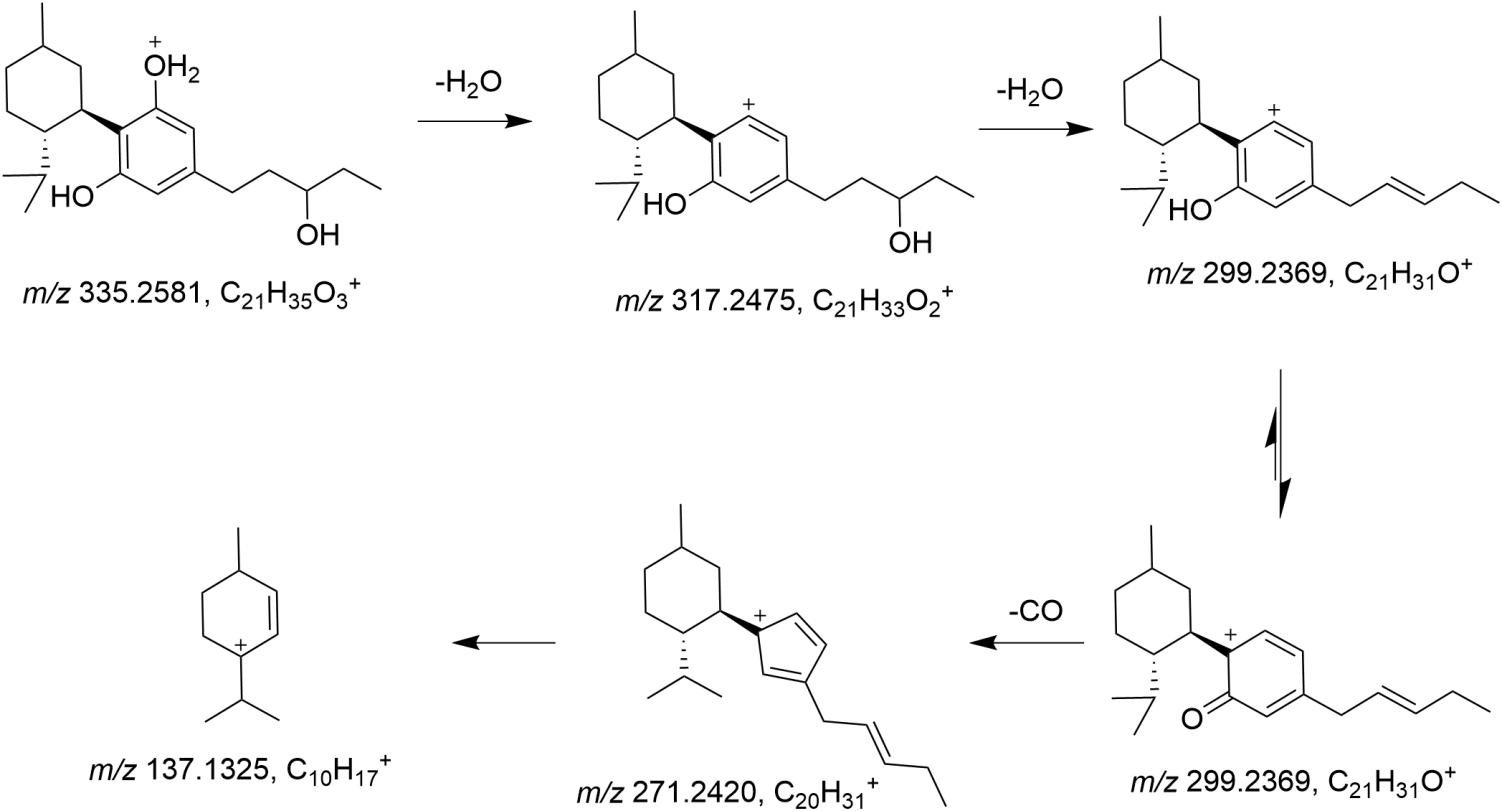
Possible fragmentation pathway of the metabolite M4, an unusual carbenium ion (*m/z* 271) is seen in the spectrum. The position of the hydroxy group on the pentyl side chain is unknown.

The metabolite M4 eluting at 7.74 min shows the [M + H]^+^ ion at *m/z* 335.2581 (C_21_H_35_O_3_^+^, 5.7 ppm); see Figure S7. An ion after the loss of a H_2_O molecule can be observed at *m/z* 317.2475 (C_21_H_33_O_2_^+^, 3.8 ppm), and an ion after a second loss of H_2_O is present at *m/z* 299.2369 (C_21_H_31_O^+^, 2.7 ppm). An ion can be observed at *m/z* 271.2420 (C_20_H_31_^+^, 4.4 ppm), and this ion results from the formal loss of CH_4_O_3_, presumably from the loss of two H_2_O and one CO. The loss of CO likely occurs from the phenol cation (*m/z* 299.2369), which tautomerizes to an oxocyclohexadienylium ion, stabilizing the positive charge on a sp^3^‐carbon. Elimination of CO from this ion leads to a ring contraction and to the formation of a cyclopentadienyl cation (*m/z* 271.2420). The ion at *m/z* 137.1325 (C_10_H_17_^+^, 4.4 ppm) results from the alicyclic part of the molecule, and this ion might form after a 1,3‐*H* shift of the cyclopentadienyl cation and subsequent elimination of the cyclopentadiene moiety. It is not an indicator for the hydroxylation position in this case. The ions at *m/z* 81.0699 (C_6_H_9_^+^, 3.7 ppm) and *m/z* 83.0855 (C_6_H_11_^+^, 2.4 ppm) result from two different processes, but their simultaneous presence is most likely indicative of a hydroxylation position on the side chain of the molecule. Two characteristic ions are found at *m/z* 197.1172 (C_11_H_17_O_3_^+^, 3.0 ppm) and its anhydrate at *m/z* 179.1067 (C_11_H_15_O_2_^+^, −1.1 ppm). These ions are a protonated olivetol with a hydroxylated side chain and its anhydrate. They are characteristic of side‐chain hydroxylated metabolites. A suggested fragmentation pathway is shown in Figure 2.

The metabolite M5 eluting at 7.13 min shows the same ions as described for the metabolite M4, and the corresponding product ion spectrum is found in Figure S8. The presence of the ions *m/z* 179 and *m/z* 197 and the absence of the ion *m/z* 181 indicate that this metabolite is hydroxylated on the side chain. The base peak *m/z* 271 indicates that this ion emerged after the loss of CO from an oxocyclohexadienylium, a tautomer of the phenol cation.

**FIGURE 3:**
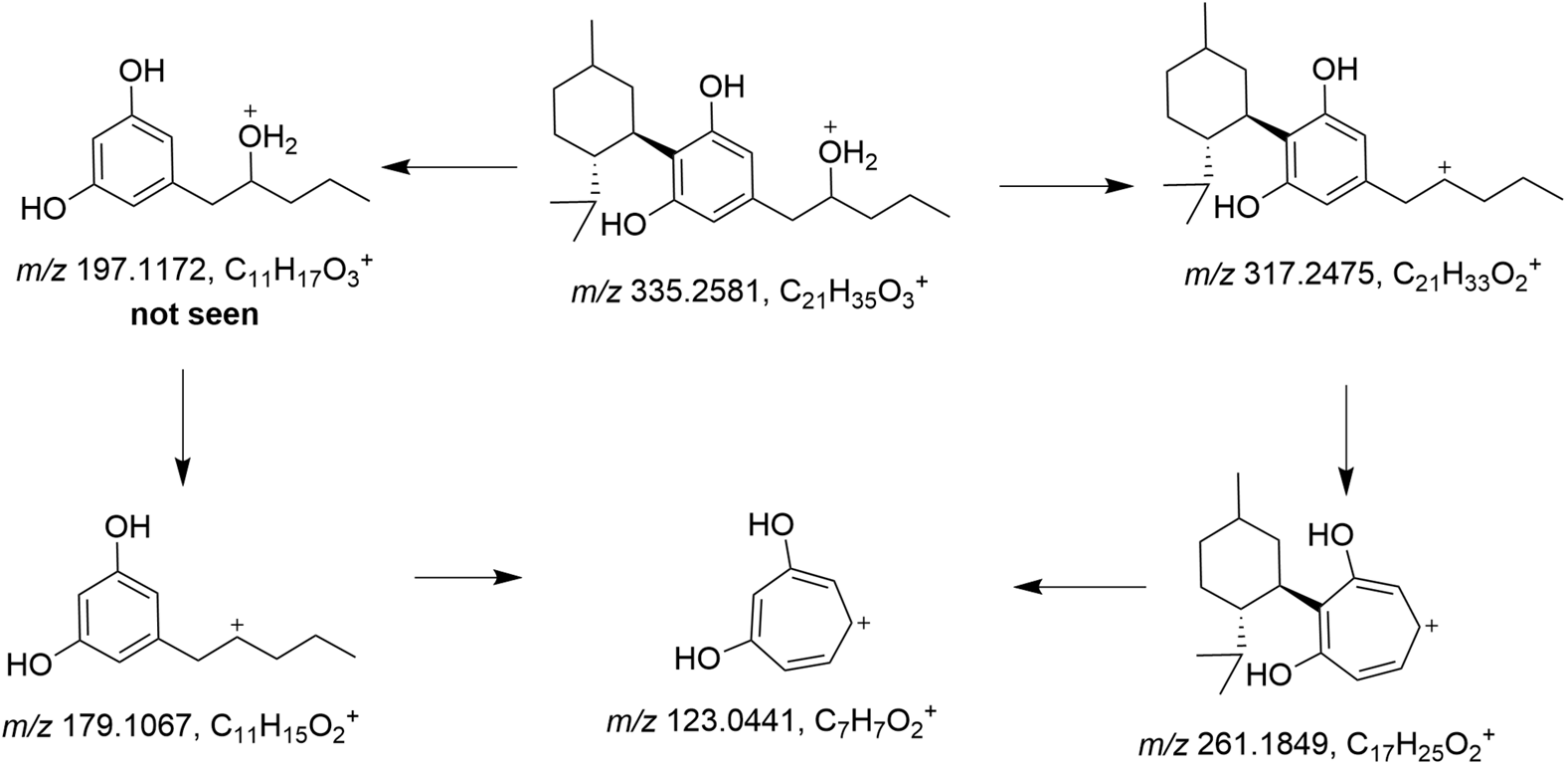
Possible fragmentation pathway of 2″OH‐H4CBD (metabolite M6).

The minor metabolite M6 eluting at 4.87 min shows the ion at *m/z* 179.1067 (C_11_H_15_O_2_^+^, 7.3 ppm), indicating that this metabolite is hydroxylated on the side chain; see Figure S9. The fragment ion at *m/z* 261.1849 (C_17_H_25_O_2_^+^, 7.3 ppm) might result from a tropylium ion, which is formed after degradation of the side chain, and a hydroxy group at C2 seems plausible. Further fragmentation of this ion leads to the tropylium ion with *m/z* 123.0441 (C_7_H_7_O_2_^+^, −2.4 ppm), which could also be formed from the ion with *m/z* 179 after elimination of *n*‐butene from the side chain. The fragment ion *m/z* 137.0592 (C_8_H_9_O_2_^+^, 8.0 ppm) is from the aromatic part. The characteristic ion *m/z* 139.1481 (C_10_H_19_^+^, −2.2 ppm) is present but very low in abundance, indicating that hydroxylation took place on the side chain. The metabolite M6 was tentatively identified as 2″OH‐H4CBD, and a suggested fragmentation pathway is shown in Figure 3.

The metabolite M7 eluting at 4.56 min shows the same ions as M6, and it is therefore assumed that these two compounds are diastereomers, showing a different configuration at either C1, the hydroxylation position C2″, or at both positions. The corresponding product ion spectrum is shown in Figure S10.

The metabolite M8 (see Figure S11) eluting at 4.37 min shows the protonated olivetol ion at *m/z* 181.1223 (C_11_H_17_O_2_^+^, 0.6 ppm), indicating a hydroxylation position on the alicyclic moiety of the molecule. In addition, a tropylium ion at *m/z* 193.1223 (C_12_H_17_O_2_^+^, 0.0 ppm) is present, which fragments further by loss of the side chain, seen at *m/z* 123.0441 (C_7_H_7_O_2_^+^, −1.6 ppm). The presence of the ion at *m/z* 193.1223 is a further indicator of the hydroxylation on the alicyclic moiety. This tropylium ion has a saturated pentyl side chain, and it can only be formed if the hydroxylation position is found on the alicyclic moiety.

A minor metabolite M9 can be seen at 3.64 min, which shows a very similar mass spectrum to the metabolite M8, probably a diastereomer. The product ion spectrum is shown in Figure S12.

#### 6.1.3 Bishydroxylated Metabolites

A bishydroxylated metabolite M10 eluting at 1.96 min shows a rather strong abundance. The presence of the ion at *m/z* 193.1223 (C_12_H_17_O_2_^+^, −1.6 ppm) indicates that this metabolite had two hydroxylation positions at the alicyclic moiety. A product spectrum is shown in Figure S13.

Some other coeluting bishydroxylated metabolites are present between 2.0 and 3.0 min, and most of them show the ions with *m/z* 193 or *m/z* 181, which are indicative ions for bishydroxylation on the alicyclic moiety.

### 6.2 LC‐QqTOF Phase II Metabolites

**FIGURE 4:**
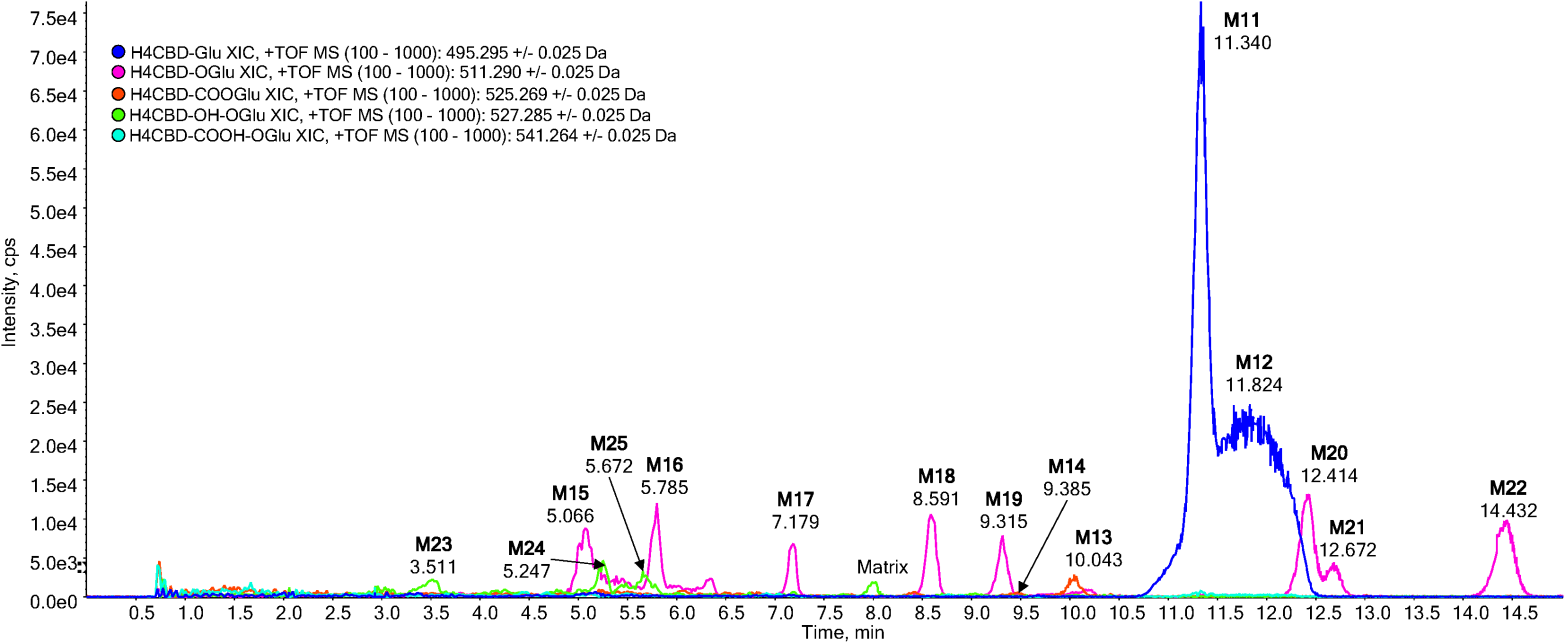
Extracted ion chromatograms of an extract from a urine sample, 3 h after oral ingestion of 25‐mg H4CBD. H4CBD glucuronide (blue, M11 and M12), carboxylated H4CBD glucuronide (red, M13 and M14), monohydroxylated H4CBD glucuronide (pink, M15–M22), and bishydroxylated H4CBD glucuronide (green, M23–M25). An extracted urine sample prior to H4CBD ingestion is shown in Figure S14.

Several glucuronidated metabolites were found in an extract of the urine sample 3 h after the ingestion of H4CBD. The most abundant metabolites in urine after 3 h were the glucuronides of H4CBD (M11 and M12). The glucuronides of carboxylated (M13 and M14), hydroxylated (M15–M22), and bishydroxylated metabolites (M23–M25) of H4CBD are present as well. A chromatogram is shown in Figure 4, and a chromatogram prior to H4CBD ingestion can be found in Figure S14.

**FIGURE 5:**
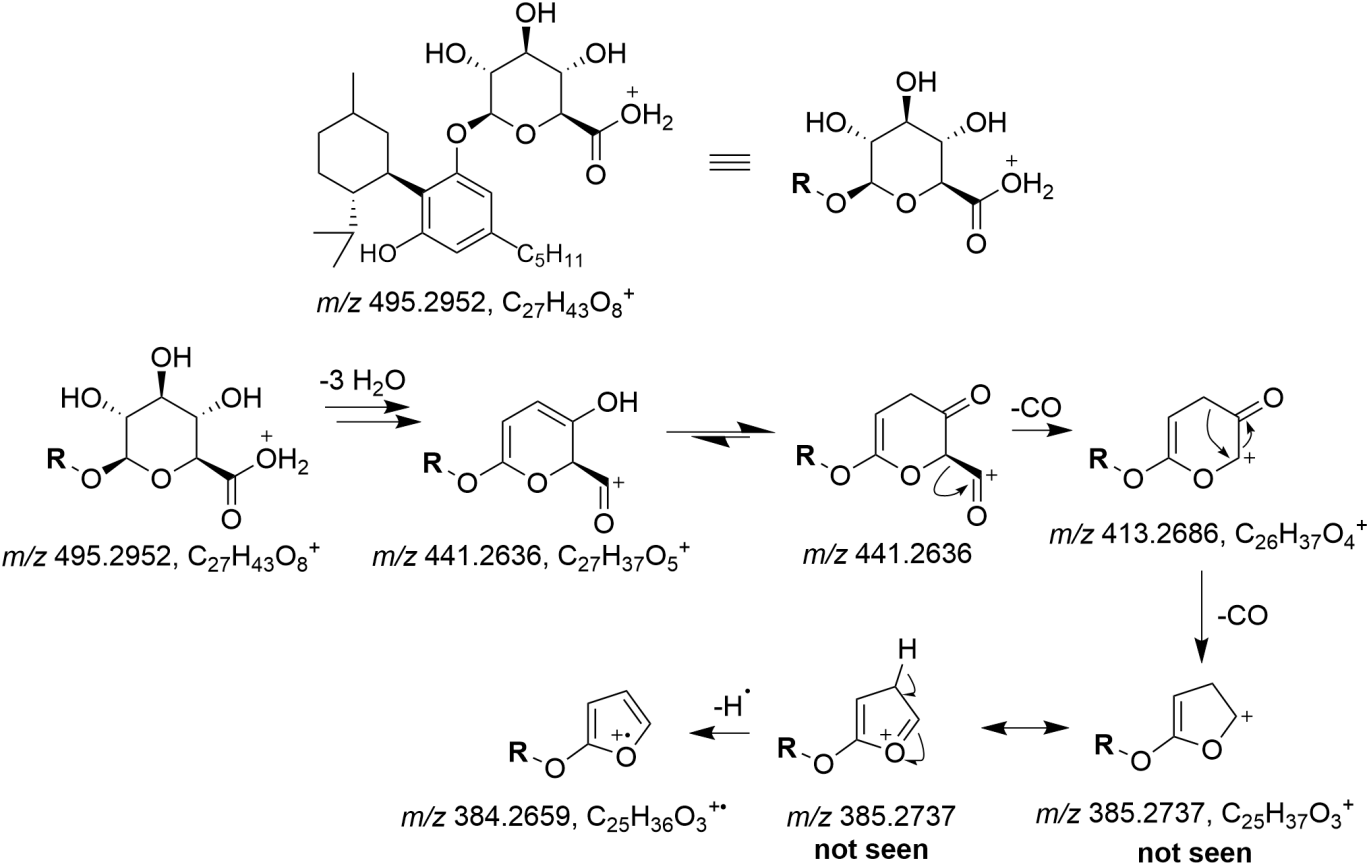
Postulated fragmentation pathway of H4CBD glucuronide, explaining the formation of the radical cation *m/z* 384.

The epimers of H4CBD glucuronide eluted at 11.34 min (M11) and 11.82 min (M12). Their product ion spectra are shown in Figures S15 and S16. The [M + H]^+^ ion can be found at *m/z* 495.2952 (C_27_H_43_O_8_^+^, −4.2 ppm), and the loss of two H_2_O molecules is seen at *m/z* 459.2741 (C_27_H_39_O_6_^+^, −2.6 ppm). An odd‐electron fragment is present at *m/z* 384.2659 (C_25_H_36_O_3_^+•^, −4.4 ppm), which results from the fragmentation of the glucuronide moiety, and a H4CBD‐furanyl radical cation seems to be a plausible structure for this fragment ion. A postulated fragmentation pathway is shown in Figure 5. The base peak of the spectrum is the protonated aglycone found at *m/z* 319.2632 (C_21_H_35_O_2_^+^, −3.1 ppm). The lower mass fragments, discussed earlier for the fragment ions of H4CBD, are of low abundance.

#### 6.2.1 Carboxylated Glucuronides

The metabolite M13 eluting at 10.04 min shows the [M + H]^+^ ion at *m/z* 525.2694 (C_27_H_41_O_10_^+^, 24.7 ppm); see Figure S17. An odd‐electron fragment ion can be seen at *m/z* 414.2401 (C_25_H_34_O_5_^+•^, 0.5 ppm), which results from the fragmentation of the glucuronide moiety. The base peak of this spectrum is the protonated aglycone at *m/z* 349.2373 (C_27_H_41_O_10_^+^, −0.9 ppm). The same lower fragment ions can be seen as in the mass spectrum of the Phase I metabolite 5″‐COOH‐H4CBD (M3), namely, *m/z* 331.2268 (C_21_H_31_O_3_^+^, 1.5 ppm), *m/z* 211.0965 (C_11_H_15_O_4_^+^, 3.3 ppm), *m/z* 193.0859 (C_11_H_13_O_3_^+^, 3.6 ppm), *m/z* 139.1481 (C_10_H_19_^+^, −6.5 ppm), and *m/z* 83.0855 (C_6_H_11_^+^, −9.6 ppm). This metabolite was tentatively identified as 5″‐COOH‐H4CBD glucuronide.

Another glucuronidated carboxylic metabolite (M14) eluted at 9.39 min, and a product ion spectrum is shown in Figure S18. The [M + H]^+^ ion is seen at *m/z* 525.2694 (C_27_H_41_O_10_^+^, 27.6 ppm). An ion at *m/z* 457.1857 (C_25_H_29_O_8_^+^, 0.2 ppm) and its deglucuronidated species at *m/z* 281.1536 (C_19_H_21_O_2_^+^, −8.9 ppm) are present, representing formal losses of C_2_H_12_O_2_ (two H_2_O and two CH_4_) from the [M + H]^+^ ion and the protonated aglycone, respectively. Their structures are unknown. Interestingly, the ion at *m/z* 281.1536 does only appear if the metabolite was priorly glucuronidated. The formation of the ion at *m/z* 169.1223 (C_10_H_17_O_2_^+^, −6.5 ppm) is unknown. It further decomposes by the loss of H_2_O at *m/z* 151.1117 (C_10_H_15_O^+^, 6.6 ppm) and the loss of CO at *m/z* 123.1168 (C_9_H_15_^+^, −4.1 ppm). Additionally, the tropylium ion at *m/z* 193.1223 (C_12_H_17_O_2_^+^, −8.8 ppm) and the protonated olivetol ion at *m/z* 181.1223 (C_11_H_17_O_2_^+^, 4.4 ppm) appear in the mass spectrum, indicating that this metabolite is carboxylated at the alicyclic moiety. This metabolite was tentatively identified as the glucuronide of 7‐COOH‐H4CBD.

#### 6.2.2 Hydroxylated Glucuronides

The metabolite M15 eluting at 5.07 min shows the [M + H]^+^ ion at *m/z* 511.2902 (C_27_H_43_O_9_^+^, −26.4 ppm); see Figure S19. Loss of three H_2_O can be observed (*m/z* 493.2796, C_27_H_41_O_8_^+^, −13.8 ppm; *m/z* 475.2690, C_27_H_39_O_7_^+^, −3.4 ppm; *m/z* 457.2585, C_27_H_37_O_6_^+^, 10.5 ppm). The characteristic odd‐electron fragment ion at *m/z* 400.2608 (C_25_H_36_O_4_^+•^, −4.2 ppm) and its anhydrate at *m/z* 382.2502 (C_25_H_34_O_3_^+•^, 4.4 ppm) can be found. The protonated aglycone is found at *m/z* 335.2581 (C_21_H_35_O_3_^+^, −1.2 ppm), and its anhydrate at *m/z* 317.2475 (C_21_H_33_O_2_^+^, −0.9 ppm) forms the base peak of this spectrum. The protonated and unsaturated olivetol ion at *m/z* 179.1067 (C_11_H_15_O_2_^+^, 1.1 ppm) is present, indicating that this metabolite was hydroxylated on the side chain. A very low‐abundant ion with *m/z* 261.1849 (C_17_H_25_O_2_^+^, −10.0 ppm) is seen. This metabolite might be the glucuronide of the metabolite M6.

The metabolite eluting M16 at 5.79 min shows the [M + H]^+^ ion at *m/z* 511.2902 (C_27_H_43_O_9_^+^, 4.7 ppm); see Figure S20. The base peak of this spectrum is the protonated aglycone at *m/z* 335.2581 (C_21_H_35_O_3_^+^, −2.4 ppm), and the anhydrate at *m/z* 317.2475 (C_21_H_33_O_2_^+^, 6.9 ppm) is also present. The tropylium ion at *m/z* 193.1223 (C_12_H_17_O_2_^+^, −3.6 ppm) and the protonated olivetol ion at *m/z* 181.1223 (C_11_H_17_O_2_^+^, 11.0 ppm) are characteristic ions for a hydroxylation position on the alicyclic moiety. The product ion spectrum is shown in Figure S20.

At 7.18 min, the metabolite M17 can be seen, and a product ion spectrum is found in Figure S21. It shows the [M + H]^+^ ion at *m/z* 511.2902 (C_27_H_43_O_9_^+^, −5.1 ppm). The protonated aglycone at *m/z* 335.2581 (C_21_H_35_O_3_^+^, 0.6 ppm) and the anhydrate at *m/z* 317.2475 (C_21_H_33_O_2_^+^, −7.9 ppm) are present, and the latter forms the base peak of this spectrum. The tropylium ion at *m/z* 193.1223 (C_12_H_17_O_2_^+^, 9.3 ppm) and the ion at *m/z* 137.1325 (C_10_H_17_^+^, −10.2 ppm) are indicative of a hydroxylation on the alicyclic moiety.

The metabolite M18, eluting at 8.59 min, shows the [M + H]^+^ ion at *m/z* 511.2902 (C_27_H_43_O_9_^+^, −2.5 ppm) and its anhydrates at *m/z* 493.2796 (C_27_H_41_O_8_^+^, 8.7 ppm) and at *m/z* 475.2690 (C_27_H_39_O_7_^+^, 0.0 ppm); see Figure S22. An ion can be seen at *m/z* 443.2064 (C_25_H_31_O_7_^+^, −8.1 ppm) representing a formal loss of C_2_H_12_O_2_, the same formal loss as observed for the glucuronidated carboxy metabolite M14. The base peak of the spectrum is the deglucuronidated molecule, seen at *m/z* 335.2581 (C_21_H_35_O_3_^+^, 2.7 ppm), and its anhydrate is seen at *m/z* 317.2475 (C_21_H_33_O_2_^+^, 5.0 ppm). The presence of the tropylium ion at *m/z* 193.1223 (C_12_H_17_O_2_^+^, −2.6 ppm), the protonated olivetol ion at *m/z* 181.1223 (C_11_H_17_O_2_^+^, −1.7 ppm), and the ion at *m/z* 137.1325 (C_10_H_17_^+^, −2.9 ppm) are indicative of a metabolite that is hydroxylated on the alicyclic moiety. The same lower fragment ions appear as in the mass spectrum of the Phase I metabolites M8 and M9. This metabolite was tentatively identified as 7‐OH‐H4CBD glucuronide.

The metabolite M19 eluting at 9.32 min shows the same fragment ions as the metabolite M18, indicating that these two metabolites are diastereomers; see Figure S23. This metabolite was therefore tentatively identified as the other epimer of 7‐OH‐H4CBD glucuronide.

The metabolite M20 eluting at 12.41 min shows the [M + H]^+^ ion at *m/z* 511.2902 (C_27_H_43_O_9_^+^, 1.6 ppm), shown in Figure S24. The protonated aglycone at *m/z* 335.2581 (C_21_H_35_O_3_^+^, 3.9 ppm) forms the base peak of the spectrum. Repetitive loss of two H_2_O molecules resulting in the ions at *m/z* 317.2475 (C_21_H_33_O_2_^+^, −0.6 ppm) and at *m/z* 299.2369 (C_21_H_31_O^+^, 4.3 ppm) is present. Further loss of CO at *m/z* 271.2420 (C_20_H_31_^+^, 1.5 ppm) is also present. The ion at *m/z* 137.1325 (C_10_H_17_^+^, −2.9 ppm) is not indicative of a hydroxylation on the alicyclic moiety in this case, as the ions at *m/z* 197.1178 (C_11_H_17_O_3_^+^, 5.1 ppm) and at *m/z* 179.1065 (C_11_H_15_O_2_^+^, −16.7 ppm) are present but not very abundant. The same lower fragment ions appear as in the mass spectrum of the Phase I metabolites M4 and M5, and the product ion spectrum of the metabolite M20 is found in Figure S24.

The mass spectrum of the metabolite M21 eluting at 12.67 min is very similar to the mass spectrum of the metabolite M20, and the same fragment ions appear in similar abundances. The product ion spectrum is found in Figure S25.

The mass spectrum of the metabolite M22 (see Figure S26) eluting at 14.43 min is similar to the mass spectra of the metabolites M20 and M21, but only the aglycone at *m/z* 335.2581 (C_21_H_35_O_3_^+^, 4.5 ppm) is of high abundance. The other fragment ions are even less abundant than in the spectra of the metabolites M20 and M21.

#### 6.2.3 Bishydroxylated Glucuronides

A glucuronidated bishydroxylated metabolite M23 eluted at 3.51 min; a product ion spectrum is found in Figure S27. The [M + H]^+^ ion at *m/z* 527.2851 (C_27_H_43_O_9_^+^, 6.6 ppm) and loss of two H_2_O molecules are seen at *m/z* 509.2745 (C_27_H_41_O_8_^+^, −4.1 ppm) and at *m/z* 491.2639 (C_27_H_39_O_7_^+^, −11.4 ppm). The protonated aglycone at *m/z* 351.2530 (C_21_H_35_O_4_^+^, 5.1 ppm) and loss of two H_2_O molecules are seen at *m/z* 333.2424 (C_21_H_33_O_3_^+^, 4.8 ppm) and *m/z* 315.2319 (C_21_H_31_O_2_^+^, 14.6 ppm). The tropylium ion at *m/z* 193.1223 (C_12_H_17_O_2_^+^, 4.1 ppm) and the protonated olivetol ion at *m/z* 181.1223 (C_11_H_17_O_2_^+^, 2.8 ppm) can be observed. The ion at *m/z* 153.1274 (C_10_H_17_O^+^, −3.3 ppm) and its anhydrate at *m/z* 135.1168 (C_10_H_15_^+^, −20.7 ppm) are present, indicating two hydroxylation sites on the alicyclic moiety of the molecule. The ion at *m/z* 137.1325 (C_10_H_17_^+^, 2.9 ppm) might result from a coeluting metabolite, as it is indicative of a metabolite that is hydroxylated on the alicyclic moiety and the side chain of the molecule.

Another glucuronidated bishydroxylated metabolite M24 eluted at 5.25 min; the product ion spectrum is found in Figure S28. The [M + H]^+^ ion at *m/z* 527.2851 (C_27_H_43_O_9_^+^, 12.1 ppm) is seen. The deglucuronidated ion at *m/z* 351.2530 (C_21_H_35_O_4_^+^, −0.6 ppm) and the loss of two H_2_O molecules are seen at *m/z* 333.2424 (C_21_H_33_O_3_^+^, 7.8 ppm) and *m/z* 315.2319 (C_21_H_31_O_2_^+^, 4.1 ppm). An ion from the alicyclic moiety can be seen at *m/z* 137.1325 (C_10_H_17_^+^, −8.0 ppm), indicating that this metabolite is hydroxylated on the alicyclic moiety and on the side chain.

Another bishydroxylated glucuronide M25 eluted at 5.67 min; the product ion spectrum is seen in Figure S29. The [M + H]^+^ ion at *m/z* 527.2851 (C_27_H_43_O_9_^+^, −2.8 ppm) and its anhydrate at *m/z* 509.2745 (C_27_H_41_O_8_^+^, −9.4 ppm) are observable. The base peak of the spectrum is the protonated aglycone at *m/z* 351.2530 (C_21_H_35_O_4_^+^, −3.7 ppm). Two losses of H_2_O at *m/z* 333.2424 (C_21_H_33_O_3_^+^, 0.3 ppm) and at *m/z* 315.2319 (C_21_H_31_O_2_^+^, −5.7 ppm) are observed. The hydroxylated olivetol ion at *m/z* 197.1172 (C_11_H_17_O_3_^+^, 0.5 ppm) indicates that this metabolite had a hydroxylation site on the alicyclic moiety and on the side chain. The tropylium ion at *m/z* 193.1223 (C_12_H_17_O_2_^+^, 7.2 ppm) derives from a metabolite that bears two hydroxylation positions on the alicyclic moiety, which leads to the assumption that coelution of metabolites is likely in this case.

### 6.3 GC–MS Phase I Metabolites (TMS Derivatives)

#### 6.3.1 Fragmentation Patterns of H4CBD and Its Trimethylsilylderivatives

The electron impact (EI) mass spectra of (*R*)‐H4CBD and (*S*)‐H4CBD are indistinguishable. They show the molecular ion at *m/z* 318. The main fragments are the dihydrochromenylium ion at *m/z* 233 and the tropylium ion at *m/z* 193. The dihydrochromenylium ion in hydrogenated cannabinoids like H4CBD or HHC is of high abundance. In their unsaturated analogs, CBD and the isomers of THC, the analogous chromenylium ion *m/z* 231 usually forms the base peak and is often the only highly abundant ion due to its simple formation and aromatic stability [24, 25, 26]. The tropylium ion forms the base peak of the H4CBD epimers as it does for the HHC epimers [24, 26, 27, 28].

**FIGURE 6:**
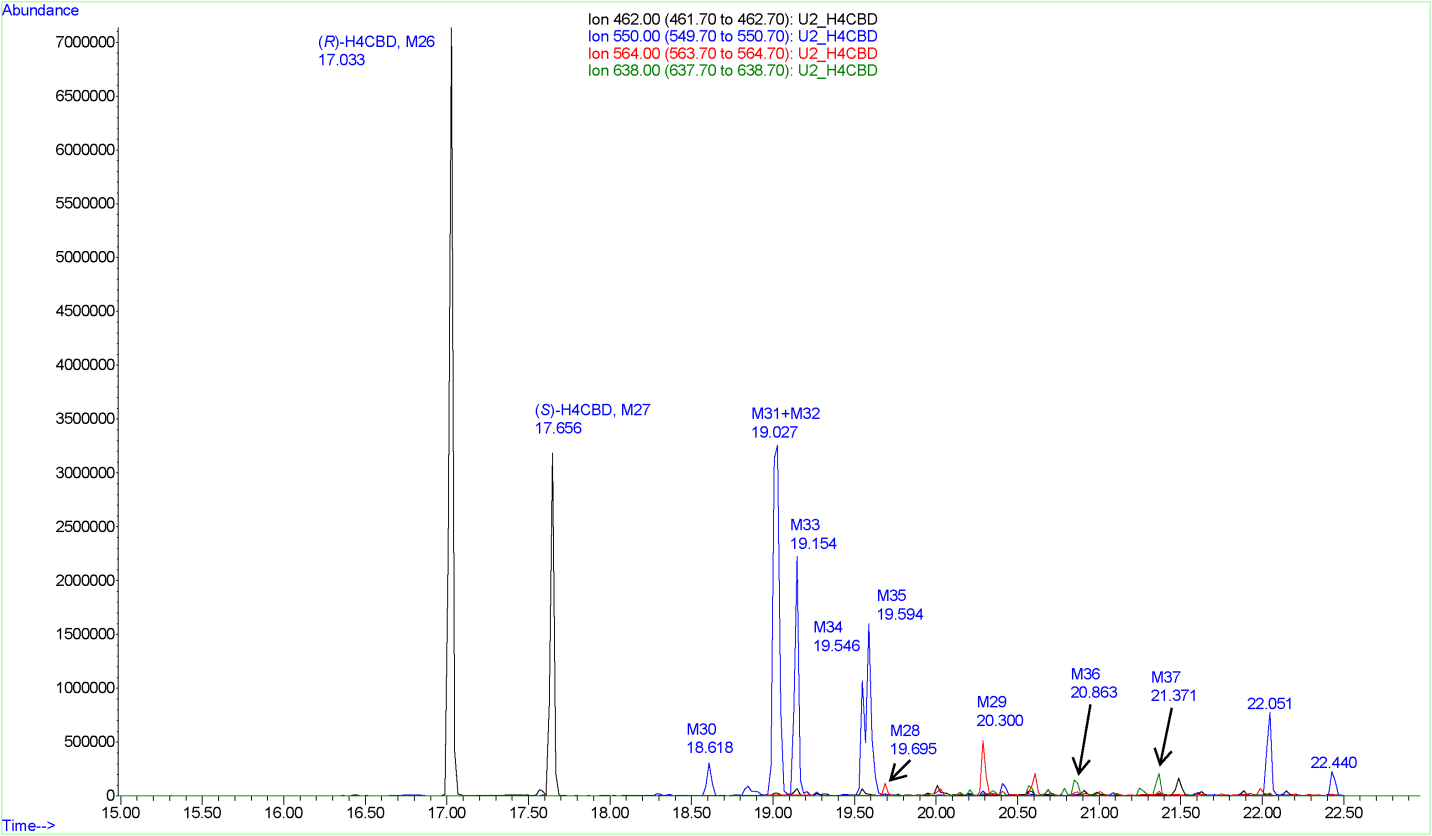
Overlayed XIC of a trimethylsilylated extract from a deglucuronidated urine sample 3 h after oral ingestion of H4CBD. H4CBD (black, M26 and M27), carboxylated metabolites (red, M28 and M29), hydroxylated metabolites (blue, M30–M35), and bishydroxylated metabolites (green, M36 and M37).

The spectra of the trimethylsilylated H4CBD epimers are indistinguishable as well; see Figures S30 and S31. The most common fragments are the trimethylsilylated derivatives of the dihydrochromenylium ion *m/z* 377 and the trimethylsilylated tropylium ion *m/z* 337 discussed previously. The trimethylsilylated metabolites (*R*)‐H4CBD TMS (M26) and (*S*)‐H4CBD (M27) can be seen at 17.03 and 17.66 min, respectively (see Figure 6).

#### 6.3.2 Carboxylated Metabolites, Trimethylsilylated

Two different carboxylated metabolites were detected. The metabolite M28 at 19.70 min shows the molecular ion at *m/z* 564 (see Figure S32). After loss of a trimethylsilyl radical and CO_2_ from the trimethylsilylcarboxy group, the base peak of the spectrum at *m/z* 447 is formed. Additionally, the tropylium ion at *m/z* 337 is present, indicating that this metabolite was carboxylated on the alicyclic moiety. Trimethylsilylated 7‐COOH‐H4CBD fits with the observed fragments. The same fragmentation mechanisms as in 7‐COOH‐CBD occur, with the exception of the retro‐Diels‐Alder fragmentation of the cyclohexene ring due to a lacking double bond in the alicyclic ring [15].

Another carboxylated metabolite M29 is observed at 20.30 min (see Figure S33). This metabolite shows the fragment ion *m/z* 439 and *m/z* 479, which are trimethylsilylcarboxy derivatives of the dihydrochromenylium ion *m/z* 337 and the tropylium ion *m/z* 377, indicating that this metabolite is carboxylated on the side chain. This metabolite was therefore tentatively identified as 5″‐COOH‐H4CBD. The fragment ion *m/z* 439 can also be seen in the mass spectrum of trimethylsilylated 5″‐COOH‐CBD [15].

#### 6.3.3 Hydroxylated Metabolites, Trimethylsilylated

Metabolites that are hydroxylated on the side chain can be recognized by the presence of the ions with *m/z* 425 and *m/z* 465, whereas the ions with *m/z* 337 and *m/z* 377 are absent [29]. The former ions have an additional trimethylsilyloxy group on the side chain.

**FIGURE 7:**
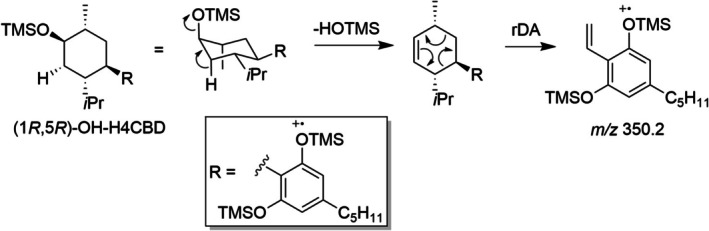
Possible fragmentation pathway to the ion *m/z* 350 seen in the spectrum of (1*R*,5*R*)‐OH‐H4CBD (metabolite M32).

Five different hydroxylated metabolites of H4CBD are seen in the extracted ion chromatogram (XIC *m/z* 550). The minor metabolite M30 eluting at 18.62 min shows the dihydrochromenlyium ion *m/z* 377 as the base peak, indicating that this metabolite is hydroxylated on the alicyclic moiety (see Figure S34). The presence of the ion at *m/z* 392 might be indicative that this metabolite was hydroxylated on C6, synperiplanar to the methyl group at C1. Elimination of trimethylsilanol and subsequent retro‐Diels‐Alder reaction would lead to a fragment ion with *m/z* 392, a similar mechanism as shown in Figure 7. A plausible structure of this metabolite might be (1*R*,6*S*)‐OH‐H4CBD or (1*S*,6*R*)‐OH‐H4CBD, and the latter shows higher 1,3‐diaxial strain in the conformation in which trimethylsilanol could be eliminated due to bulky axial groups. The EI mass spectrum is shown in Figure S34.

At 19.0 min, two different metabolites are almost coeluting; the first metabolite M31 at 19.01 min shows the ions *m/z* 465 and *m/z* 425, indicating that this metabolite is a side‐chain hydroxylated metabolite. The hydroxylation position is unknown; a mass spectrum is shown in Figure S35.

The other metabolite M32 at 19.03 min shows the fragment ions *m/z* 377 and *m/z* 337, indicating a metabolite that is hydroxylated on the alicyclic part (see Figure S36). In addition, this metabolite shows a quite abundant fragment ion with *m/z* 350. This might be indicative that the hydroxylation position is either antiperiplanar to the methyl group or to the *iso*‐propyl group. The tentative metabolite (1*R*,6*R*)‐OH‐H4CBD has a stable chair conformation in which the *iso*‐propyl group and the aromatic moiety are equatorial substituents. In this conformation, the metabolite could eliminate trimethylsilanol, resulting in the formation of a 3,4,6‐trisubstituted cyclohexene intermediate, which can fragment further to a styrene ion with *m/z* 350 after retro‐Diels‐Alder reaction. The potential metabolites (1*S*,5*S*)‐OH‐H4CBD, (1*R*,4*S*)‐OH‐H4CBD, and (1*S*,4*S*)‐OH‐H4CBD might undergo the same reaction cascade, but in these molecules, the conformation in which the elimination of trimethylsilanol could occur would have three bulky axial substituents, resulting in a high 1,3‐diaxial strain. A possible fragmentation pathway is shown in Figure 7.

The mass spectra of the metabolites M33 and M34 eluting at 19.15 and 19.55 min are identical, showing the molecular ion at *m/z* 550 and two abundant ions at *m/z* 447 and *m/z* 337; see Figures S37 and S38. The ion with *m/z* 447 forms the base peak of their spectra and might form after the loss of a trimethylsilyl radical and formaldehyde. A primary alcohol is therefore suspected. The ion *m/z* 337 is the tropylium ion that is also present in the mass spectra of the H4CBD epimers, indicating that these metabolites were hydroxylated on the alicyclic moiety of the molecule. Hydroxylation position on C7 seems plausible; this would explain why the characteristic dihydrochromenylium ion *m/z* 377 is not present. These metabolites were therefore tentatively identified as 7‐OH‐(*R*)‐H4CBD and 7‐OH‐(*S*)‐H4CBD. The same fragmentation reactions occur in the TMS derivative of the analogue 7‐OH‐CBD, where the ion *m/z* 443 (unsaturated analogue of *m/z* 447) forms after the loss of a trimethylsilyl radical and formaldehyde; the ion *m/z* 337 is also present. An additional ion is formed in 7‐OH‐CBD with *m/z* 478, which cannot be formed from 7‐OH‐H4CBD, as this ion occurs from a retro‐Diels‐Alder pathway [14].

The metabolite M35 eluting at 19.59 min shows the ions *m/z* 465 and *m/z* 425 (see Figure S39). These are the discussed dihydrochromenylium ion (*m/z* 377) and tropylium ion (*m/z* 337) with an additional trimethylsilyloxy group, indicating that this metabolite is hydroxylated on the side chain.

#### 6.3.4 Bishydroxylated Metabolites, Trimethylsilylated

The metabolite M36 at 20.86 min shows the ion *m/z* 535, which is indicative of a hydroxylation position on C7 after the loss of a trimethylsilyl radical and formaldehyde (see Figure S40). The fragment ions at *m/z* 465 and *m/z* 425 are present, indicating a hydroxylation position on the side chain.

The metabolite M37 at 21.37 min shows two very abundant fragment ions (see Figure S41). The ion *m/z* 535 can be formed after the elimination of a trimethylsilyl radical and formaldehyde, the same fragmentation mechanism as in the tentatively identified 7‐OH‐H4CBD epimers and the metabolite M36. The other ion (*m/z* 425) is indicative of a hydroxylation position at the side chain. This metabolite was therefore tentatively identified as a side‐chain hydroxylated derivative of 7‐OH‐H4CBD; an EI mass spectrum is shown in Figure S41. The analogous fragment ion *m/z* 531 and the same fragment ion *m/z* 425 are observed for the bishydroxylated metabolites of CBD 1″,7‐DiOH‐CBD, 3″,7‐DiOH‐CBD, 4″,7‐DiOH‐CBD, and 5″,7‐DiOH‐CBD [14, 15].

The Kováts indices, retention times, and relevant ions of the trimethylsilyl derivatives of H4CBD and the detected metabolites are summarized in Table 3. A chromatogram of the *n*‐alkane standard that was used for the calculation of the Kováts indices can be found in Figure S46.

### 6.4 Fragmentation Patterns of Phase I Metabolites

In ESI+, the fragmentation patterns of H4CBD are different from CBD. H4CBD does not fragment to the characteristic tropylium ion *m/z* 193.1223; instead, the aromatic moiety of the molecule can be seen as a protonated olivetol at *m/z* 181.1223 after fragmentation under ESI+ conditions. This is also true for their respective metabolites, side‐chain hydroxylated metabolites of H4CBD fragment to a hydroxy‐olivetol ion *m/z* 197.1178 and its anhydrate *m/z* 179.1065. H4CBD metabolites that are hydroxylated on the alicyclic moiety can undergo fragmentation to the characteristic tropylium ion *m/z* 193.1223. The presence of these ions in H4CBD metabolites is therefore indicative of the hydroxylation position.

Very unusual fragmentation patterns are found for several Phase I and Phase II metabolites. A Phase I metabolite (M2) that was tentatively identified as a carboxylic acid showed the fragment ion *m/z* 227.1794 that was identified as (C_17_H_23_^+^). It is not clear how this ion is formed. A similar ion (*m/z* 225.1) is found in the mass spectrum of 7‐COOH‐CBD under ESI+ conditions.

The side‐chain hydroxylated metabolites M4 and M5 showed loss of both oxygens from the phenol groups, presumably as H_2_O and CO to form a cyclopentadienyl cation. The same fragmentation pattern to form a cyclopentadienyl ion can be seen in the ESI+ mass spectra of hydroquinone, pyrocatechol, and vanillin [30, 31].

The side‐chain hydroxylated metabolites M6 and M7 form another tropylium ion (*m/z* 261.1849), which results from the fragmentation of the side chain, in contrast to the formation of the tropylium ion *m/z* 193.1223, which is formed from the fragmentation of the alicyclic moiety.

The fragmentation patterns of the trimethylsilylated derivatives of H4CBD and its Phase I metabolites under EI ionization are similar to those of the trimethylsilylated derivatives of CBD and its Phase I metabolites.

### 6.5 Fragmentation Patterns of Phase II Metabolites

Several Phase II metabolites showed odd‐electron fragment ions, which are presumably formed by the fragmentation of the glucuronide moiety to form a furanyl radical cation. This kind of fragmentation was seen for the glucuronides of H4CBD (M11, M12), the side‐chain carboxylated metabolite 5″‐COOH‐H4CBD (M13), and the side‐chain hydroxylated metabolite M15. The Phase II metabolites with functional groups on the alicyclic moiety did not show this fragmentation pattern. It is therefore assumed that side‐chain oxidized metabolites of H4CBD can easily be protonated on the glucuronide, which is prone to fragmentation.

The tentatively identified metabolites 7‐OH‐H4CBD glucuronide and 7‐COOH‐H4CBD glucuronide (M14) showed a formal loss of C_2_H_12_O_2_ (two H_2_O and two CH_4_) by an unknown process. The glucuronide moiety seems not to be fragmented in this fragmentation pathway because, in the case of the carboxy metabolite M14, the deglucuronidated species was found as well.

## 7 Conclusions

The epimers of H4CBD and their respective glucuronides can be used as analytical targets, but might not be present because of metabolization, depending on the time between consumption and urine sampling. In this case, the side‐chain hydroxylated metabolites M4 and M5, and the metabolite M8, which is presumably an epimer of 7‐OH‐H4CBD, might be better targets as they were very abundant in the chromatogram. Further investigations are needed to elucidate the structures of the very abundant side‐chain hydroxylated metabolites M4 and M5. A method for the determination of H4CBD and its metabolites should also include the carboxylic acids 7‐COOH‐H4CBD and 5″‐COOH‐H4CBD, and it is assumed that they accumulate after frequent H4CBD consumption. The same metabolites can be analyzed as TMS derivatives by GC–MS. Glucuronides of the discussed metabolites would also suit as forensic markers. Currently, no analytical standards for the proof of H4CBD consumption are available besides (*R*)‐H4CBD and (*S*)‐H4CBD.

## Conflicts of Interest

The authors declare no conflicts of interest.

## Supporting information


**Figure S1:** Quantification of (*R*)‐ and (*S*)‐H4CBD in the H4CBD product for recreational use with GC–MS.
**Figure S2:** Chromatogram of deglucuronidated urine before ingestion of H4CBD, measured on a LC‐QqTOF.
**Figure S3:** LC‐QqTOF spectrum of (*S*)‐H4CBD (Metabolite M1).
**Figure S4:** LC‐QqTOF spectrum of (*R*)‐H4CBD (Metabolite M1).
**Figure S5:** Mass spectrum of metabolite M2, a carboxylated metabolite of H4CBD (from a deglucuronidated urine sample 3 h after ingestion of 25‐mg H4CBD).
**Figure S6:** Mass spectrum of metabolite M3, a carboxylated metabolite of H4CBD (from a deglucuronidated urine sample 3 h after ingestion of 25‐mg H4CBD).
**Figure S7:** Mass spectrum of metabolite M4, a side‐chain hydroxylated metabolite of H4CBD (from a deglucuronidated urine sample 3 h after ingestion of 25‐mg H4CBD). *The position of the hydroxy group is unknown.
**Figure S8:** Mass spectrum of metabolite M5, a side‐chain hydroxylated metabolite of H4CBD (from a deglucuronidated urine sample 3 h after ingestion of 25‐mg H4CBD)*.* *The position of the hydroxy group is unknown.
**Figure S9:** Mass spectrum of metabolite M6, a hydroxylated metabolite of H4CBD (from a deglucuronidated urine sample 3 h after ingestion of 25‐mg H4CBD). Tentatively identified as an epimer of 2″OH‐H4CBD.
**Figure S10:** Mass spectrum of metabolite M7, a hydroxylated metabolite of H4CBD (from a deglucuronidated urine sample 3 h after ingestion of 25‐mg H4CBD). Tentatively identified as an epimer of 2″OH‐H4CBD.
**Figure S11:** Mass spectrum of metabolite M8, a hydroxylated metabolite of H4CBD (from a deglucuronidated urine sample 3 h after ingestion of 25‐mg H4CBD). Tentatively identified as an epimer of 7‐OH‐H4CBD.
**Figure S12:** Mass spectrum of metabolite M9, a hydroxylated metabolite of H4CBD (from a deglucuronidated urine sample 3 h after ingestion of 25‐mg H4CBD). Tentatively identified as an epimer of 7‐OH‐H4CBD.
**Figure S13:** Mass spectrum of metabolite M10, a bishydroxylated metabolite of H4CBD (from a deglucuronidated urine sample 3 h after ingestion of 25‐mg H4CBD). The position of the hydroxy groups is unknown.
**Figure S14:** Chromatogram of a urine sample before ingestion of H4CBD, measured on a LC‐QqTOF.
**Figure S15:** Mass spectrum of metabolite M11, a glucuronidated metabolite of H4CBD (from a urine sample 3 h after ingestion of 25‐mg H4CBD).
**Figure S16:** Mass spectrum of metabolite M12, a glucuronidated metabolite of H4CBD (from a urine sample 3 h after ingestion of 25‐mg H4CBD). Acquired in SWATH mode.
**Figure S17:** Mass spectrum of metabolite M13, a carboxylated and glucuronidated metabolite of H4CBD (from a urine sample 3 h after ingestion of 25‐mg H4CBD). The position of the glucuronide moiety is unknown.
**Figure S18:** Mass spectrum of metabolite M14, a carboxylated and glucuronidated metabolite of H4CBD (from a urine sample 3 h after ingestion of 25‐mg H4CBD). The position of the glucuronide moiety is unknown.
**Figure S19:** Mass spectrum of metabolite M15, a hydroxylated and glucuronidated metabolite of H4CBD (from a urine sample 3 h after ingestion of 25‐mg H4CBD). The position of the glucuronide moiety is unknown.
**Figure S20:** Mass spectrum of metabolite M16, a hydroxylated and glucuronidated metabolite of H4CBD (from a urine sample 3 h after ingestion of 25‐mg H4CBD). The position of the glucuronide moiety and the hydroxy group is unknown.
**Figure S21:** Mass spectrum of metabolite M17, a hydroxylated and glucuronidated metabolite of H4CBD (from a urine sample 3 h after ingestion of 25‐mg H4CBD). The position of the glucuronide moiety and the hydroxy group is unknown.
**Figure S22:** Mass spectrum of metabolite M18, a hydroxylated and glucuronidated metabolite of H4CBD (from a urine sample 3 h after ingestion of 25‐mg H4CBD). The position of the glucuronide moiety is unknown.
**Figure S23:** Mass spectrum of metabolite M19, a hydroxylated and glucuronidated metabolite of H4CBD (from a urine sample 3 h after ingestion of 25‐mg H4CBD). The position of the glucuronide moiety is unknown.
**Figure S24:** Mass spectrum of metabolite M20, a hydroxylated and glucuronidated metabolite of H4CBD (from a urine sample 3 h after ingestion of 25‐mg H4CBD). The position of the hydroxy group and the glucuronide is unknown.
**Figure S25:** Mass spectrum of metabolite M21, a hydroxylated and glucuronidated metabolite of H4CBD (from a urine sample 3 h after ingestion of 25‐mg H4CBD). The position of the hydroxy group and the glucuronide is unknown.
**Figure S26:** Mass spectrum of metabolite M22, a hydroxylated and glucuronidated metabolite of H4CBD (from a urine sample 3 h after ingestion of 25‐mg H4CBD). The position of the hydroxy group and the glucuronide is unknown.
**Figure S27:** Mass spectrum of metabolite M23, a bishydroxylated and glucuronidated metabolite of H4CBD (from a urine sample 3 h after ingestion of 25‐mg H4CBD). Both hydroxylation positions are found on the alicyclic moiety, and their position and the position of the glucuronide are not clear.
**Figure S28:** Mass spectrum of metabolite M24, a bishydroxylated and glucuronidated metabolite of H4CBD (from a urine sample 3 h after ingestion of 25‐mg H4CBD). One hydroxylation position is found on the alicyclic moiety, the other hydroxylation position is on the side‐chain, and their position and the position of the glucuronide are not clear.
**Figure S29:** Mass spectrum of metabolite M25, a bishydroxylated and glucuronidated metabolite of H4CBD (from a urine sample 3 h after ingestion of 25‐mg H4CBD). Both hydroxylation positions are found on the alicyclic moiety, and their position and the position of the glucuronide are not clear. The ion *m/z* 197.1173 could result from a coeluting metabolite with a hydroxylation position on the side‐chain.
**Figure S30:** EI mass spectrum of the metabolite M26 (*R*)‐H4CBD TMS.
**Figure S31:** EI mass spectrum of the metabolite M27 (*S*)‐H4CBD TMS, the same ions are formed as for (*R*)‐H4CBD TMS.
**Figure S32:** EI mass spectrum of the carboxylated metabolite M28 7‐COOH‐H4CBD TMS.
**Figure S33:** EI mass spectrum of the carboxylated metabolite M29 5″‐COOH‐H4CBD TMS.
**Figure S34:** EI mass spectrum of the hydroxylated metabolite M30, hydroxylated on the alicyclic moiety.
**Figure S35:** EI mass spectrum of the hydroxylated metabolite M31, hydroxylated on the side‐chain. Hydroxylation position unknown.
**Figure S36:** EI mass spectrum of the hydroxylated metabolite M32, hydroxylated on the alicyclic moiety.
**Figure S37:** EI mass spectrum of the hydroxylated metabolite M33, hydroxylated on the alicyclic moiety. Presumably 7‐OH‐(*R*)‐H4CBD.
**Figure S38:** EI mass spectrum of the hydroxylated metabolite M34, hydroxylated on the alicyclic moiety. Presumably 7‐OH‐(*S*)‐H4CBD.
**Figure S39:** EI mass spectrum of the hydroxylated metabolite M35, hydroxylated on the side‐chain. Hydroxylation position unknown.
**Figure S40:** EI mass spectrum of the bishydroxylated metabolite M36, hydroxylated on C7 of the alicyclic moiety and on the side‐chain. Position of the side‐chain hydroxylation is unknown.
**Figure S41:** EI mass spectrum of the bishydroxylated metabolite M37, hydroxylated on C7 of the alicyclic moiety and on the side‐chain. Position of the side‐chain hydroxylation is unknown.
**Figure S42:** Product ion spectrum of 7‐COOH‐CBD at a collision energy of +46 V.
**Figure S43:** Product ion spectrum of 7‐COOH‐CBD at a collision energy of +73 V.
**Figure S44:** Product ion spectrum of (*S*)‐H4CBD (reference standard).
**Figure S45:** Product ion spectrum of (*R*)‐H4CBD (reference standard).
**Figure S46:** Chromatogram of an *n*‐alkane standard (C7–C40) used for the determination of Kováts indices.

## Data Availability

The data that support the findings of this study are available from the corresponding author upon reasonable request.
