# Supplementary material for: Identification of Tetrahydrocannabidiol Metabolites in Human Urine
Source: Drug Test Anal. 2025 Sep 2;17(12):2333–46. doi: 10.1002/dta.3945 (PMC12689245; doi:10.1002/dta.3945)
Supplement: Supplementary file 1 — Figure S1: Quantification of (R)‐ and (S)‐H4CBD in the H4CBD product for recreational use with GC–MS. Figure S2: Chromatogram of deglucuronidated urine before ingestion of H4CBD, measured on a LC‐QqTOF. Figure S3: LC‐QqTOF spectrum of (S)‐H4CBD (Metabolite M1). Figure S4: LC‐QqTOF spectrum of (R)‐H4CBD (Metabolite M1). Figure S5: Mass spectrum of metabolite M2, a carboxylated metabolite of H4CBD (from a deglucuronidated urine sample 3 h after ingestion of 25‐mg H4CBD). Figure S6: Mass spectrum of metabolite M3, a carboxylated metabolite of H4CBD (from a deglucuronidated urine sample 3 h after ingestion of 25‐mg H4CBD). Figure S7: Mass spectrum of metabolite M4, a side‐chain hydroxylated metabolite of H4CBD (from a deglucuronidated urine sample 3 h after ingestion of 25‐mg H4CBD). *The position of the hydroxy group is unknown. Figure S8: Mass spectrum of metabolite M5, a side‐chain hydroxylated metabolite of H4CBD (from a deglucuronidated urine sample 3 h after ingestion of 25‐mg H4CBD). *The position of the hydroxy group is unknown. Figure S9: Mass spectrum of metabolite M6, a hydroxylated metabolite of H4CBD (from a deglucuronidated urine sample 3 h after ingestion of 25‐mg H4CBD). Tentatively identified as an epimer of 2″OH‐H4CBD. Figure S10: Mass spectrum of metabolite M7, a hydroxylated metabolite of H4CBD (from a deglucuronidated urine sample 3 h after ingestion of 25‐mg H4CBD). Tentatively identified as an epimer of 2″OH‐H4CBD. Figure S11: Mass spectrum of metabolite M8, a hydroxylated metabolite of H4CBD (from a deglucuronidated urine sample 3 h after ingestion of 25‐mg H4CBD). Tentatively identified as an epimer of 7‐OH‐H4CBD. Figure S12: Mass spectrum of metabolite M9, a hydroxylated metabolite of H4CBD (from a deglucuronidated urine sample 3 h after ingestion of 25‐mg H4CBD). Tentatively identified as an epimer of 7‐OH‐H4CBD. Figure S13: Mass spectrum of metabolite M10, a bishydroxylated metabolite of H4CBD (from a deglucuronidated urine sample 3 h after i [file DTA-17-2333-s001.docx]

Supplementary Information

Identification of of tetrahydrocannabidiol metabolites in human urine

**Willi Schirmer^1, 2^, Isabelle Mösch^1^, Stefan Schürch^2^, Wolfgang Weinmann^1^**

^1^Institute of Forensic Medicine, Forensic Toxicology and Chemistry, University of Bern, Murtenstrasse 26, 3008 Bern, Switzerland

^2^Department of Chemistry, Biochemistry and Pharmaceutical Sciences, University of Bern, Freiestrasse 3, 3012 Bern, Switzerland

Corresponding author:

Willi Schirmer, Institute of Forensic Medicine, Forensic Toxicology and Chemistry,
University of Bern, Murtenstrasse 26, 3008 Bern, Switzerland

e-mail address: [willi.schirmer@irm.unibe.ch](mailto:willi.schirmer@irm.unibe.ch)

**Table of contents (Figures S1-S46)**

- Quantification of H4CBD product by GC-MS **S1**
- Chromatogram of a deglucuronidated blank urine sample **S2**
- LC-HRMS/MS spectra of deglucuronidated H4CBD metabolites **S3-S13**
  - Metabolites M1 – M10 (Phase I metabolites)
- Chromatogram of a blank urine sample **S14**
- LC-HRMS/MS spectra of deglucuronidated H4CBD metabolites **S15-S29**
  - Metabolites M11 – M25 (Phase II metabolites)
- GC-MS spectra of deglucuronidated H4CBD metabolites **S30-S41**
  - Metabolites M26 – M37 (Phase I metabolites, TMS derivatives)
- LC-HRMS/MS spectra of 7-COOH-CBD **S42-S43**
- LC-HRMS/MS spectra of (*S*)- and (*R*)-H4CBD **S44-S45**
- Chromatogram of an *n*-alkane standard (Kováts indices) **S46**


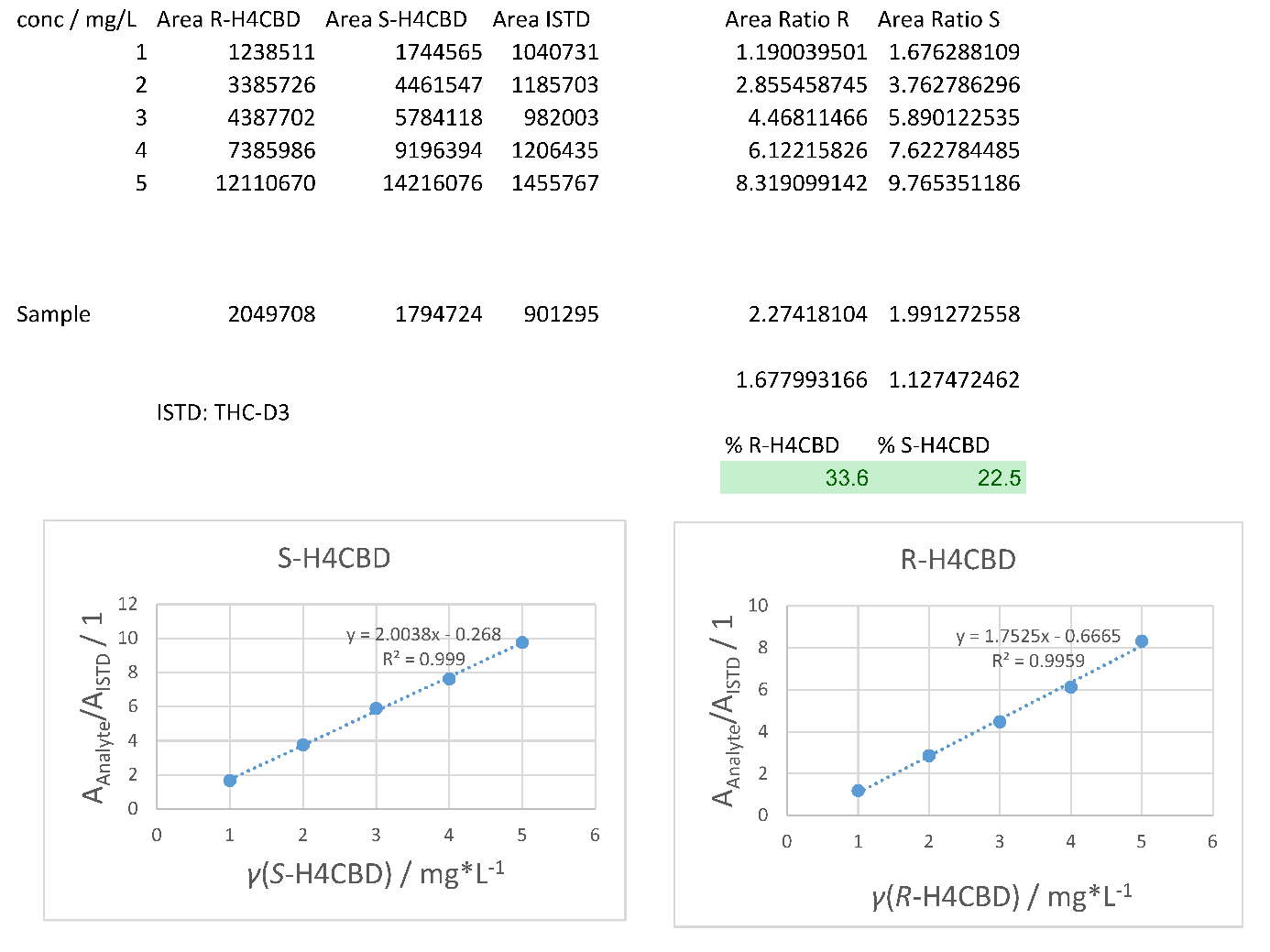


Figure S1: Quantification of (R)- and (S)-H4CBD in the H4CBD product for recreational use with GC-MS.


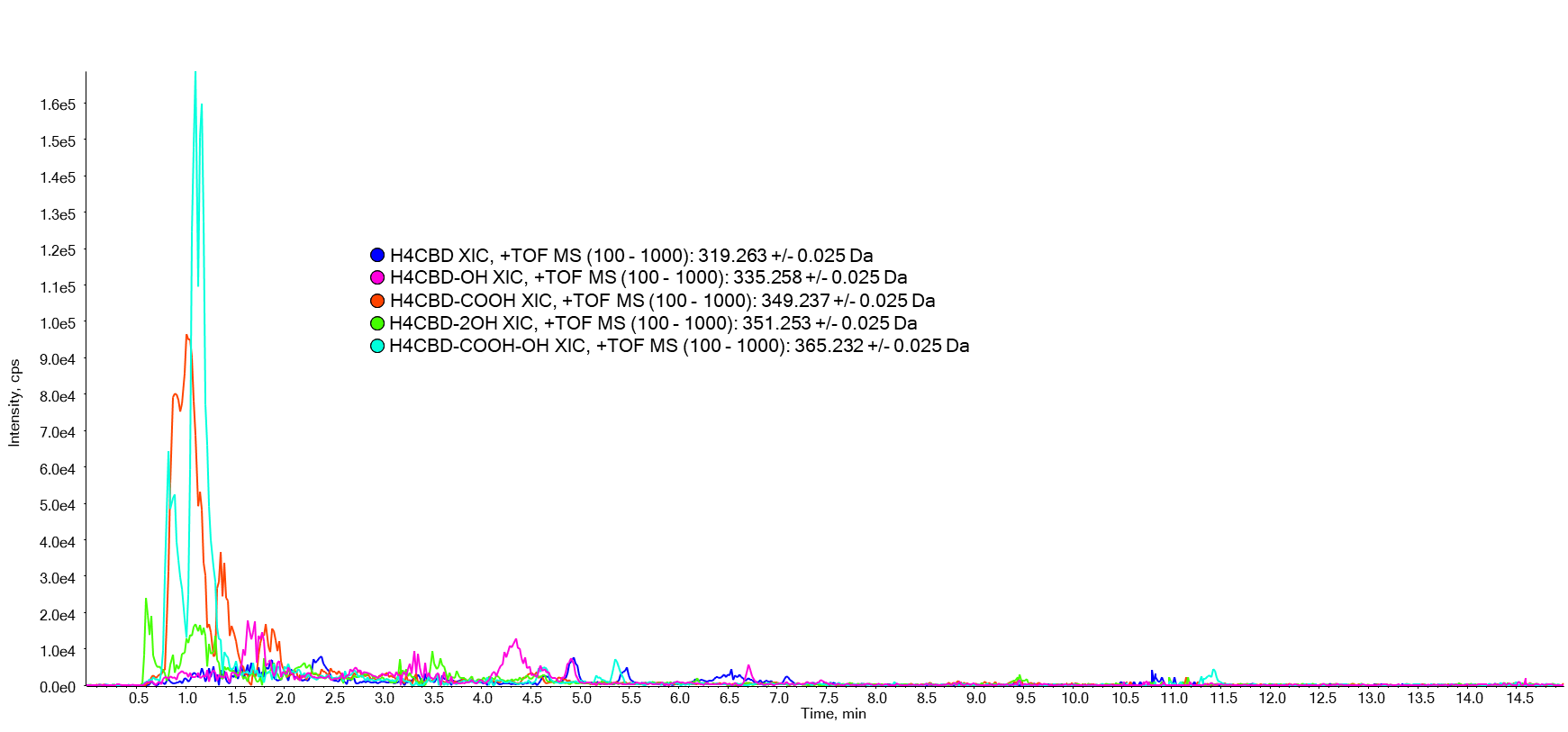


Figure S2: Chromatogram of deglucuronidated urine before ingestion of H4CBD, measured on a LC-QqTOF

*
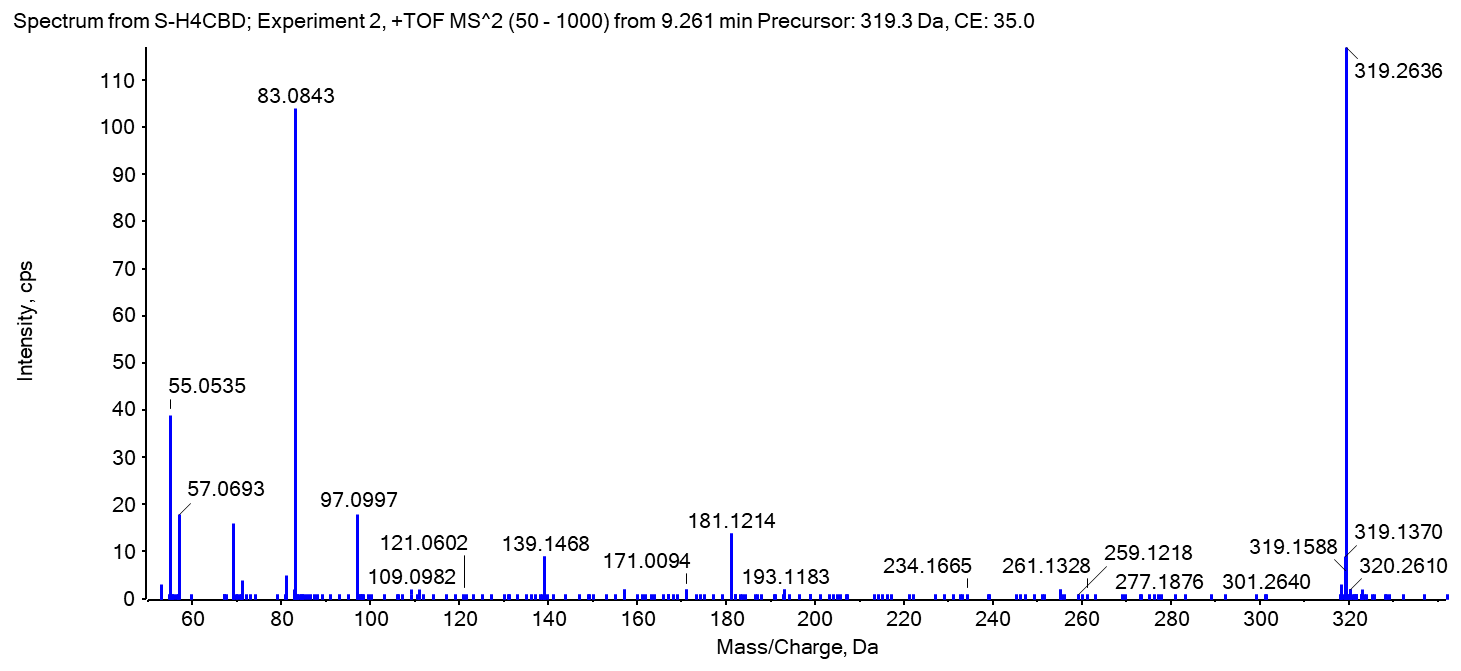
*

Figure S3: LC-QqTOF spectrum of (S)-H4CBD (Metabolite M1)


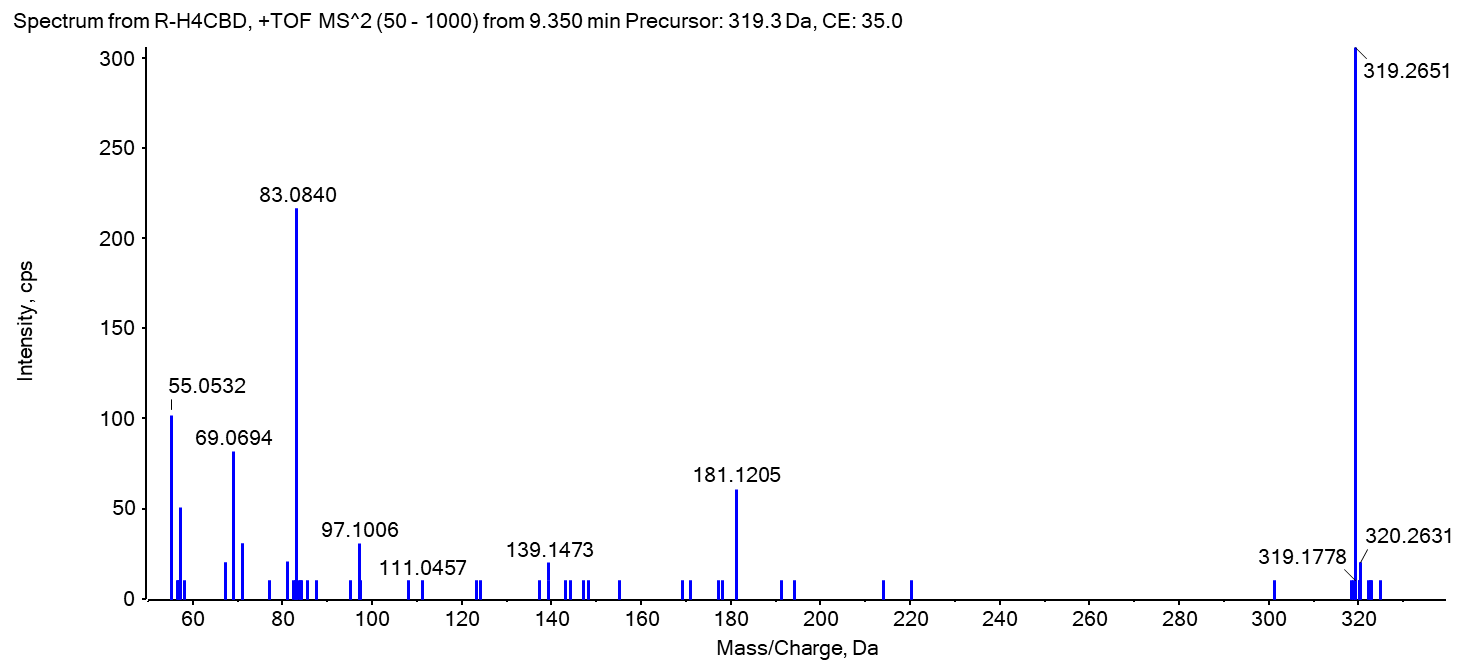


Figure S4: LC-QqTOF spectrum of (R)-H4CBD (Metabolite M1)


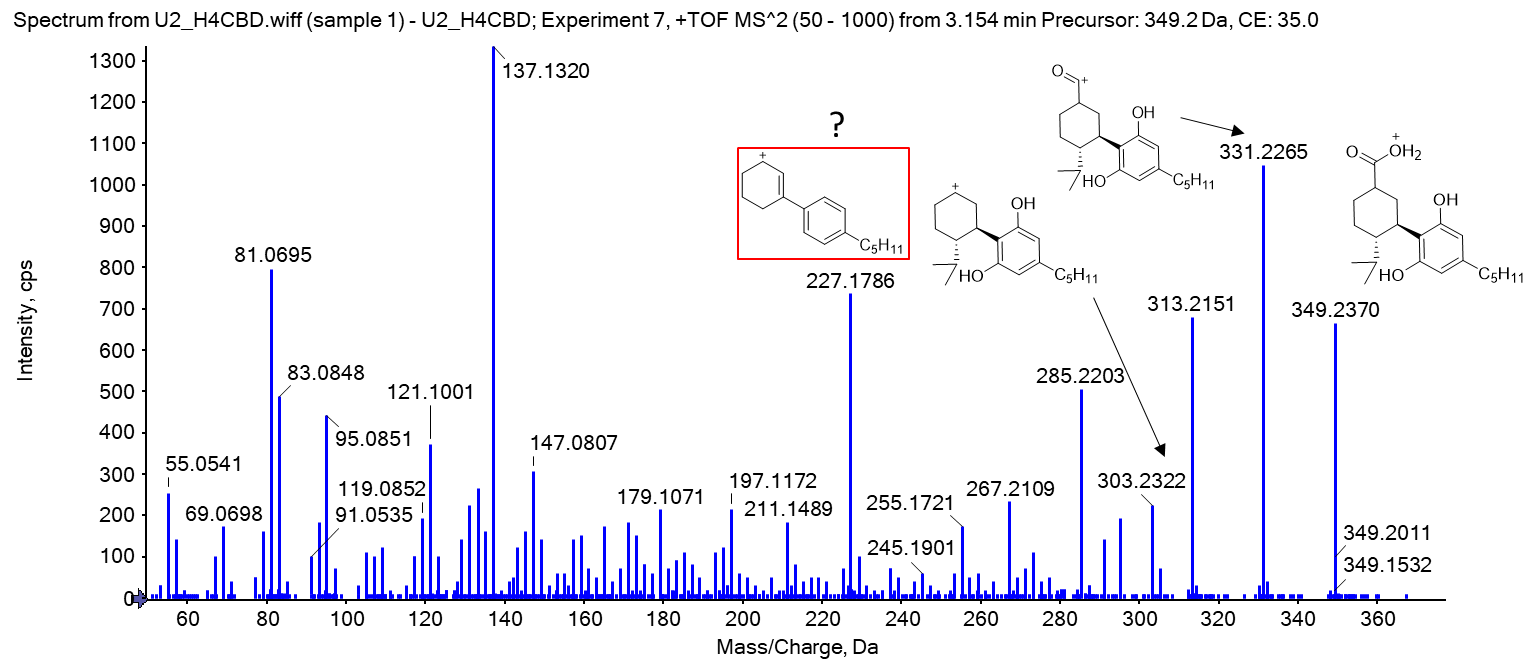


Figure S5: Mass spectrum of metabolite M2, a carboxylated metabolite of H4CBD (from a deglucuronidated urine sample 3 h after ingestion of 25 mg H4CBD)


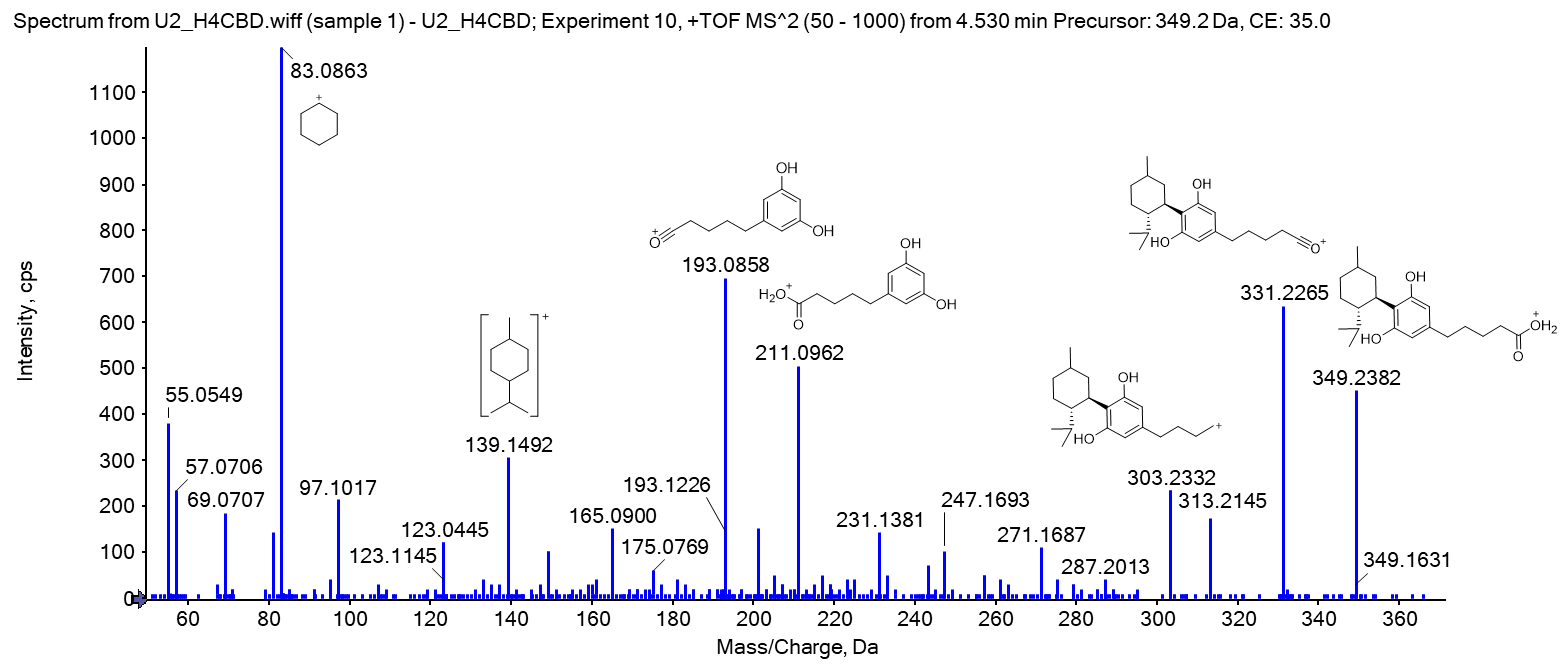


Figure S6: Mass spectrum of metabolite M3, a carboxylated metabolite of H4CBD (from a deglucuronidated urine sample 3 h after ingestion of 25 mg H4CBD)


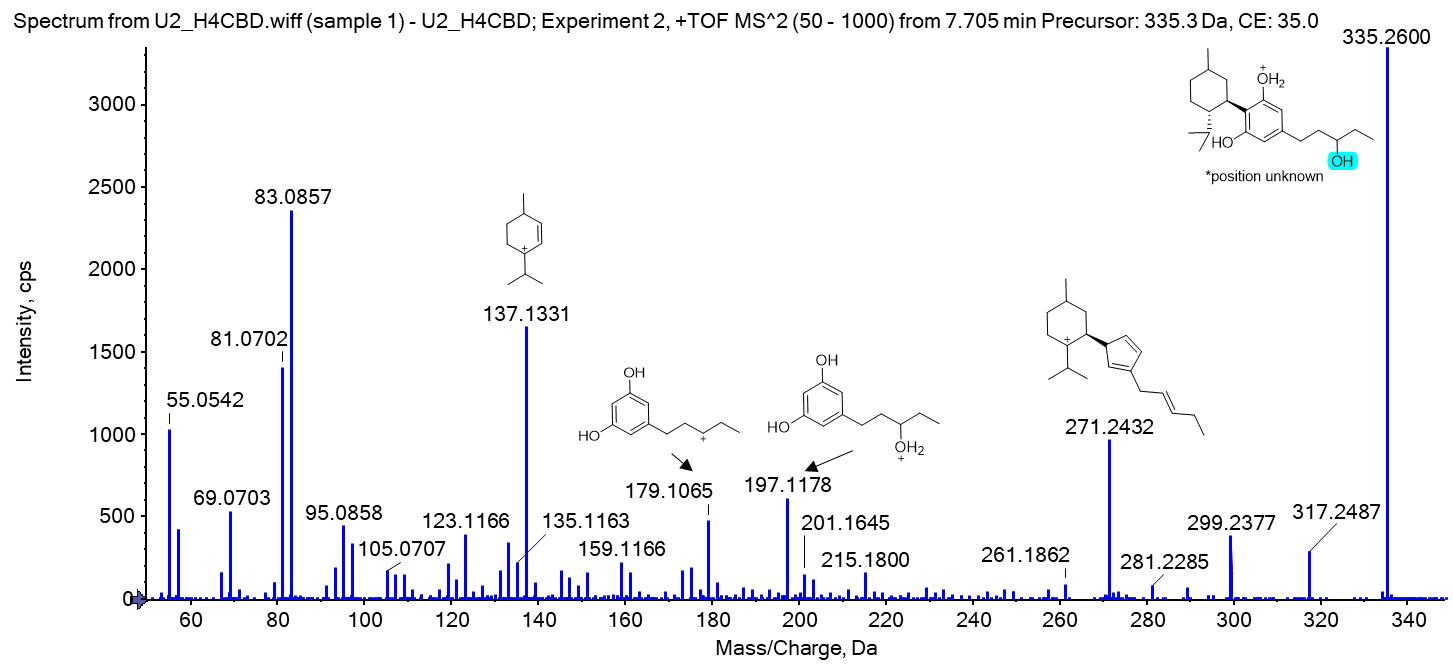


Figure S7: Mass spectrum of metabolite M4, a side chain hydroxylated metabolite of H4CBD (from a deglucuronidated urine sample 3 h after ingestion of 25 mg H4CBD). *The position of the hydroxy group is unknown.


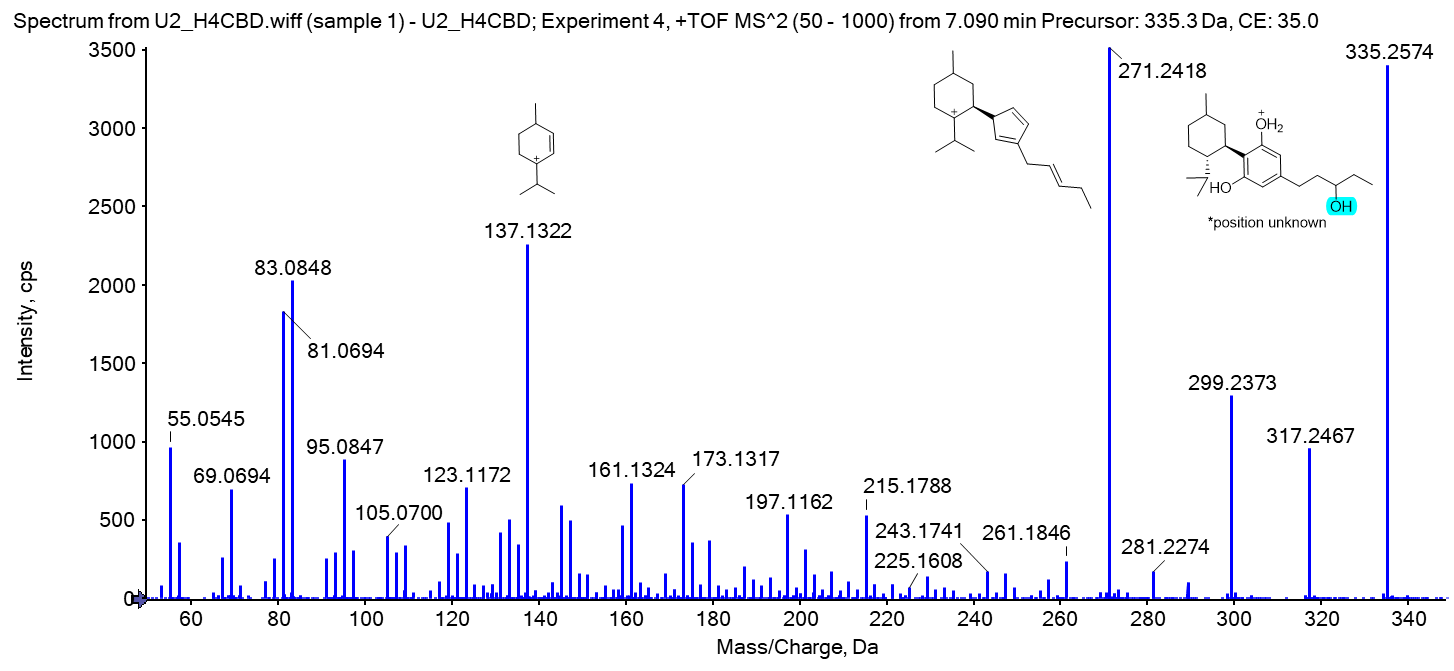


Figure S8: Mass spectrum of metabolite M5, a side chain hydroxylated metabolite of H4CBD (from a deglucuronidated urine sample 3 h after ingestion of 25 mg H4CBD). *The position of the hydroxy group is unknown.
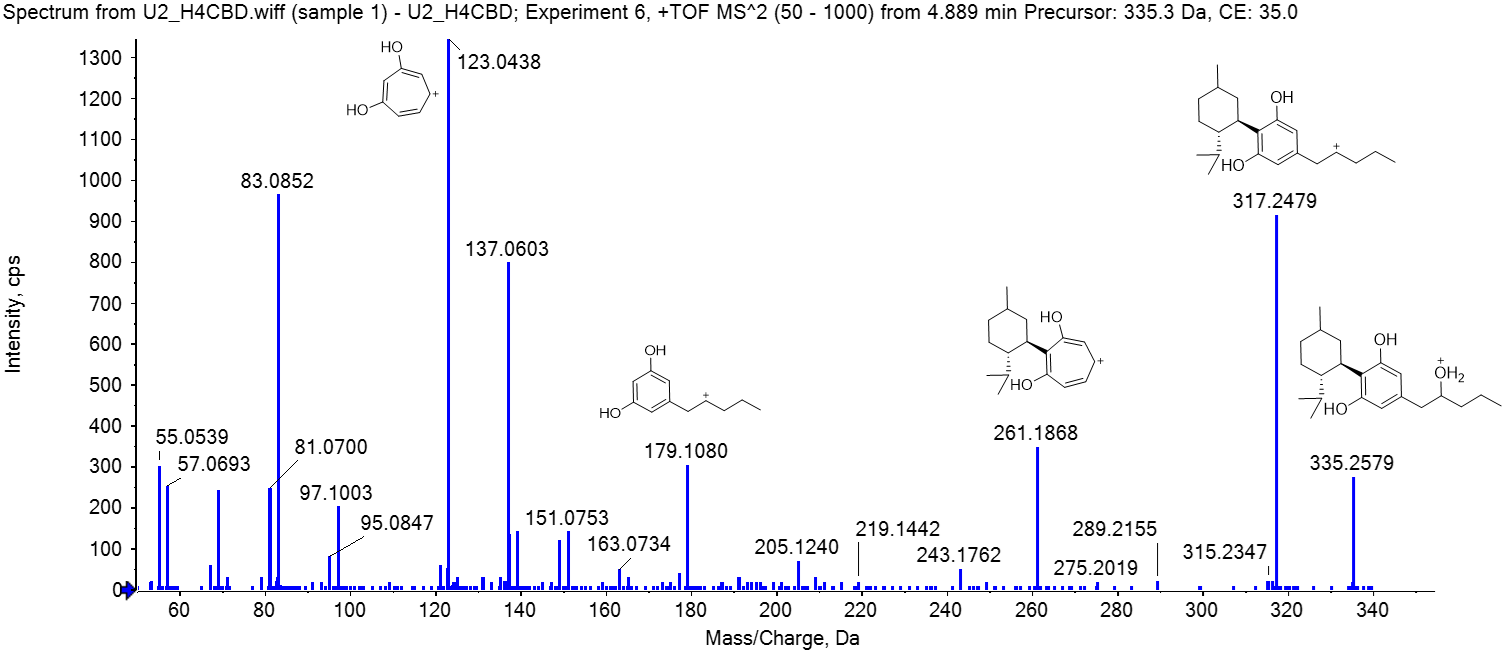


Figure S9: Mass spectrum of metabolite M6, a hydroxylated metabolite of H4CBD (from a deglucuronidated urine sample 3 h after ingestion of 25 mg H4CBD). Tentatively identified as an epimer of 2''OH-H4CBD.

*
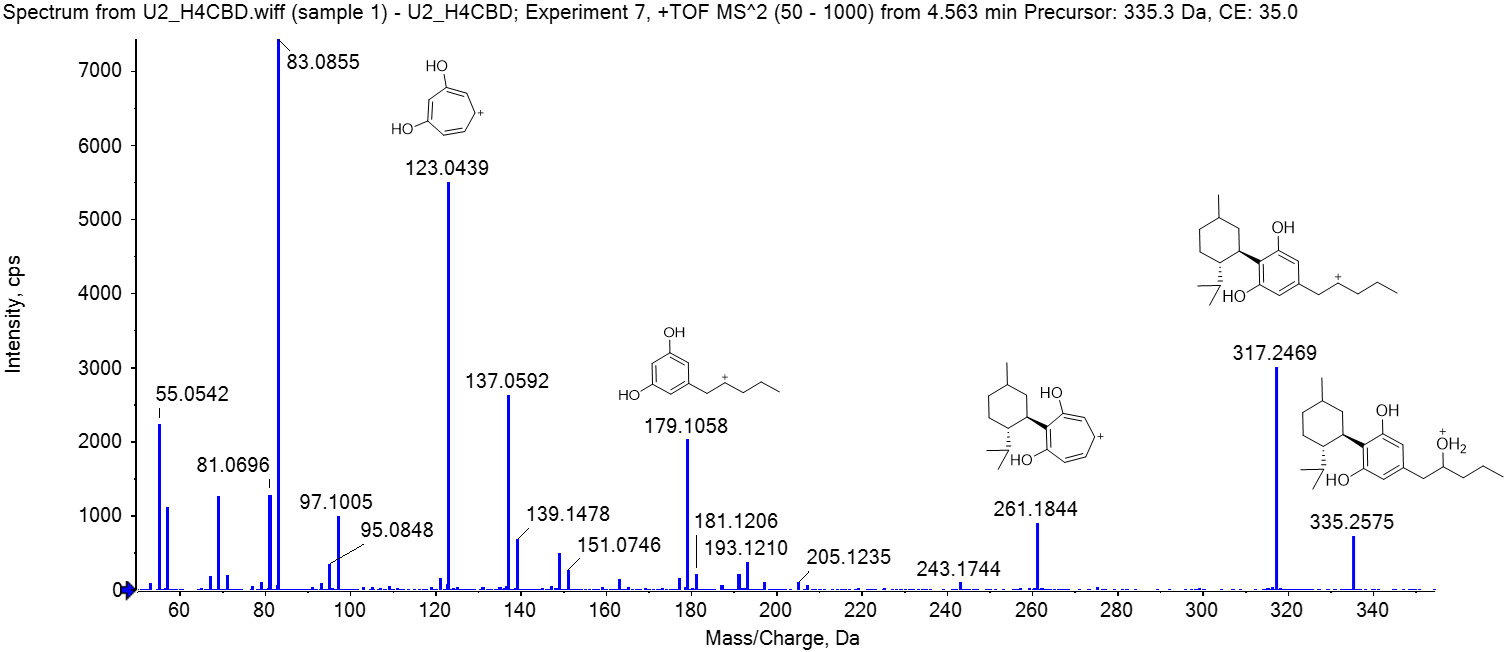
*

Figure S10: Mass spectrum of metabolite M7, a hydroxylated metabolite of H4CBD (from a deglucuronidated urine sample 3 h after ingestion of 25 mg H4CBD). Tentatively identified as an epimer of 2''OH-H4CBD.


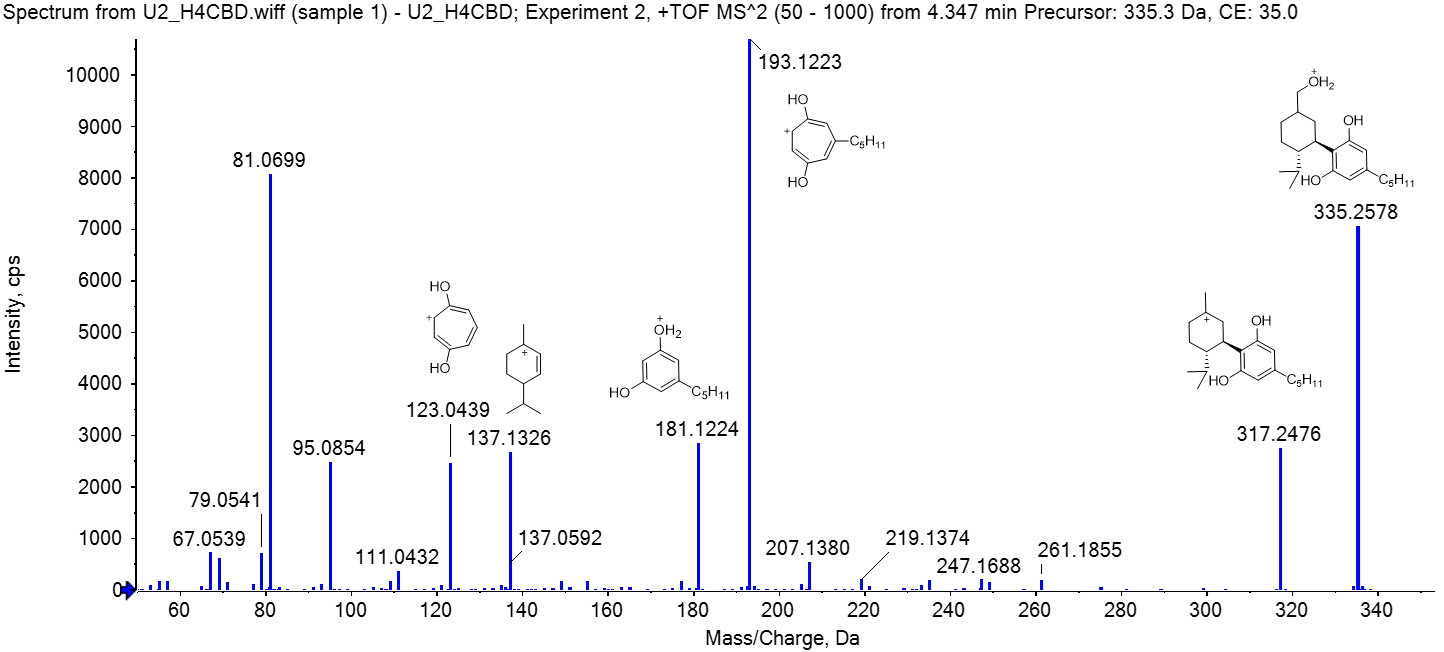


Figure S11: Mass spectrum of metabolite M8, a hydroxylated metabolite of H4CBD (from a deglucuronidated urine sample 3 h after ingestion of 25 mg H4CBD). Tentatively identified as an epimer of 7-OH-H4CBD.

*
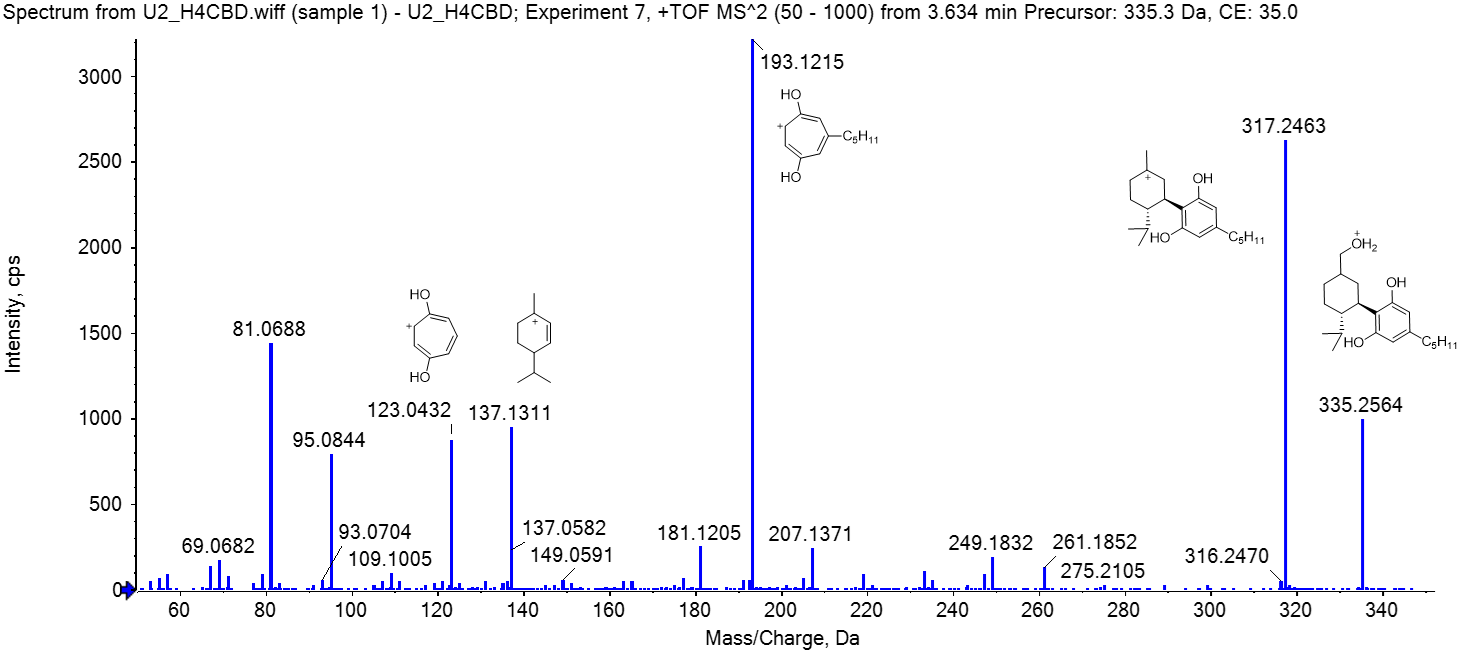
*

Figure S12: Mass spectrum of metabolite M9, a hydroxylated metabolite of H4CBD (from a deglucuronidated urine sample 3 h after ingestion of 25 mg H4CBD). Tentatively identified as an epimer of 7-OH-H4CBD.


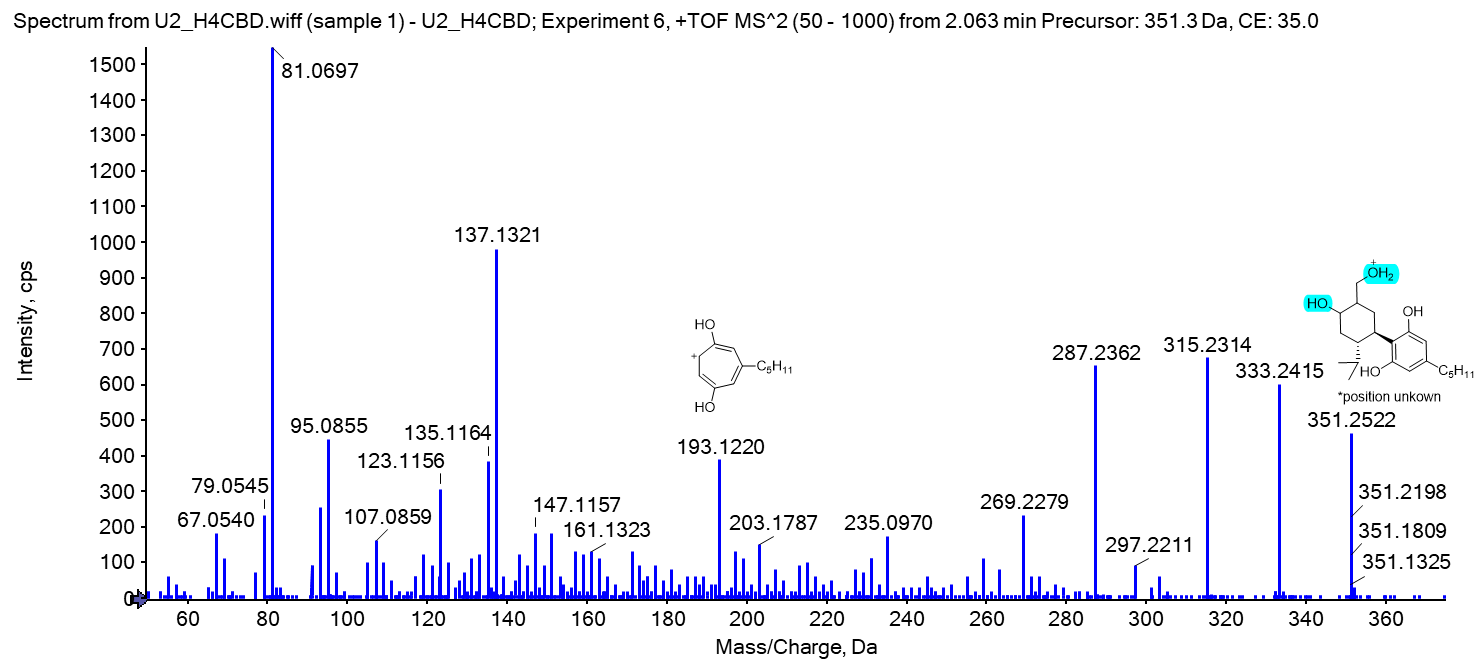


Figure S13: Mass spectrum of metabolite M10, a bishydroxylated metabolite of H4CBD (from a deglucuronidated urine sample 3 h after ingestion of 25 mg H4CBD). The position of the hydroxy groups are unknown.


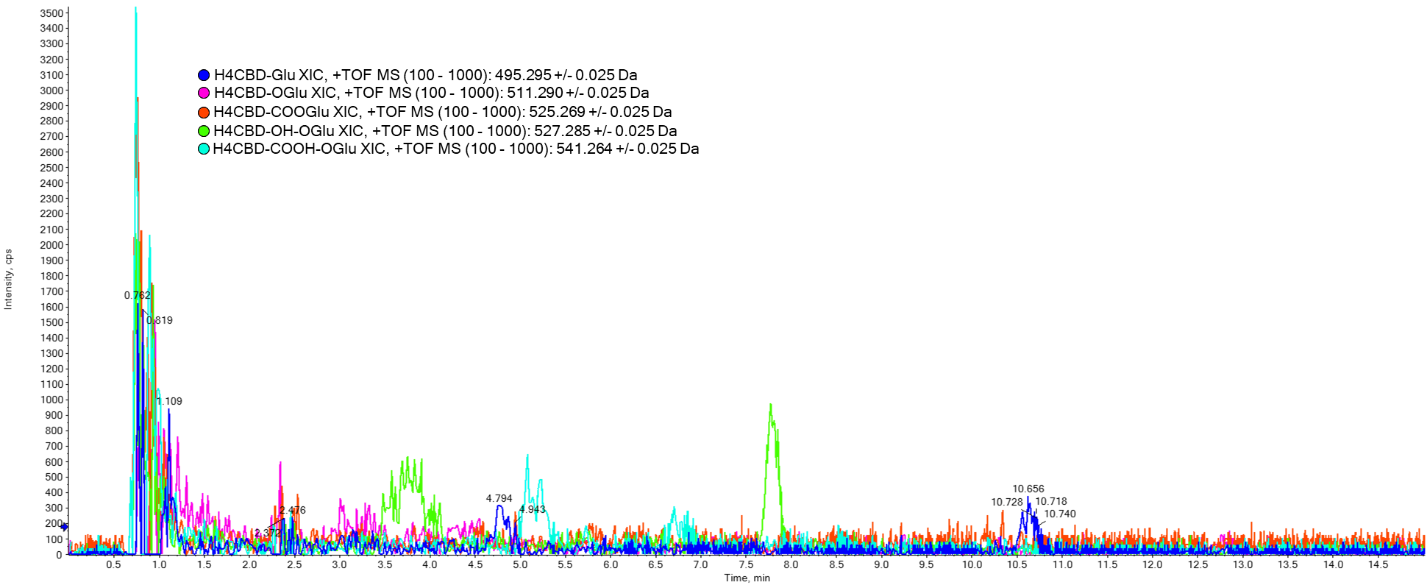


Figure S14: Chromatogram of a urine sample before ingestion of H4CBD, measured on a LC-QqTOF

*
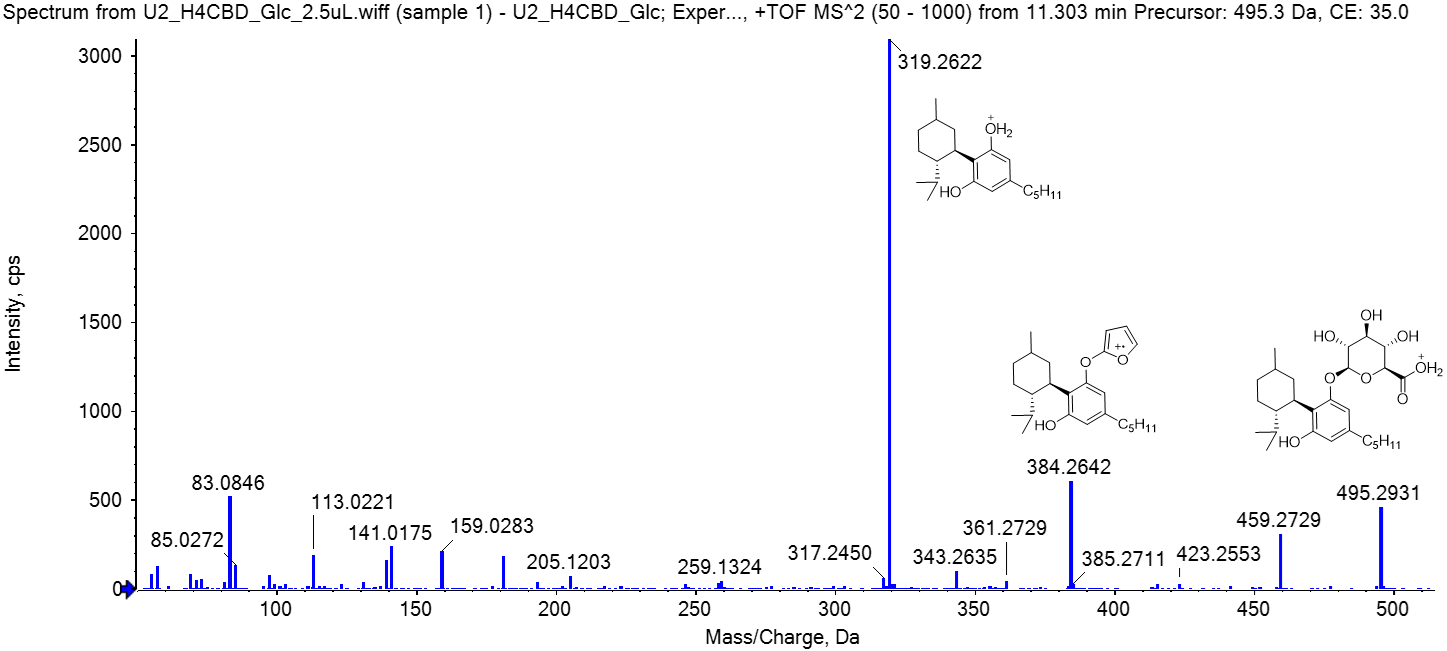
*

Figure S15: Mass spectrum of metabolite M11, a glucuronidated metabolite of H4CBD (from a urine sample 3 h after ingestion of 25 mg H4CBD).


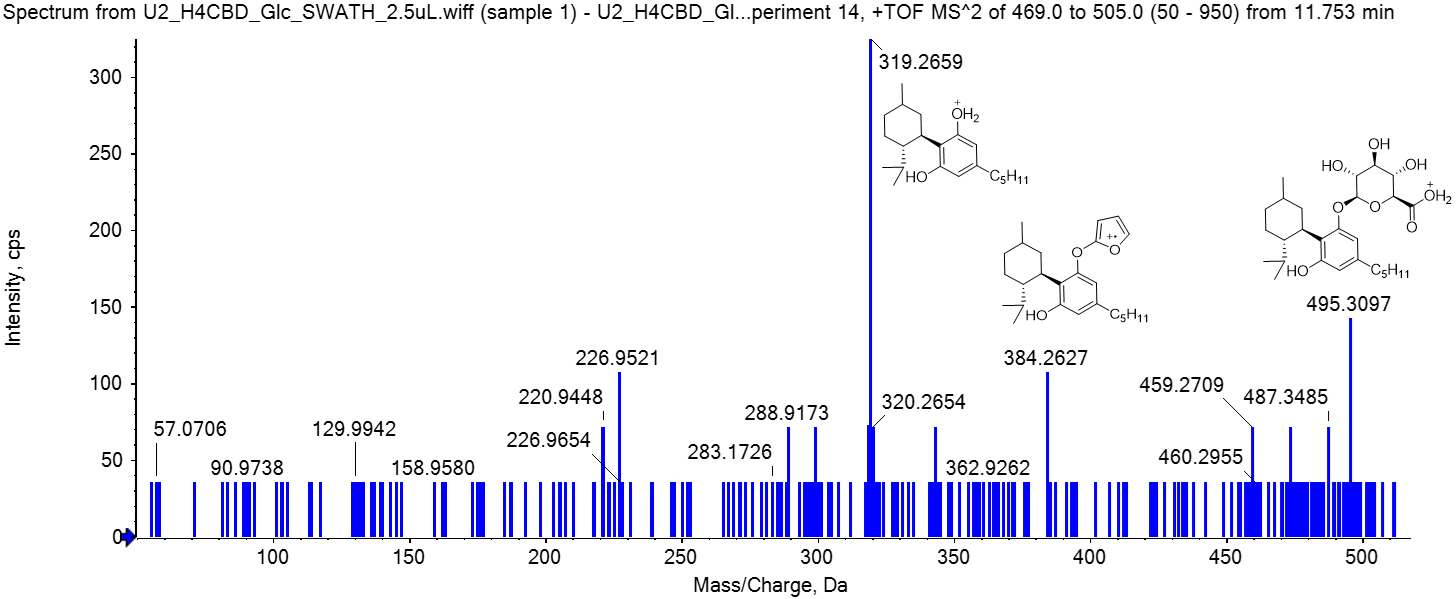


Figure S16: Mass spectrum of metabolite M12, a glucuronidated metabolite of H4CBD (from a urine sample 3 h after ingestion of 25 mg H4CBD). Acquired in SWATH mode.


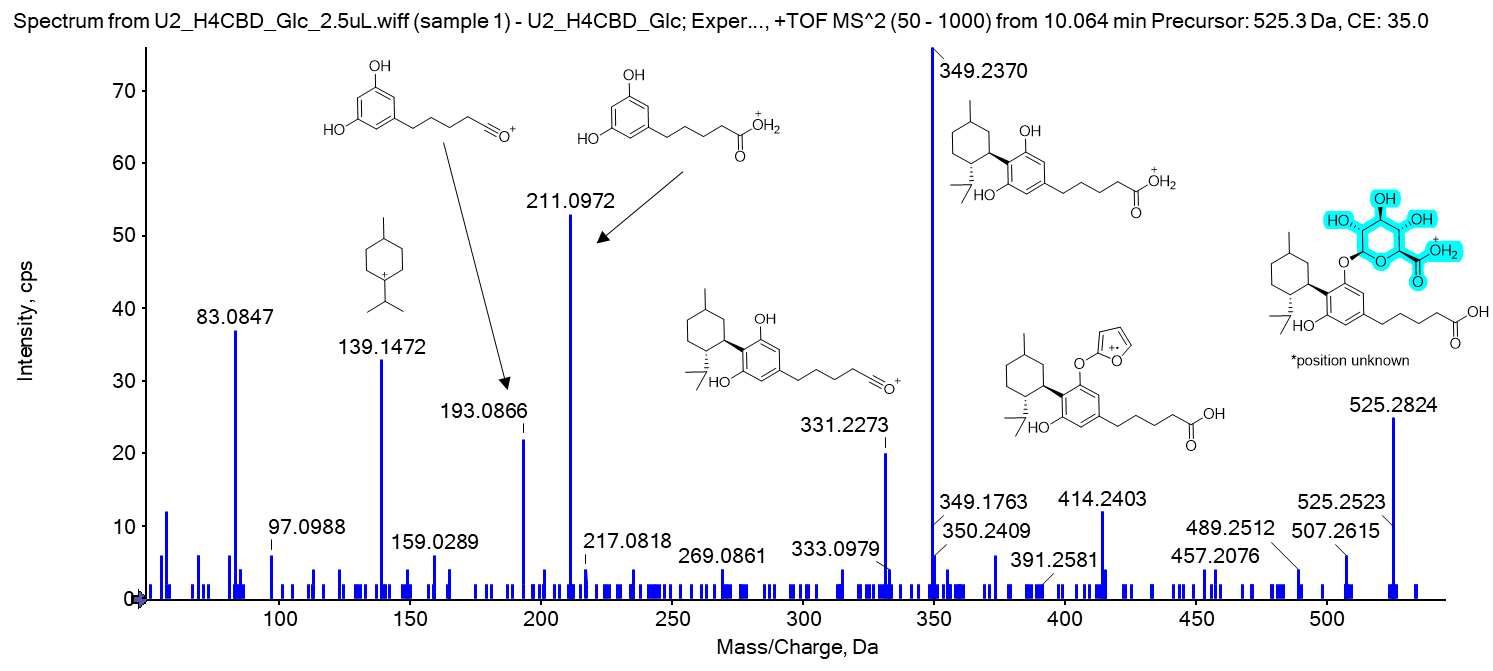


Figure S17: Mass spectrum of metabolite M13, a carboxylated and glucuronidated metabolite of H4CBD (from a urine sample 3 h after ingestion of 25 mg H4CBD). The position of the glucuronide moiety is unknown.


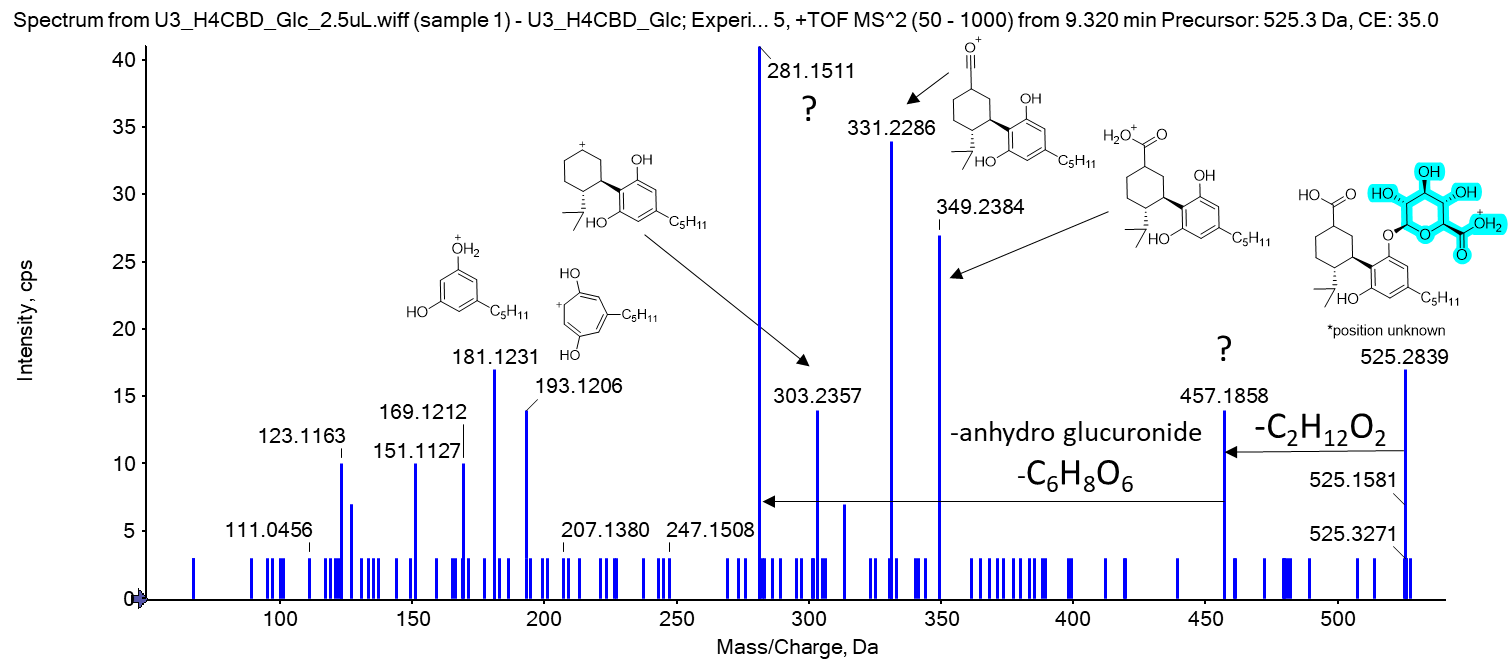


Figure S18: Mass spectrum of metabolite M14, a carboxylated and glucuronidated metabolite of H4CBD (from a urine sample 3 h after ingestion of 25 mg H4CBD). The position of the glucuronide moiety is unknown.


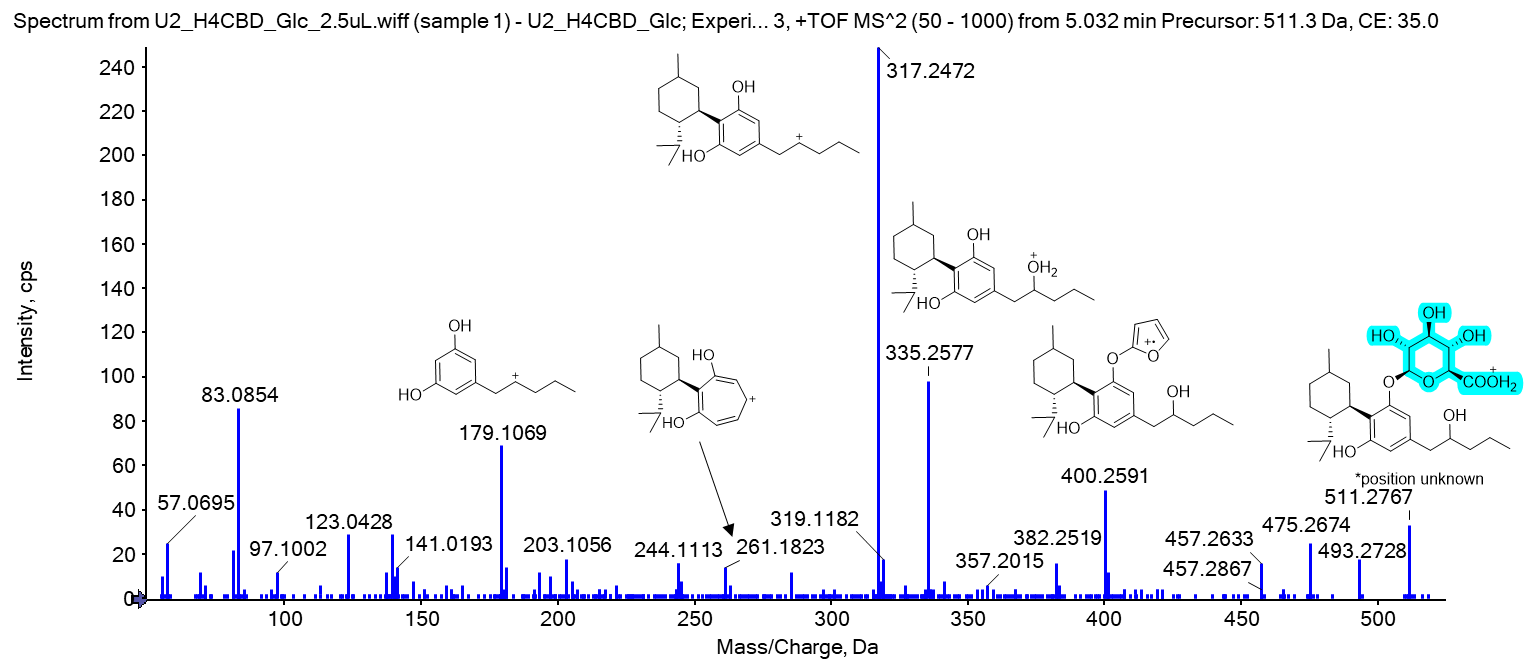


Figure S19: Mass spectrum of metabolite M15, a hydroxylated and glucuronidated metabolite of H4CBD (from a urine sample 3 h after ingestion of 25 mg H4CBD). The position of the glucuronide moiety is unknown.


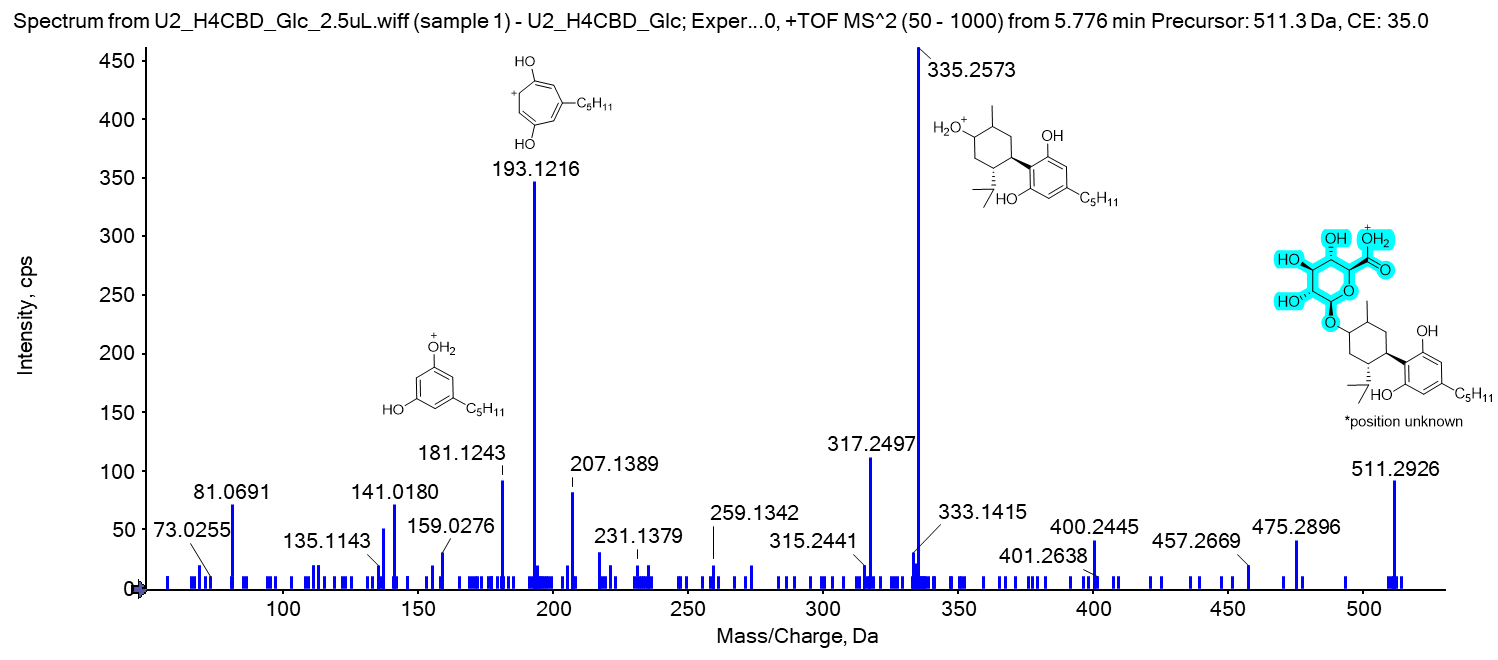


Figure S20: Mass spectrum of metabolite M16, a hydroxylated and glucuronidated metabolite of H4CBD (from a urine sample 3 h after ingestion of 25 mg H4CBD). The position of the glucuronide moiety and the hydroxy group are unknown.


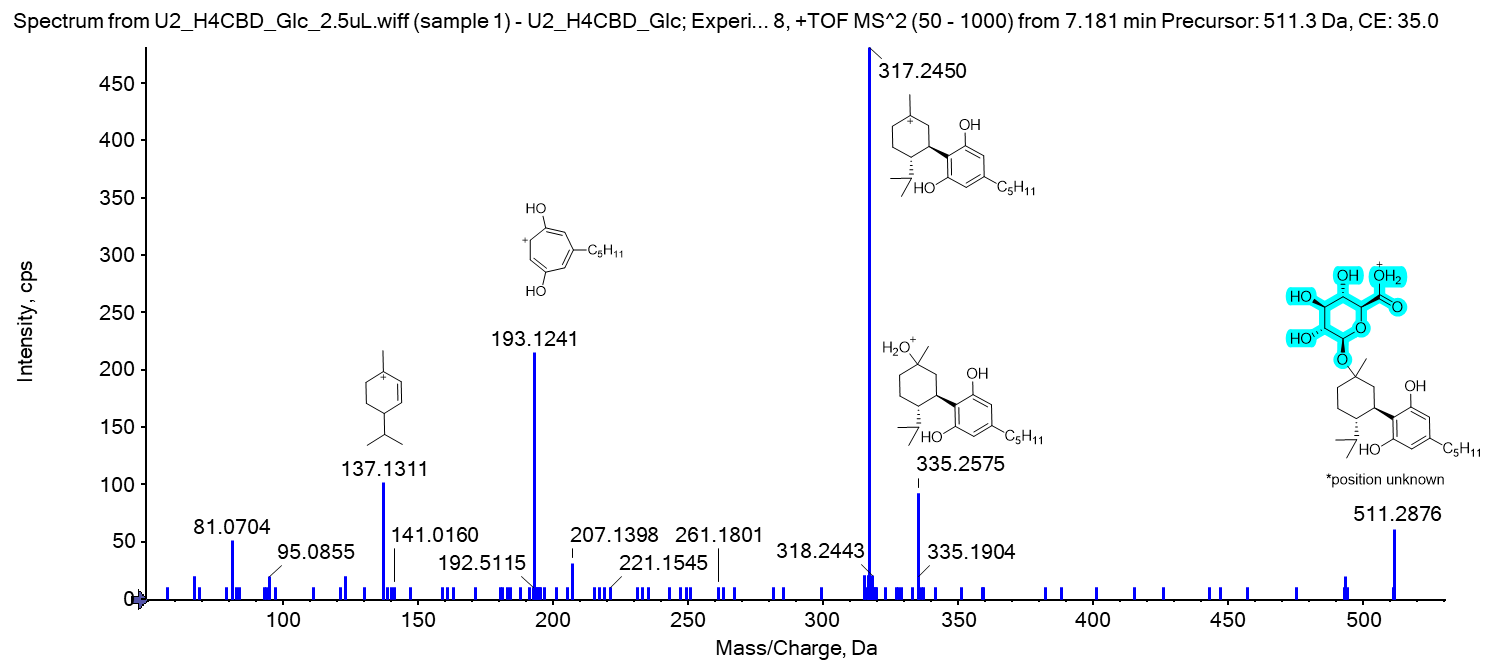


Figure S21: Mass spectrum of metabolite M17, a hydroxylated and glucuronidated metabolite of H4CBD (from a urine sample 3 h after ingestion of 25 mg H4CBD). The position of the glucuronide moiety and the hydroxy group is unknown.


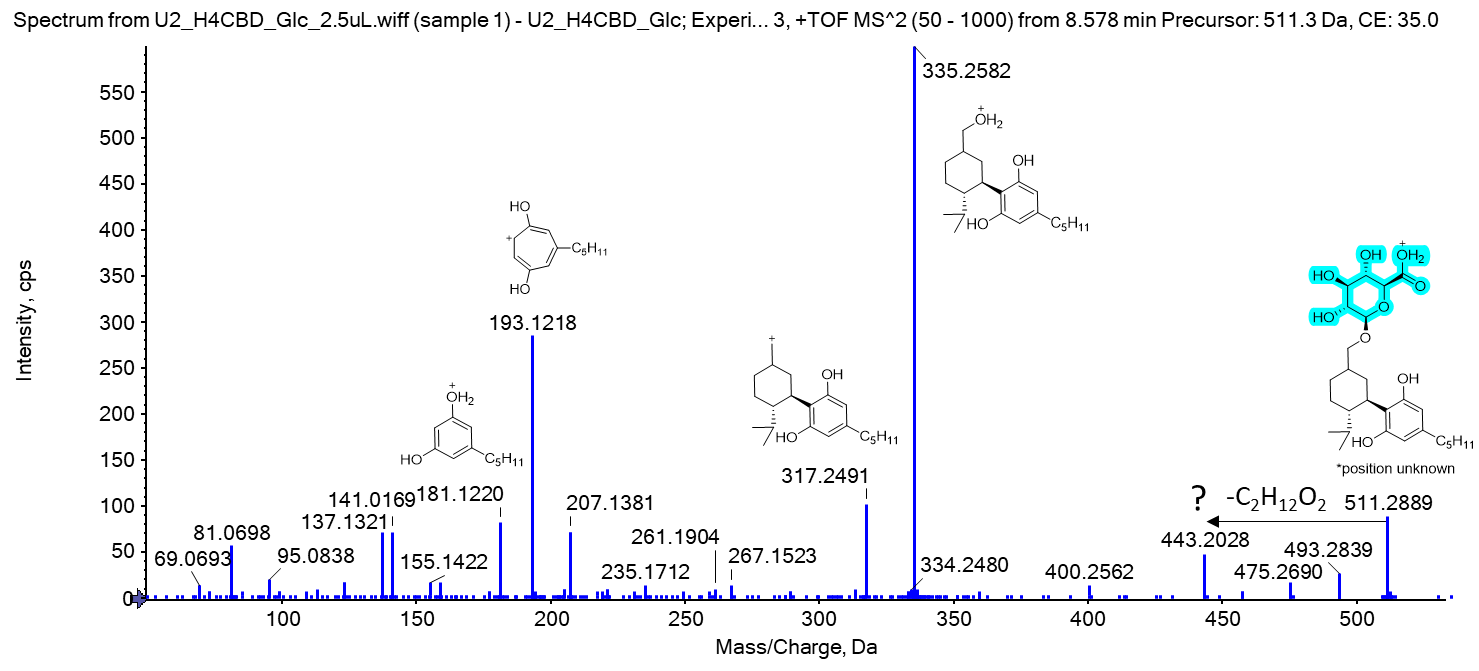


Figure S22: Mass spectrum of metabolite M18, a hydroxylated and glucuronidated metabolite of H4CBD (from a urine sample 3 h after ingestion of 25 mg H4CBD). The position of the glucuronide moiety is unknown.


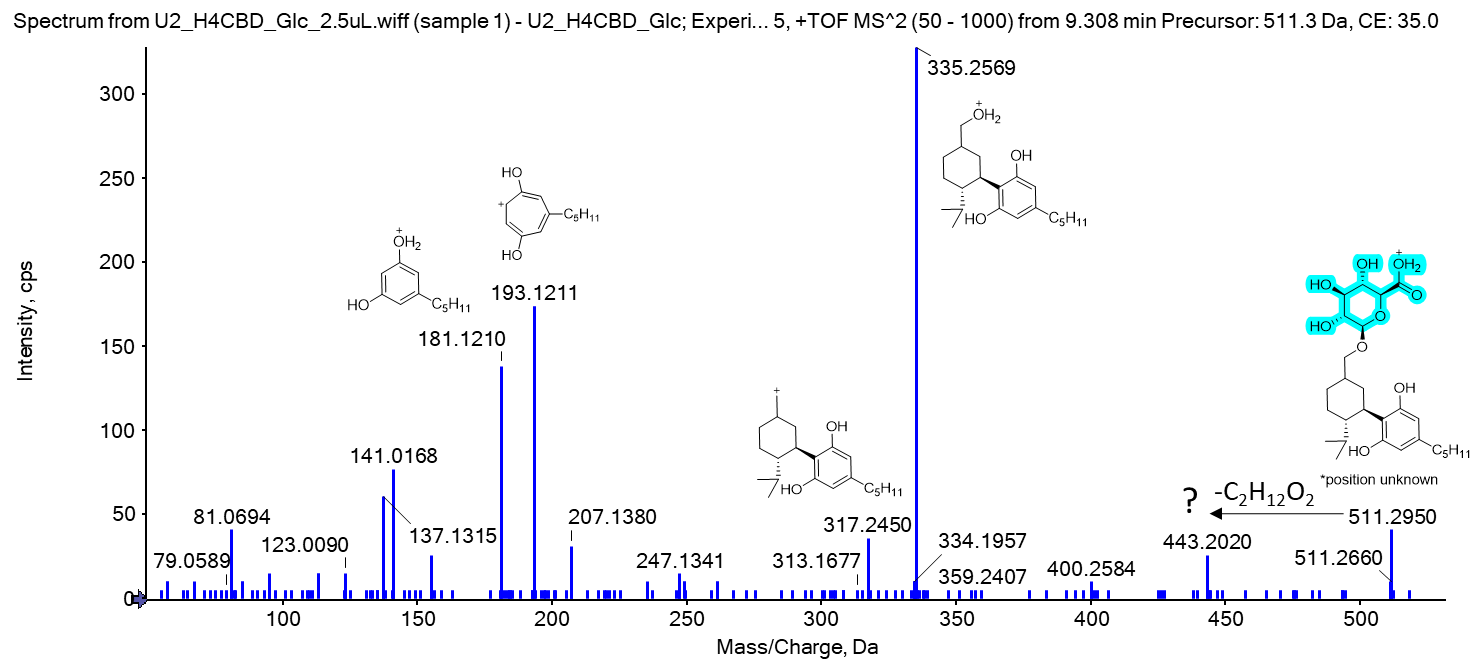


Figure S23: Mass spectrum of metabolite M19, a hydroxylated and glucuronidated metabolite of H4CBD (from a urine sample 3 h after ingestion of 25 mg H4CBD). The position of the glucuronide moiety is unknown.


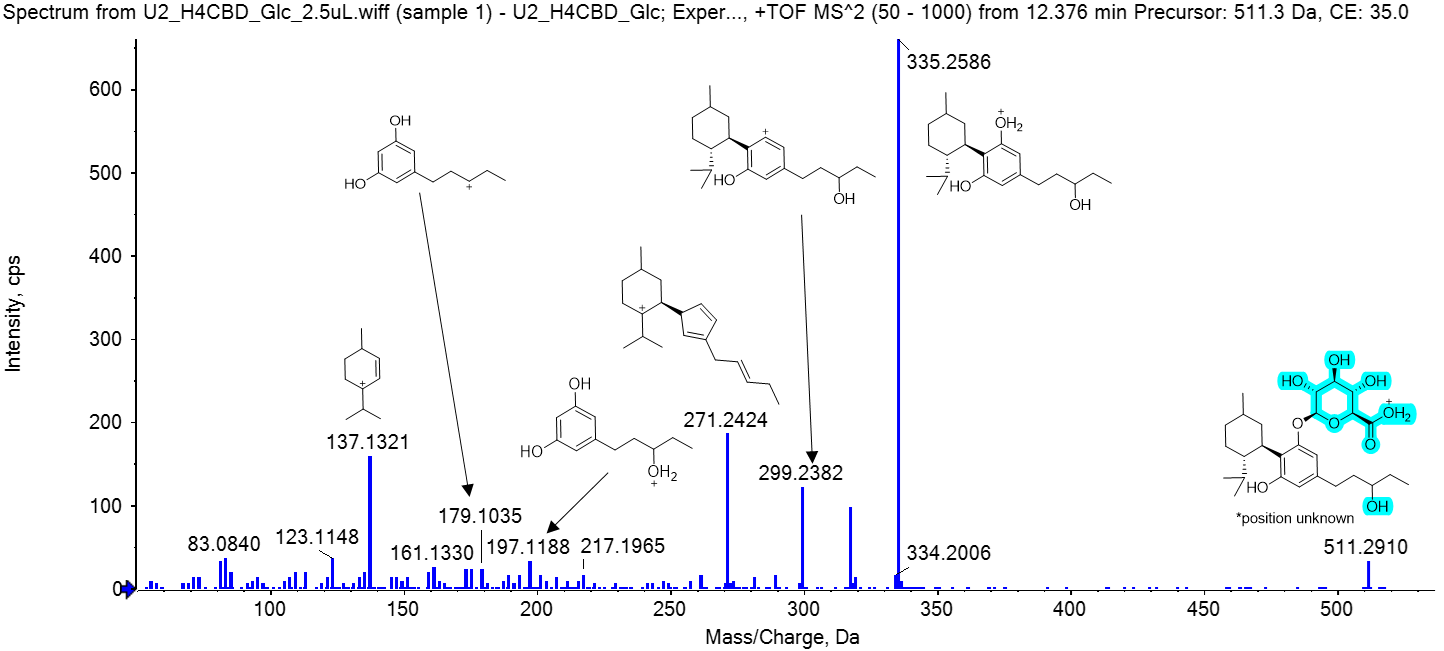


Figure S24: Mass spectrum of metabolite M20, a hydroxylated and glucuronidated metabolite of H4CBD (from a urine sample 3 h after ingestion of 25 mg H4CBD). The position of the hydroxy group and the glucuronide are unknown.


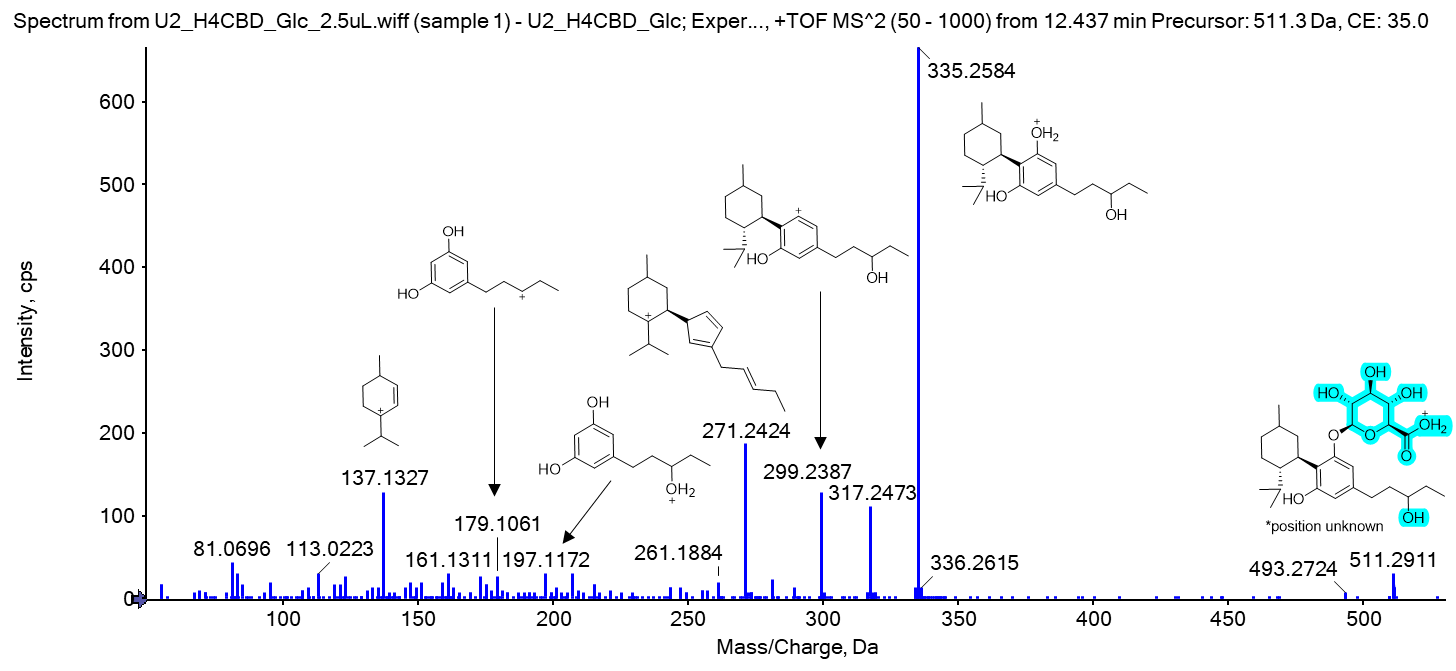


Figure S25: Mass spectrum of metabolite M21, a hydroxylated and glucuronidated metabolite of H4CBD (from a urine sample 3 h after ingestion of 25 mg H4CBD). The position of the hydroxy group and the glucuronide are unknown.


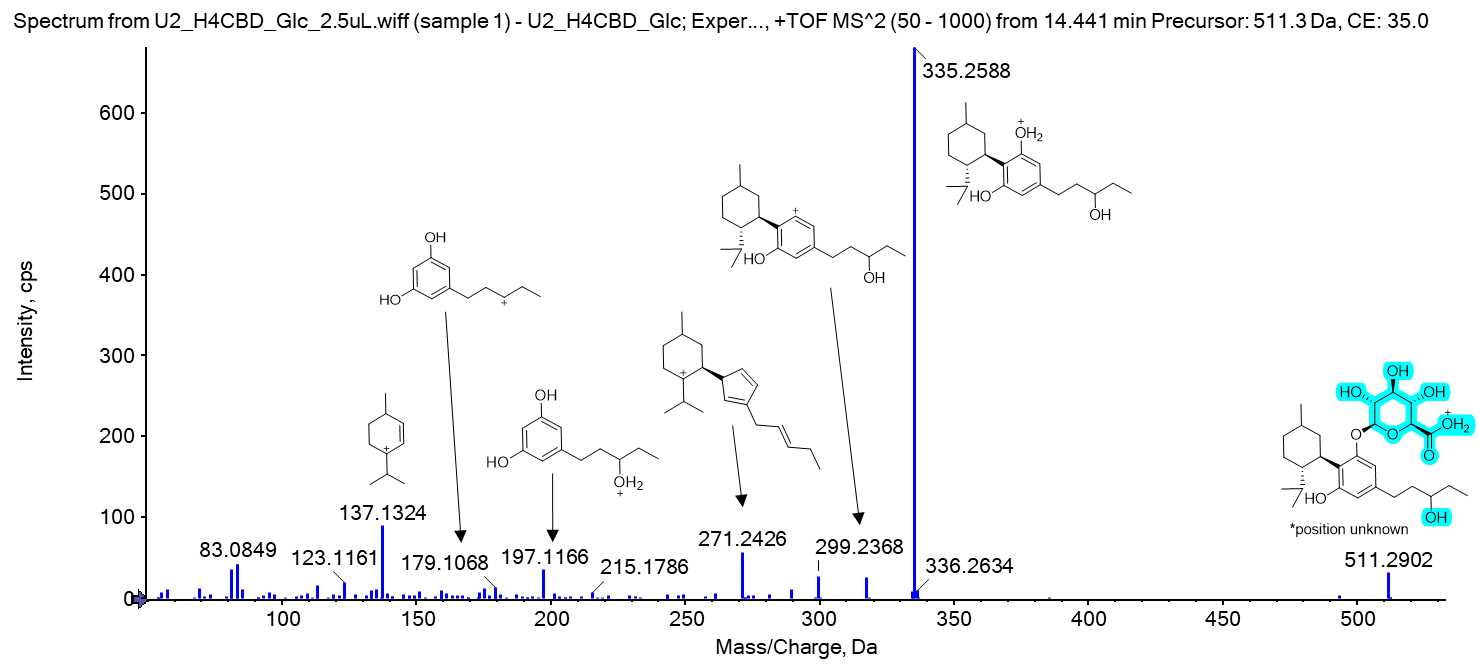


Figure S26: Mass spectrum of metabolite M22, a hydroxylated and glucuronidated metabolite of H4CBD (from a urine sample 3 h after ingestion of 25 mg H4CBD). The position of the hydroxy group and the glucuronide are unknown.


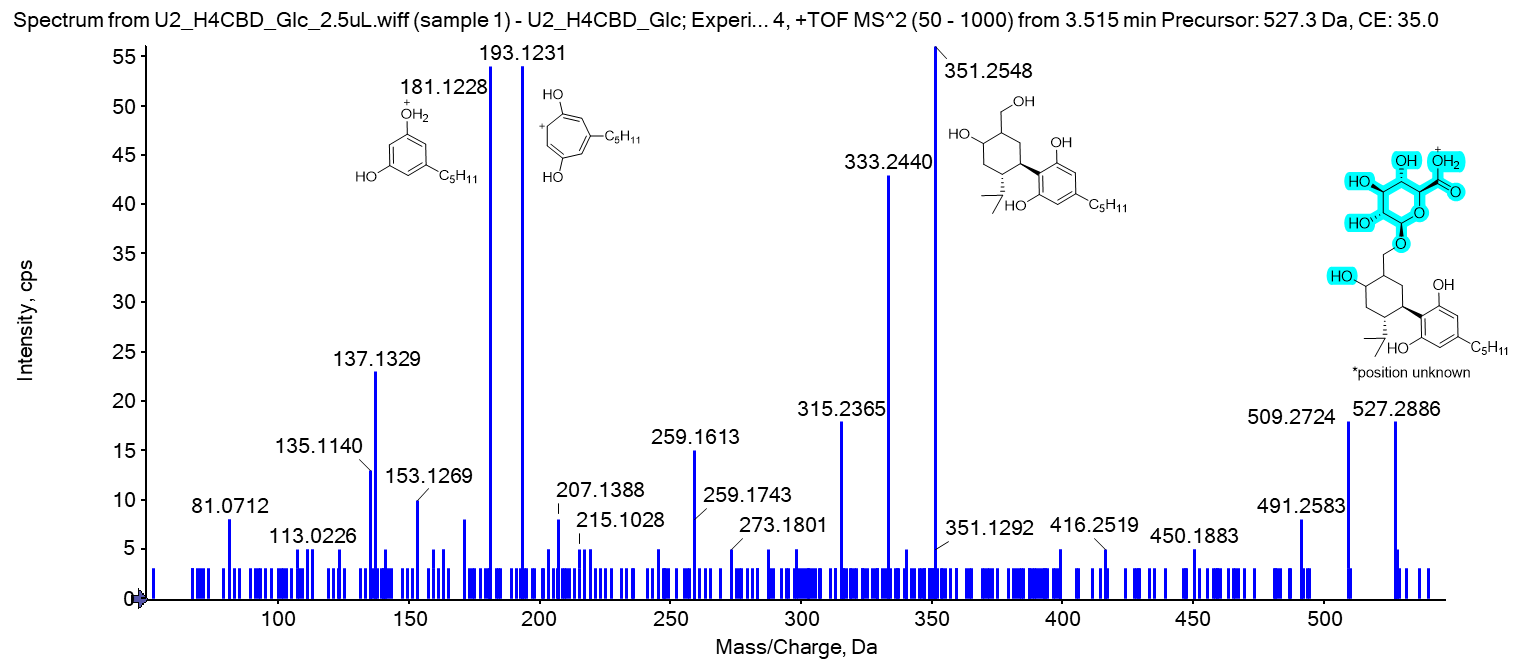


Figure S27: Mass spectrum of metabolite M23, a bishydroxylated and glucuronidated metabolite of H4CBD (from a urine sample 3 h after ingestion of 25 mg H4CBD). Both hydroxylation positions are found on the alicyclic moiety, their position and the position of the glucuronide are not clear.


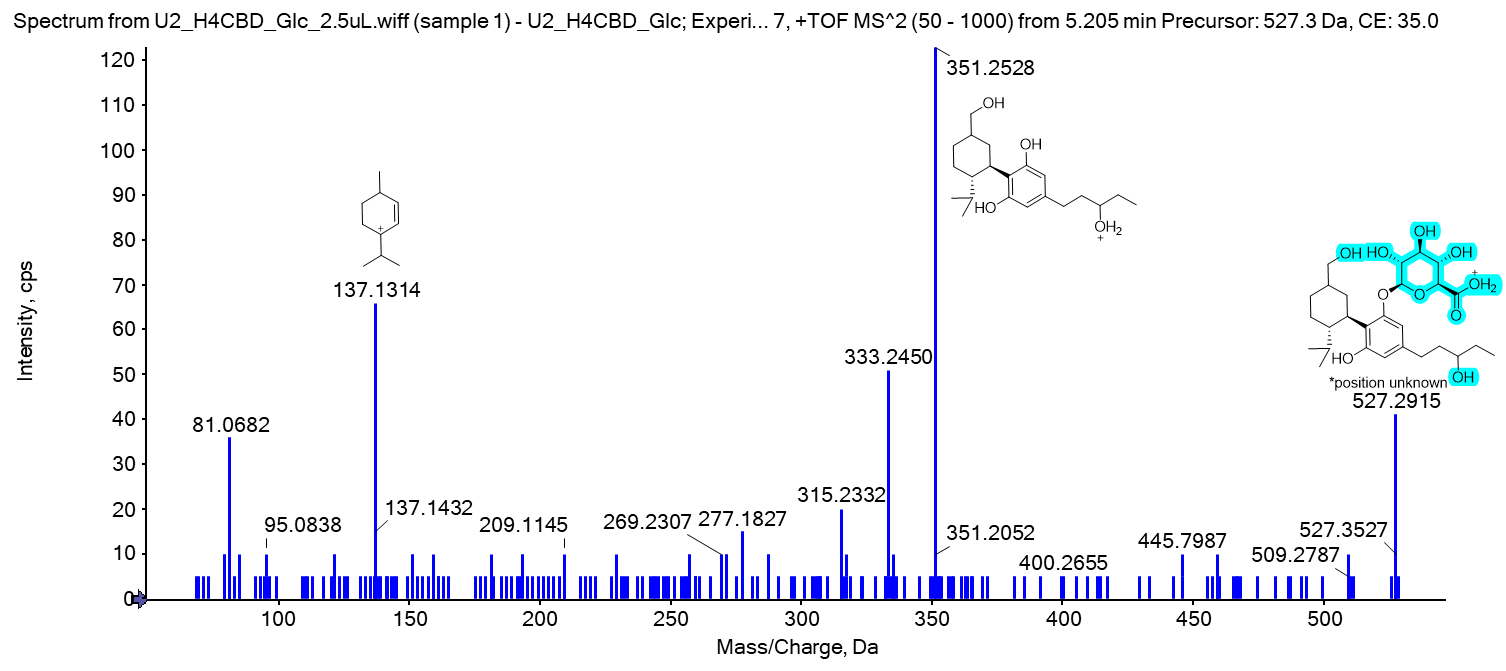


Figure S28: Mass spectrum of metabolite M24, a bishydroxylated and glucuronidated metabolite of H4CBD (from a urine sample 3 h after ingestion of 25 mg H4CBD). One hydroxylation positions is found on the alicyclic moiety, the other hydroxylation position is on the side-chain, their position and the position of the glucuronide are not clear.


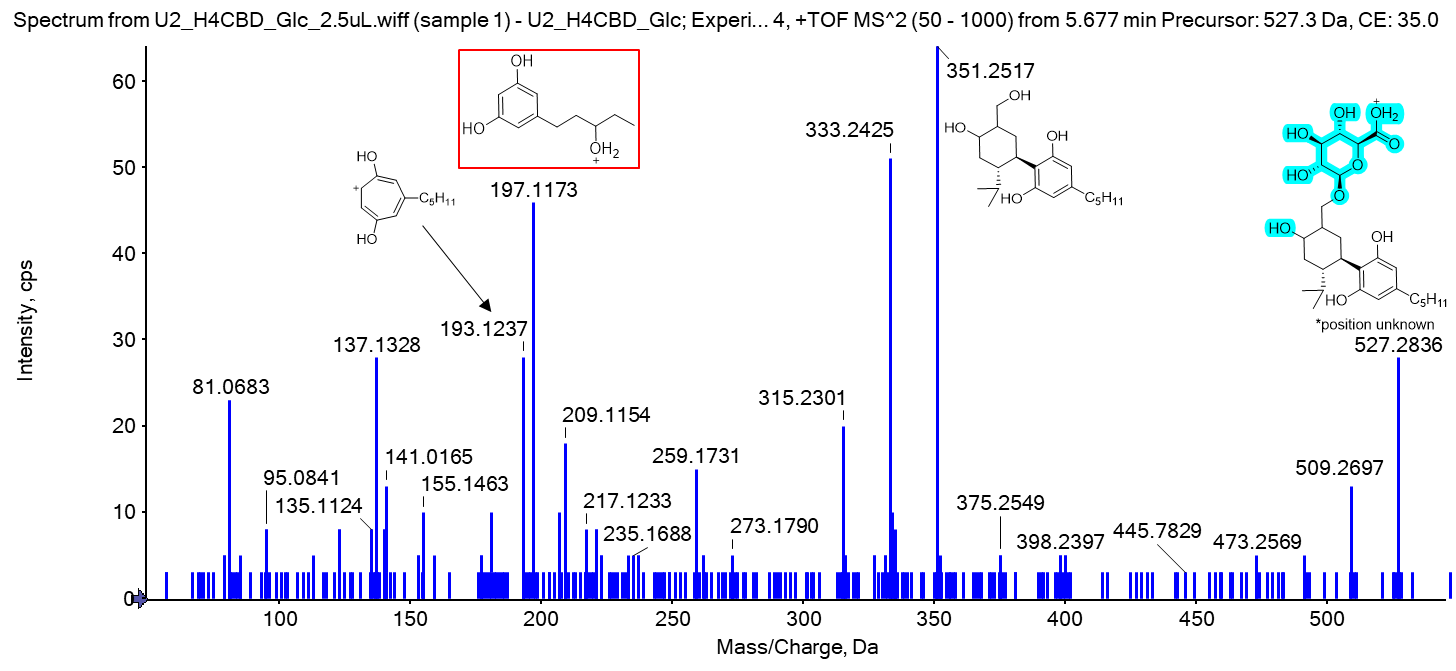


Figure S29: Mass spectrum of metabolite M25, a bishydroxylated and glucuronidated metabolite of H4CBD (from a urine sample 3 h after ingestion of 25 mg H4CBD). Both hydroxylation positions are found on the alicyclic moiety, their position and the position of the glucuronide are not clear. The ion m/z 197.1173 could result from a coeluting metabolite with a hydroxylation position on the side-chain.


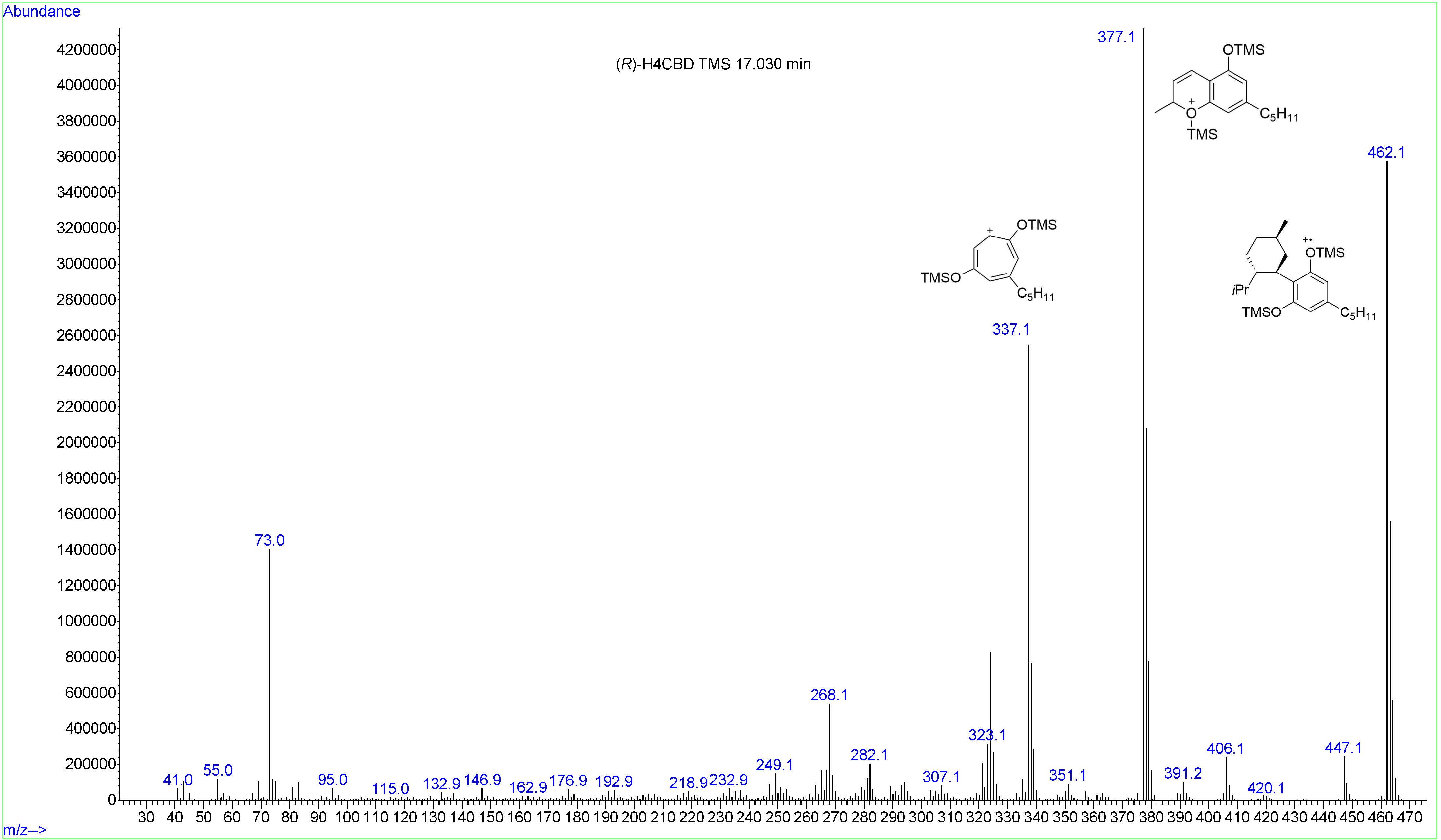


Figure S30: EI mass spectrum of the metabolite M26 (R)-H4CBD TMS


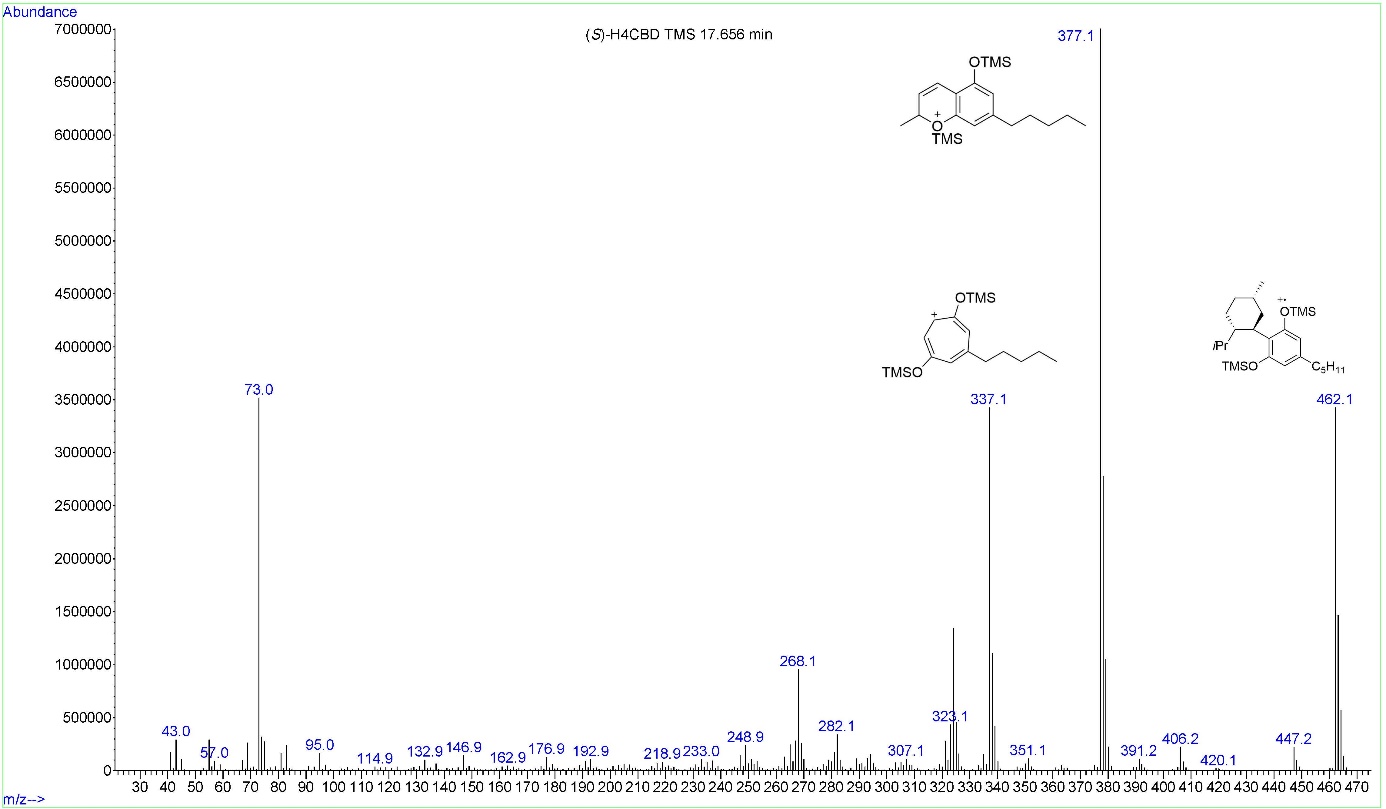


Figure S31: EI mass spectrum of the metabolite M27 (S)-H4CBD TMS, the same ions are formed as for (R)-H4CBD TMS.


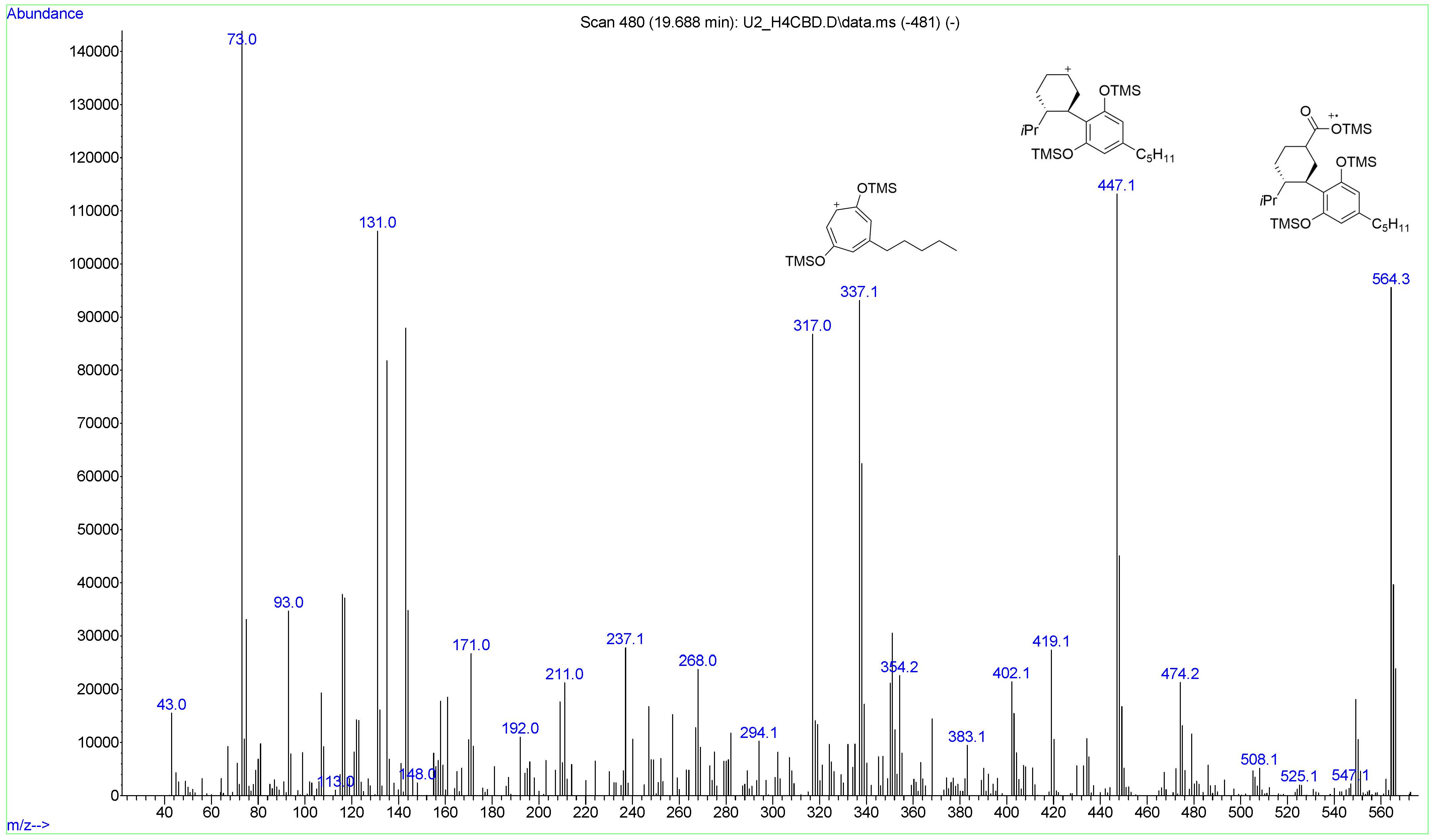


Figure S32: EI mass spectrum of the carboxylated metabolite M28 7-COOH-H4CBD TMS.


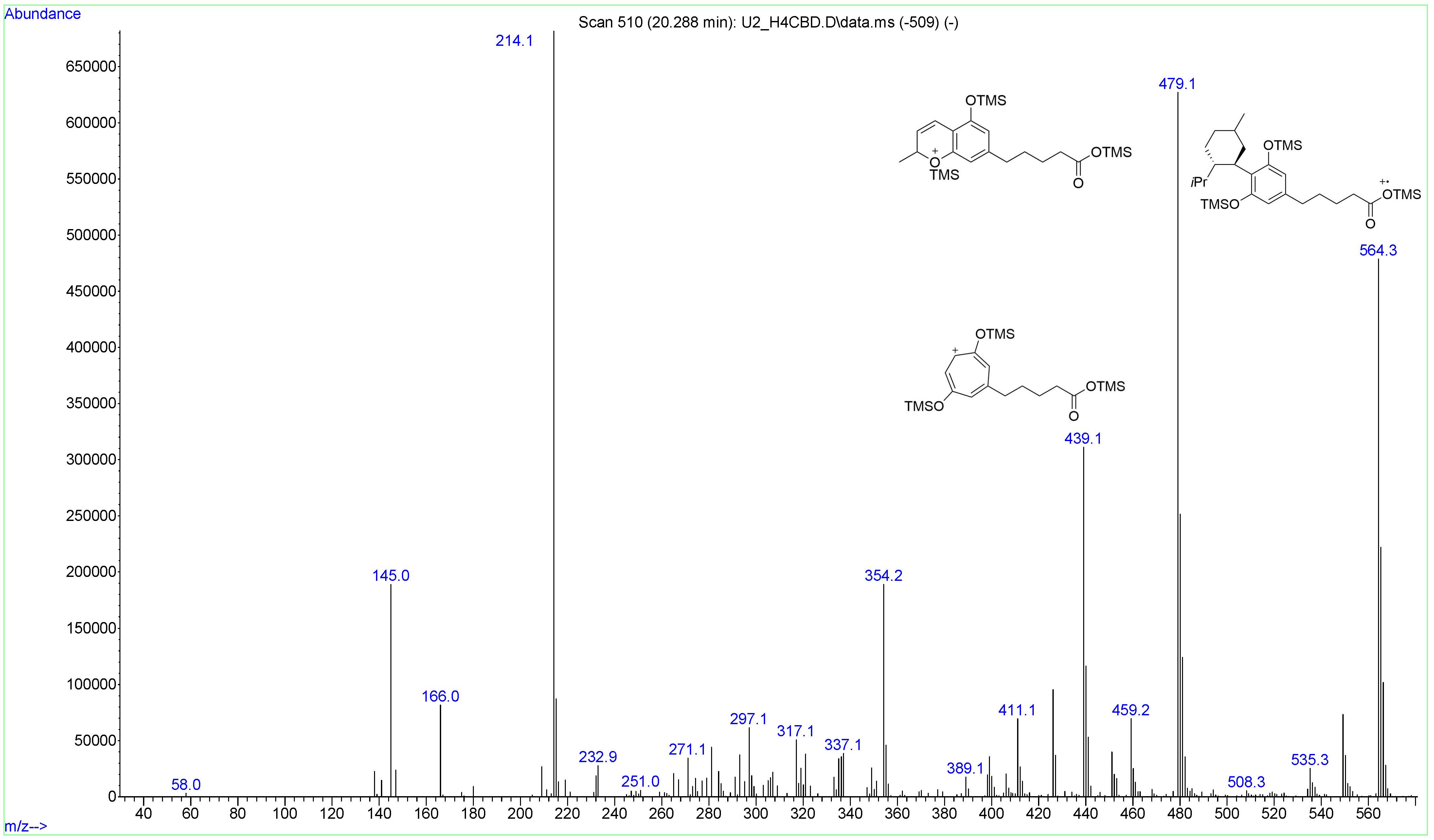


Figure S33: EI mass spectrum of the carboxylated metabolite M29 5''-COOH-H4CBD TMS.


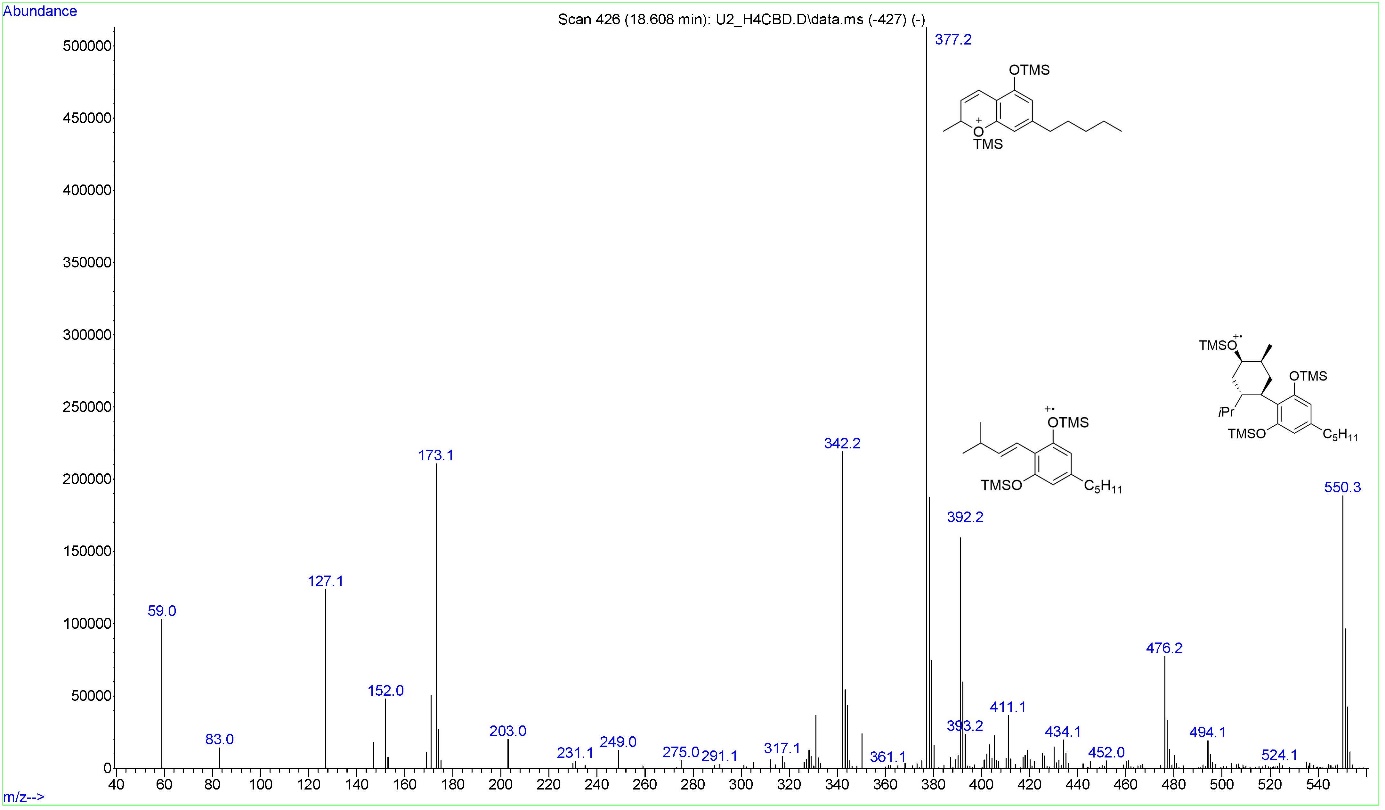


Figure S34: EI mass spectrum of the hydroxylated metabolite M30, hydroxylated on the alicyclic moiety.


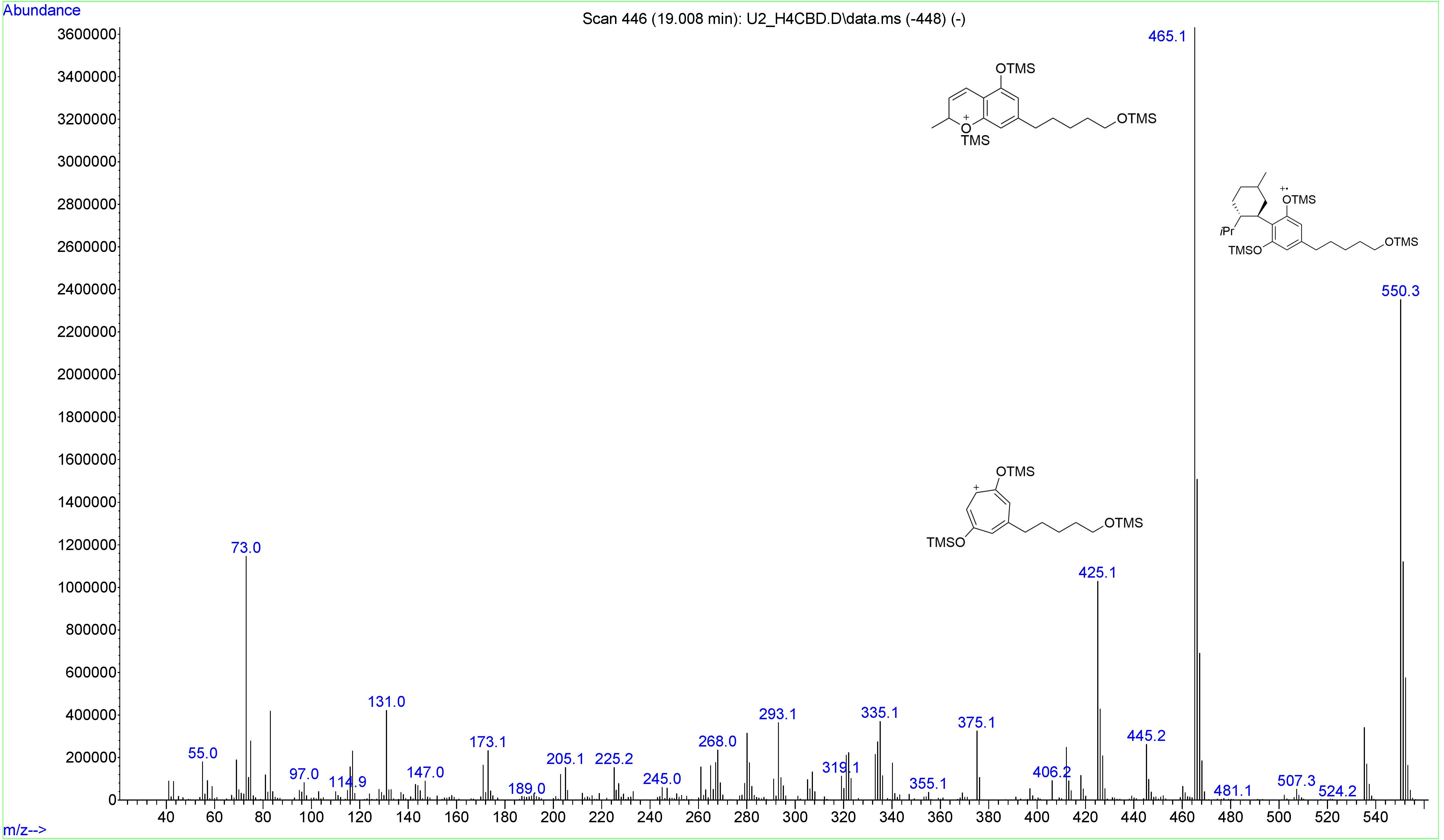


Figure S35: EI mass spectrum of the hydroxylated metabolite M31, hydroxylated on the side-chain. Hydroxylation position unknown.


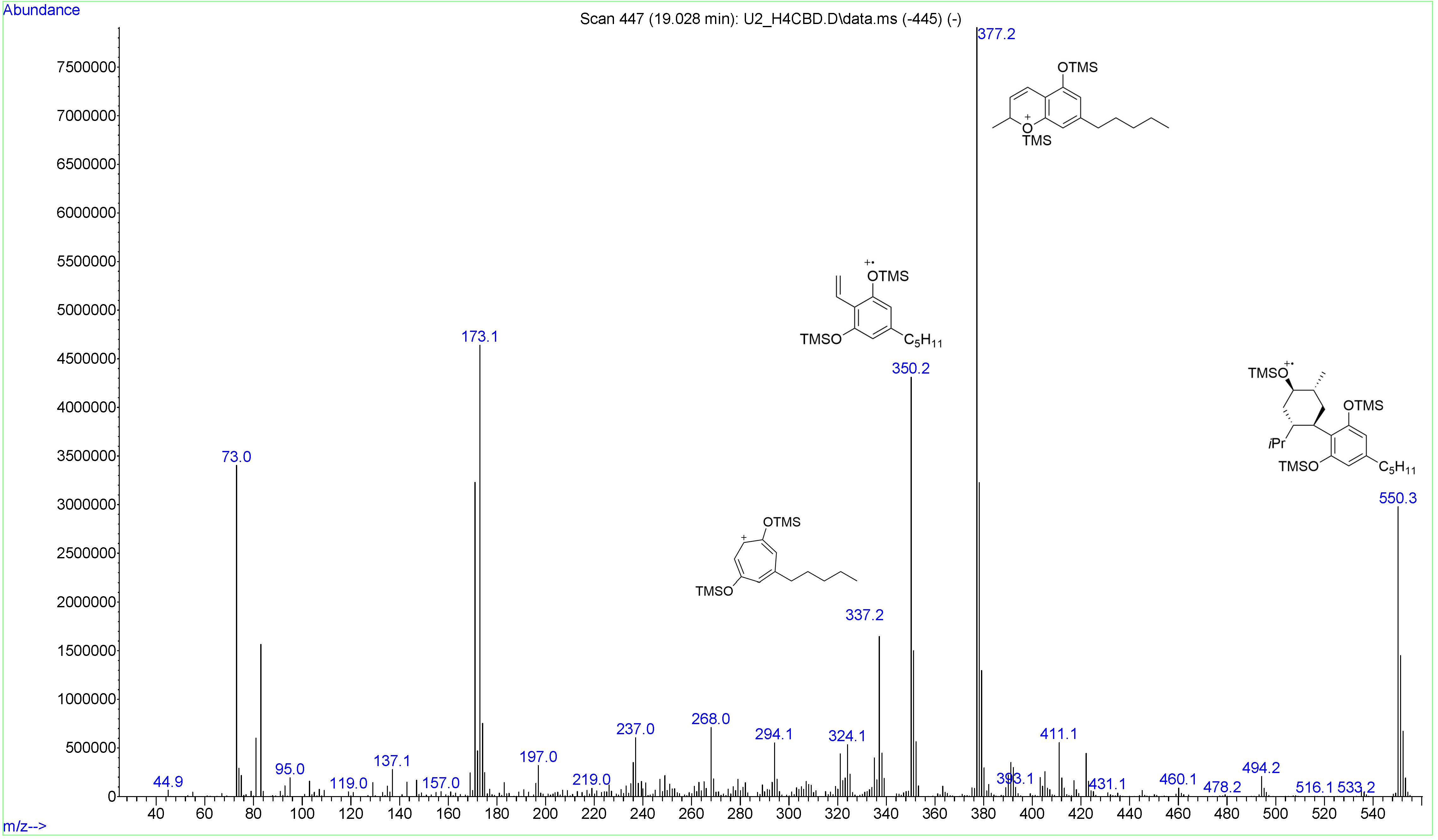


Figure S36: EI mass spectrum of the hydroxylated metabolite M32, hydroxylated on the alicyclic moiety.


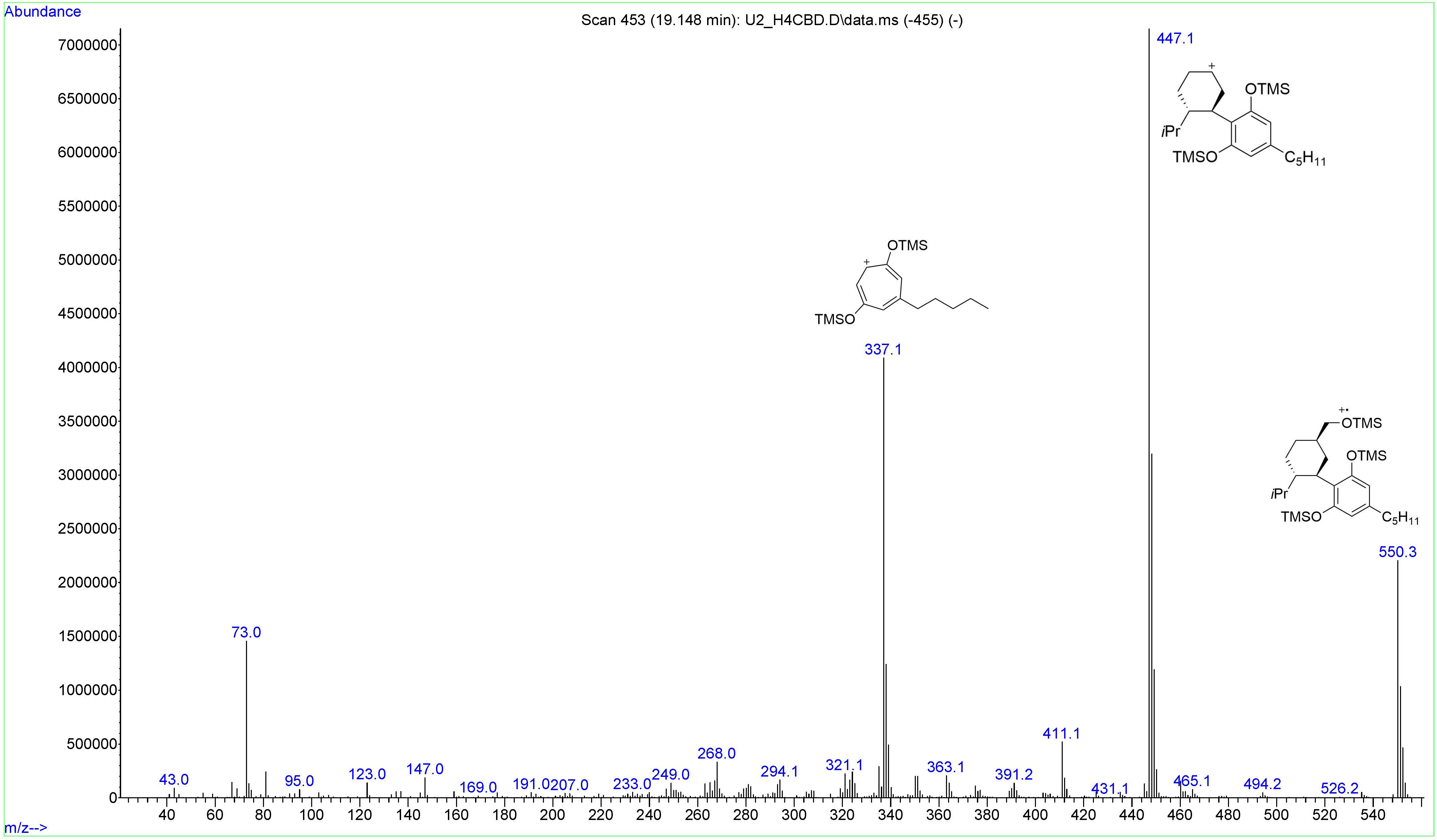


Figure S37: EI mass spectrum of the hydroxylated metabolite M33, hydroxylated on the alicyclic moiety. Presumably 7-OH-(R)-H4CBD


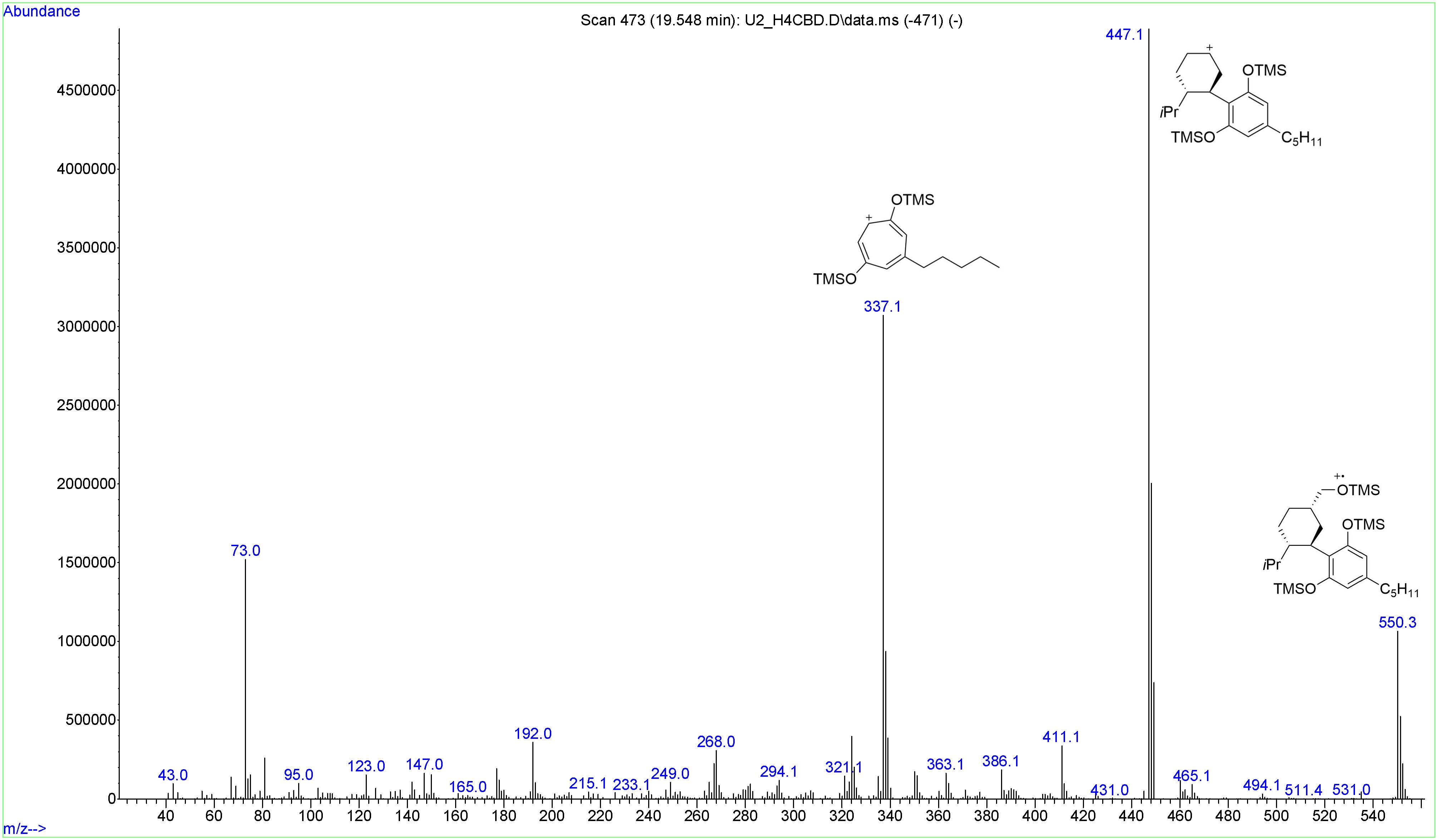


Figure S38: EI mass spectrum of the hydroxylated metabolite M34, hydroxylated on the alicyclic moiety. Presumably 7-OH-(S)-H4CBD


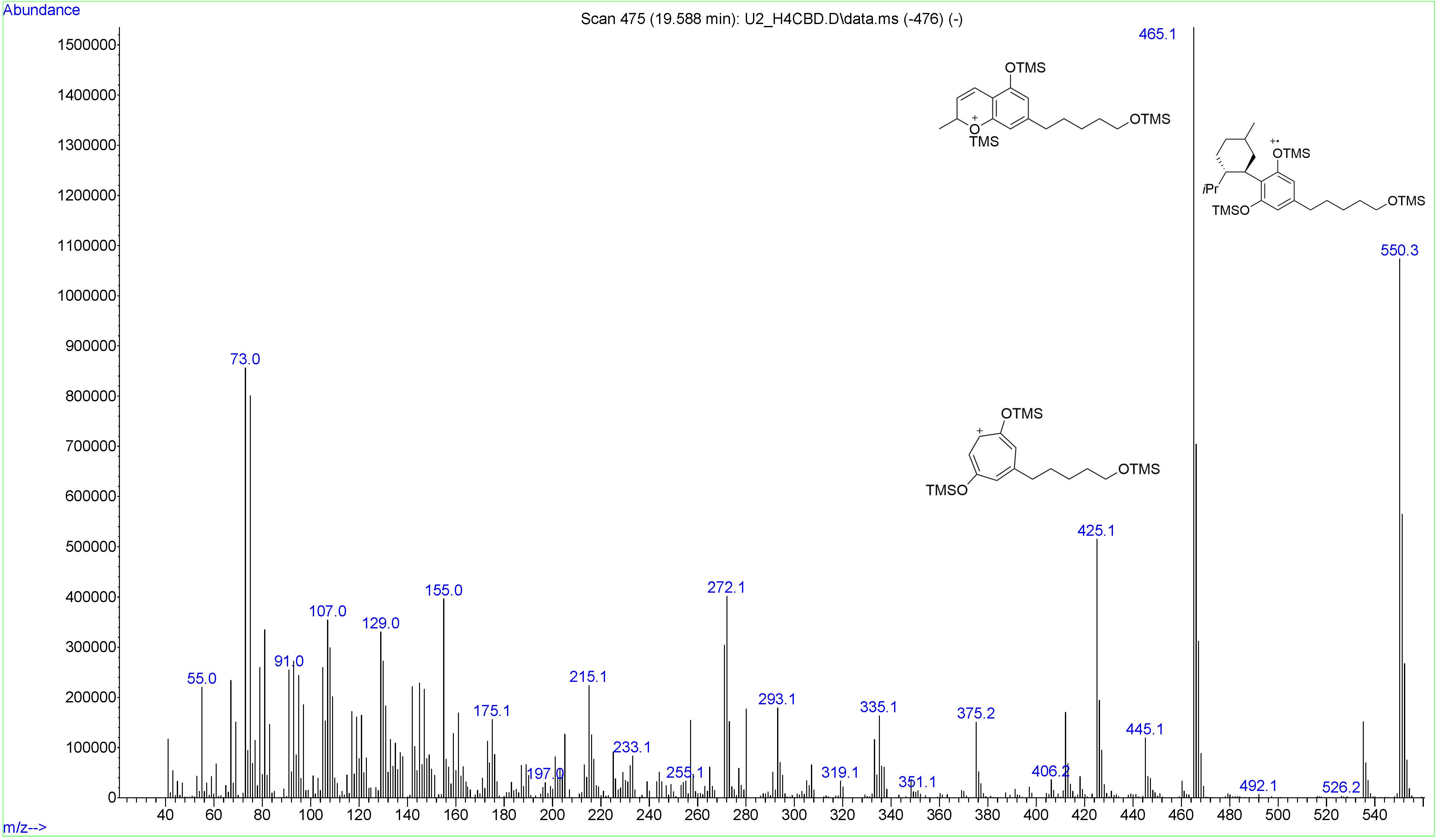


Figure S39: EI mass spectrum of the hydroxylated metabolite M35, hydroxylated on the side-chain. Hydroxylation position unknown.


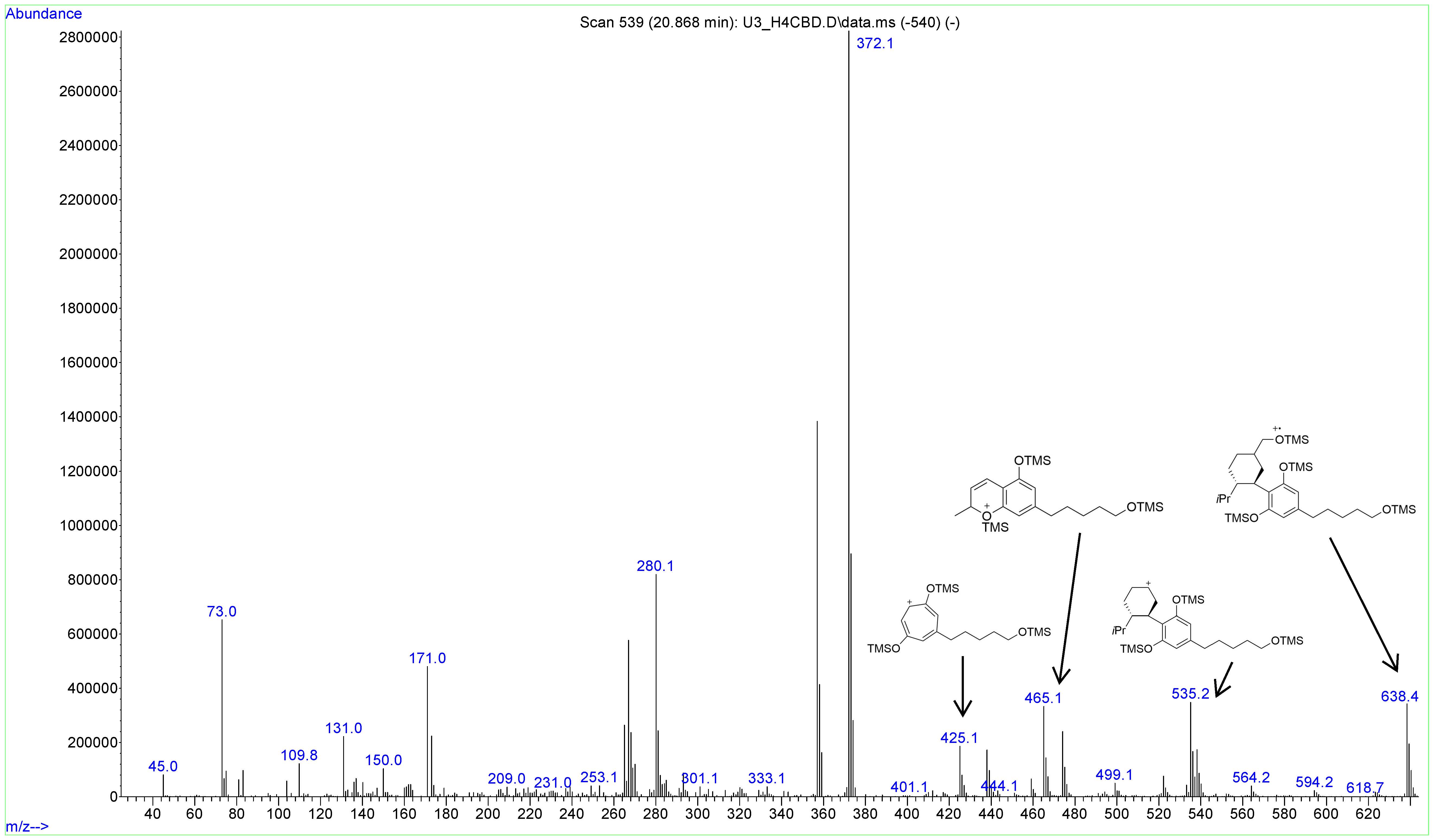


Figure S40: EI mass spectrum of the bishydroxylated metabolite M36, hydroxylated on C7 of the alicyclic moiety and on the side-chain. Position of the side-chain hydroxylation is unknown.


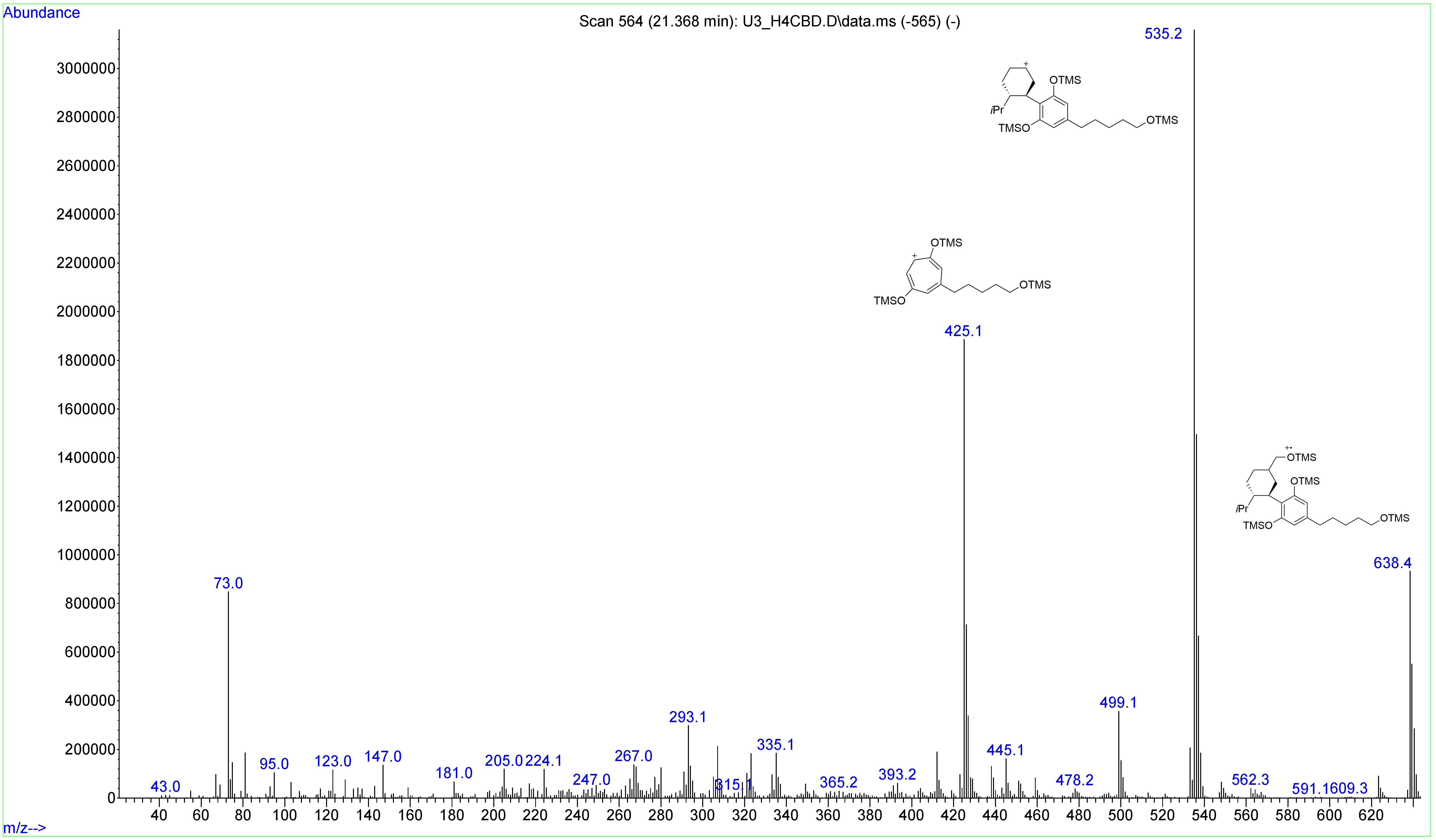


Figure S41: EI mass spectrum of the bishydroxylated metabolite M37, hydroxylated on C7 of the alicyclic moiety and on the side-chain. Position of the side-chain hydroxylation is unknown.

Figure S42: Product ion spectrum of 7-COOH-CBD at a collision energy of +46 V.

Figure S43: Product ion spectrum of 7-COOH-CBD at a collision energy of +73 V.


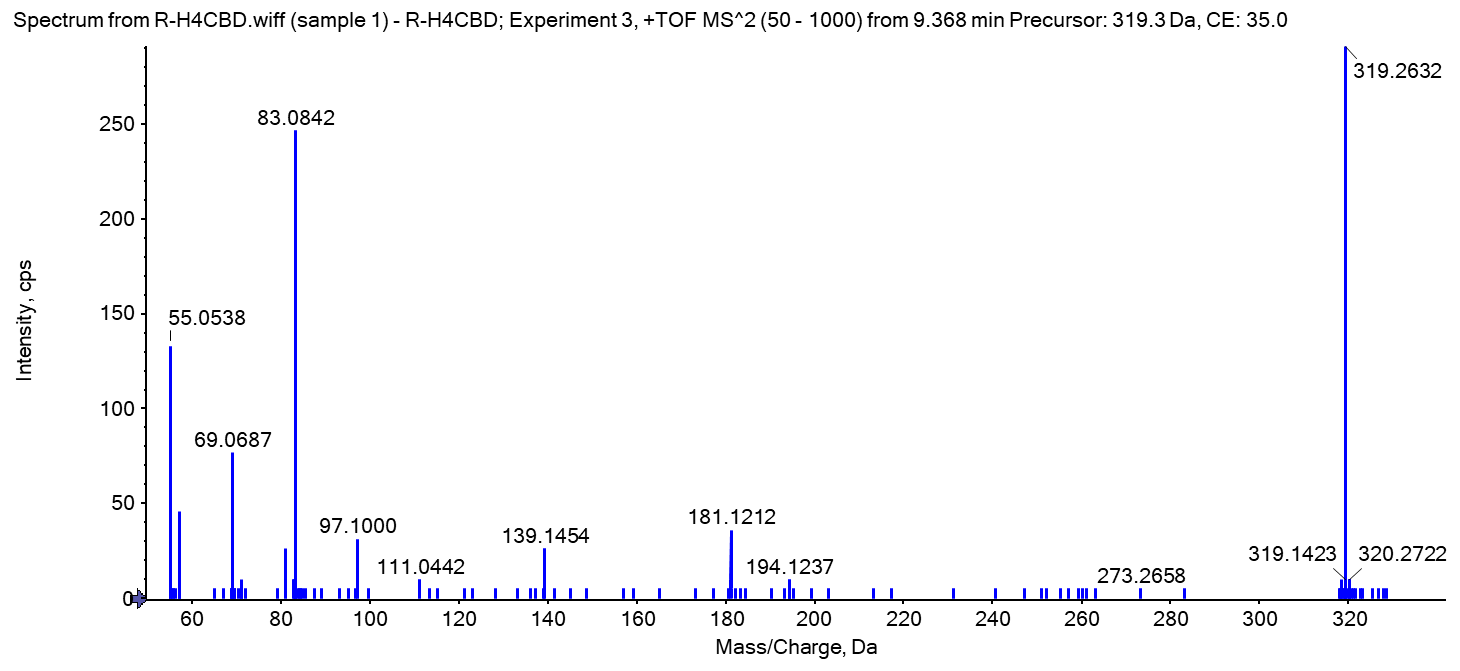


Figure S44: Product ion spectrum of (S)-H4CBD (reference standard).


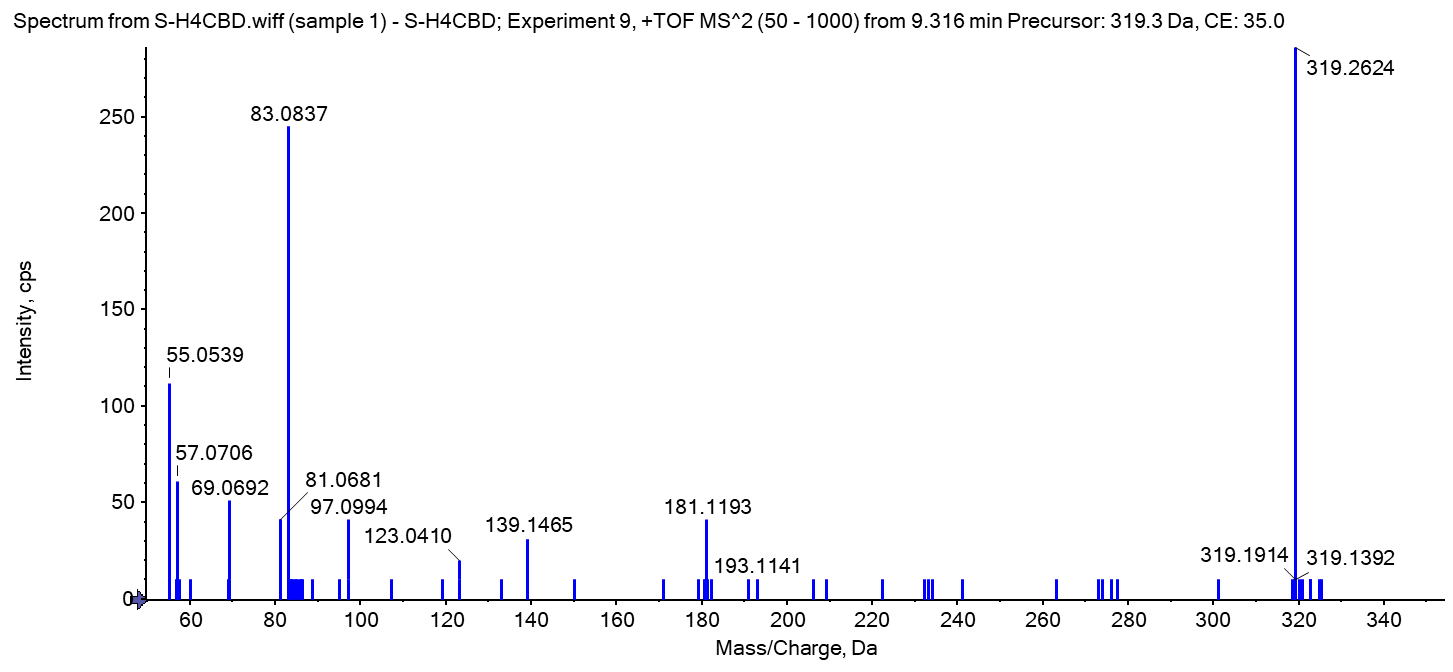


Figure S45: Product ion spectrum of (R)-H4CBD (reference standard).


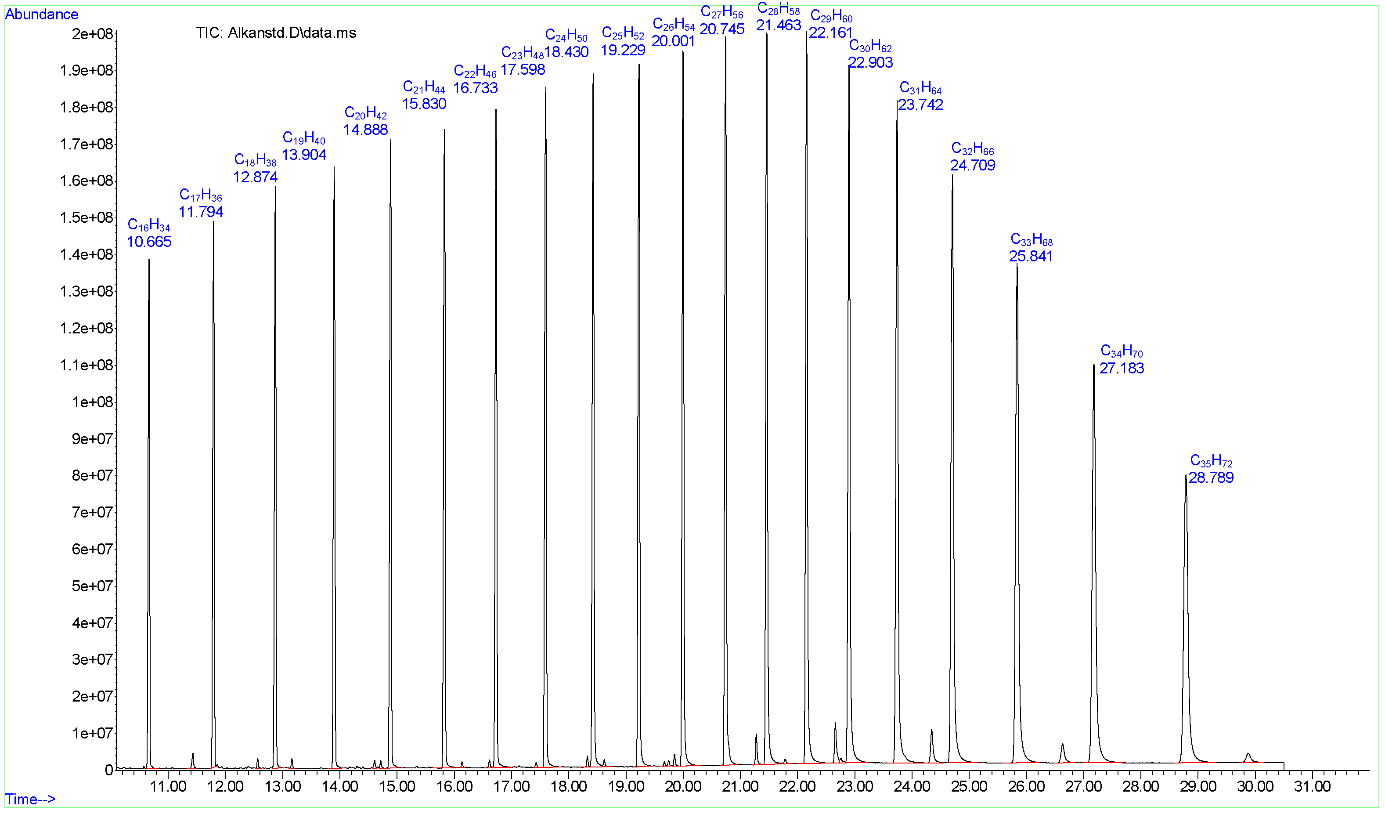


Figure S46: Chromatogram of an n-alkane standard (C7-C40) used for the determination of Kováts indices.
